# The search for scientific meaning in mindfulness research: Insights from a scoping review

**DOI:** 10.1371/journal.pone.0264924

**Published:** 2022-05-04

**Authors:** Nhat Tram Phan-Le, Linda Brennan, Lukas Parker

**Affiliations:** School of Media and Communication, RMIT University, Melbourne, Australia; University of Connecticut, UNITED STATES

## Abstract

There are on-going debates about what is and is not ‘mindfulness’. These debates are stifling rigorous academic research as scientific precision is a precursor to shared meaning. While mindfulness is a growing field of research, these divergent and conflated meanings are limiting deeper interdisciplinary research. Interventions designed in one practice context may not be useful in other contexts because meaning is not transferred between settings. This review clarifies the various research domains that study mindfulness and the conceptual and operational definitions in each domain. This two-stage study comprises a scoping review of mindfulness classifications and a comparative content mapping of mindfulness studies from 2015 to 2021. The initial comprehensive search strategy followed the preferred reporting items for scoping reviews and meta-analysis (PRISMA) method. The comparative analysis was conducted using Leximancer. Findings illustrate a complex growing research corpus on mindfulness that is somewhat confused. The results from the scoping review show three shared domains in mindfulness classifications: short-term effects of mindfulness, long-term effects of mindfulness, and mindfulness practices. The results from the content mapping show four domains of mindfulness research: mental health, behavioural change, cognitive neuroscience, and ethical mindfulness. Operational definitions of mindfulness are not articulated clearly in these domains. Conceptual and operational definitions in the ‘ethical mindfulness’ domain are not yet developed. To enhance scientific progress in mindfulness research, further investigations of mindfulness classifications need to be developed. Content mapping and semantic typology is a potential candidate for future classification. More attention should be paid to developing operational definitions according to specific research domains. Scholars in the ethical mindfulness domain will need solid conceptual and operational definitions to support their research efforts.

## Introduction

Historically embedded in Buddhist traditions, mindfulness is gaining a growing interest in both healthcare settings and mainstream practices. In healthcare settings, mindfulness emerges as a treatment and an intervention, not only for patients but for healthcare workers [1]. Mindfulness is considered a tool to prevent and treat disease, increase the ability to cope with pain and chronic illness, and reduce stress [2]. In the United Kingdom, the National Health Service (NHS) recommends mindfulness-based cognitive therapy (MBCT) for depression [3]. For healthcare workers, mindfulness is featured in healthcare training to reduce stress, foster compassion, and reduce medical errors. Particularly, 79% of medical schools in the United States offer mindfulness training [4]. Outside of healthcare, mindfulness is practised in schools, prisons, corporations, sports teams, and through mobile apps. Mindfulness has become a billion-dollar capitalised industry [5]. As of May 2020, more than 20,000 books featured mindfulness on Amazon, promoting the benefits of mindful sleeping, mindful teaching, mindful painting, mindful leadership, and a mindful nation, to name a few [6]. The most popular mindfulness app Headspace has 31 million users worldwide [7].

Yet, many forms of mindfulness are used in healthcare settings and mainstream practices without being standardised or scientifically validated across different disciplinary contexts [8]. The increasing popularity of mindfulness calls for high-quality scientific research to provide policy guidance to regulate and standardise its use. However, it is challenging to conduct, validate, and communicate scientific mindfulness research, because mindfulness is a multifaceted concept that has not yet been theoretically contextualised across contributing disciplines (e.g. [9]). Mindfulness is being practised and researched in a wide array of discipline areas from neuroscience and positive psychology to behavioural change and management [10]. The rapid expansion of mindfulness without scientific context setting results in ambiguities and inconsistencies around the boundaries of the theoretical and practice domain.

As mindfulness has become an umbrella term, the lack of clarity in domains and definitions as well as the proliferation of mindfulness theories and practices are questioned on the ethicality of their methods [11, 12]. In particular, contemporary mindfulness-based interventions (MBIs) are often criticised for incorporating traditional but ‘non-scientific’ elements of mindfulness such as “compassion” and “wisdom” [13]. Yet such elements provide impacts in healthcare contexts such as fostering compassion in healthcare workers [14]. Such widely divergent viewpoints undermine the potential for mindfulness interventions to be effectively evaluated. Consequently, the application of an intervention that is seen as only anecdotally effective may be stymied because it lacks scientific precision.

This research provides a semantic map to enable understanding of mindfulness in different research and practice domains and contexts. This will ameliorate intensional vagueness and therefore permit greater transparency in inter/trans/cross-disciplinary research endeavours. This paper is the first of its kind to attempt to include the entirety of the domain. This builds on Brumett [15] clarifications of the metaphysical and epistemic domains of mindfulness theory. By identifying the domains and the definition of mindfulness in each domain, the study supports health policymakers to (1) set a cornerstone to regulate mindfulness practices in healthcare settings, (2) identify whether mindfulness is a treatment or a therapy (or both) in healthcare contexts, and (3) set criteria for scientific assessments of mindfulness practices. This study helps researchers in healthcare research to choose the most appropriate definition and measurement for their research. The study will provide a guide for researchers to limit and inform the scope of studies across a range of disciplines. It will also provide opportunities for theories and practices to be validated and communicated across domains. Finally, this research promotes interdisciplinary researchers to embed their scientific contributions in their target research domain(s).

This study uses a comprehensive scoping review of classifications of mindfulness and a semantic concept mapping of mindfulness studies. The scoping review followed the preferred reporting items for scoping reviews and meta-analyses (PRISMA) protocol, searching relevant terms in mindfulness studies from January 2015 to March 2021. The specific search terms are contained in S1 Appendix.

The paper first discusses the importance of shared meanings within the context of the study. It then presents the methodology of the study and outlines the procedures used to develop the mindfulness’ research domains and the shared definitions. The results and discussion section provide an overview of the classifications and shared definitions of mindfulness across research domains and assesses the alignment between the classifications and the content mapping. Finally, the paper presents conclusions and implications for future research.

## Definitions in mindfulness research

In scientific research, the ability to replicate and validate measures is directly related to the ability for a particular concept to be theoretically stable across research contexts, especially when it comes to measuring abstract human constructs (see for example, [16, 17]). The ability to measure a phenomenon is premised on the ability to describe, in concrete terms, an idea or concept sufficiently that another researcher seeking to evaluate the concept can produce the same outcomes [18, 19]. Thus, measurements must be valid and reliable, and the theoretical meaning should be stable within the discipline (domain) and potentially transferable across relevant disciplines and contexts. The creation of theoretical meaning is problematic because humans create their own worlds and distinguish among concepts in their own ways within their own contexts [20]. In natural language systems, where language evolves and measurement and validation are not usually required, discrepancies between meanings are often inconsequential, if only because meaning is created as a result of dialogic conversations [21]. However, in research, especially in health and medicine, the meaning of a term has to be both precise and reliable so that interventions can be assessed for efficacy based on evidence [22, 23]. The meaning of a term is contextualised and human-centric [20]. A term means different things in different situations and to different people within their own contexts (e.g. social, cultural, political, geographic, and disciplinary contexts) [24]. Without context, there can be multiple meanings for one term. For example, the word ‘run’ has approximately 171 definitions [25]. In corporate environments, ‘run’ means ‘managing a project’, while in athletics ‘run’ is perceived as a sport. To understand a term, it is therefore important to identify the contexts that are relevant to the term, the relationships of that term to other terms within each context, and how each aspect of the meaning of the term is constructed in each context.

The term ‘mindfulness’ has at least four definitions and descriptions in the Oxford English Dictionary, the Cambridge English Dictionary, and the Macmillan Dictionary. Definitions vary from a “quality of being conscious”, a “mental state”, to a “practice of being aware of body, mind, and feelings” or a “technique involving focusing on the present moment” [25, 26]. These definitions and descriptions taken from dictionaries provide an overview of mindfulness without being embedded within a specific theoretical or disciplinary domain. Dictionary definitions are a natural language starting point for knowledge development *across* domains. Researchers *within* the domain will be aware of the scope of definitions within their own domain, they may not be aware of others’ perspectives. Mindfulness can therefore mean thinking (cognitive), feeling (affective), or doing (behaviour, conative)–different things, at different times, depending on the authors’ disciplinary background. We assert that for evidence-based research, within the same research domain, mindfulness cannot be all cognition, affection, or behaviour at once and be meaningfully deployed as a theory (e.g. [27–30]). The potential for inconsistencies in an emerging evidence base starts with imprecision in terminology.

### Meaning in scientific research

In scientific research, meaning is constructed based on two levels of definition: the conceptual and the operational [31]. The conceptual definition defines a concept (term, idea, or ‘thing’) in terms of abstract characteristics and the concepts’ relations to other conceptual entities [32]. The conceptual definition is used to explain how a concept is constructed, related to, and connected with other things within specific contexts (i.e. the scientific definition is bounded within a discipline or context) [31]. For example, the conceptual definition of ‘anxiety’ is a feeling of uneasiness and worry. The operational definition defines a concept by linking the term to the concrete world and guides researchers in measuring that concept. The operational definition is usually called a ‘construct’ because it is a constructed idea [33]. It is used to identify a set of procedures (i.e. operations, activities) applied to determine the concrete nature of a concept and its properties and to measure it [34]. To ‘operationalise’ a concept is to provide a replicable series of steps to measure it. The nature of a concept and its properties are called dimensions; an operational definition can have many dimensions (multi-dimensional) or only one (uni-dimensional) [32]. For example, the operational definition of ‘anxiety’ is an emotion characterised by an unpleasant state of turmoil, often accompanied by nervous behaviour. ‘Unpleasant state of turmoil’ and ‘nervous behaviour’ are two dimensions of anxiety and can be measured by the Hamilton Anxiety Rating Scale [35].

### Problems with conceptual definitions of mindfulness

The conceptual definition of mindfulness is complex, inconsistent, and not precisely used, as we will show in this paper. Currently there is no scientifically operationalised definition of mindfulness that has been acceptable to all the disciplines that conduct mindfulness research. Thus, there exists no gold standard of reference that can be used to communicate and validate mindfulness theories or practices [36]. An impactful definition in healthcare contexts (with over 4000 citations in Google Scholar as at July 2020) is provided by [37] in the Mindfulness-based stress reduction (MBSR) program. In MBSR, mindfulness is defined as “the non-judgemental acceptance and investigation of present experience, including body sensations, internal mental states, thoughts, emotions, impulses and memories, in order to reduce suffering or distress and to increase well-being” [38 p4]. Yet Kabat-Zinn framed this definition as a practical instruction rather than a conceptually precise measurement tool [39]. The non-evaluative and non-judgemental nature of the MBSR definition is not an adequate theoretical description of canonical mindfulness (for example). However, non-judgmentalism is useful as a practical instruction and represents a “skilful means” to mindfulness [40 p7]. Mindfulness, in this conceptualisation, is a means to an end–the end in this case being a state of being that is more aware of itself [41]. In this school of thought, mindfulness is conceptualised as an act or a behaviour that leads to affective and cognitive outcomes. In contrast, as clarified by Kabat-Zinn [42], mindfulness is the awareness emerging through attention on purpose, which is the result and therefore an ‘end’ instead of a ‘means’. This apparently contradictory proposition causes confusion on whether mindfulness is being studied as a treatment or an outcome. As a result, it leads to the inconsistency in measuring outcomes in practice.

Additionally, the construction of conceptual definitions of mindfulness is inconsistent across fields. Mindfulness is variously defined in relation to the capacities of attention, awareness, memory/retention, or acceptance/discernment, nonjudgment, and compassion [9]. These mindfulness terms, such as ‘acceptance’, ‘nonjudgment’, and ‘compassion’, are also discussed and studied from different perspectives (e.g. the study of Buddhism, positive psychology, or management) [43]. For instance, Bodhi [44] explicitly states that acceptance is not part of mindfulness, as acceptance does not distinguish between wholesome (non-attachment, non-agression, calmness, faith, energy) and unwholesome (delusion, laziness, lack of faith, gloominess) states of mind. In this dilemma the argument is thus: in order to be mindful, one must also be wholesome, thereby negating mindfulness as a behaviour leading to affective and cognitive outcomes by asserting it as comprising all states or acts; past and future. This dilemma also conflates temporal aspects of antecedent and consequence. For the effects of mindfulness to be measured, it is first necessary to determine if it is an antecedent to or a consequence of therapy, treatment, or intervention [45]. Mindfulness may also be a trait, a state, a practice, or a set of religious ideals. However, again, it cannot be all these at once and be precisely measured within the scope of a single study, embedded within a specific discipline (e.g. [27–30]). It is, however, possible for studies to operationalise mindfulness differently and measure precisely *within* disciplinary domains. The conundrum occurs when the measures are applied across domains without consideration for context.

The argument regarding the ‘manipulation’ of mindfulness, especially in healthcare practice settings, is often due to vague definitions [39, 46, 47]. In particular, some healthcare practices and fitness programs are marketed as mindfulness but there are no regulations or standards to validate the claim [9]. For instance, mindfulness mobile apps like Headspace are criticised for stripping away the values of mindfulness by overlooking external causes of suffering, such as political, economic, or social factors [11]. The lack of a suitable operational definition of mindfulness limits the capacity to refute claims made in relation to mindfulness therapies, leading to claims that the practice is ineffective, when it may merely be poorly operationalised.

Additionally, some Buddhist commentators are critical of mainstream mindfulness that is marketed as equivalent to Buddhist practice, while being ungrounded in the traditional reflective morality of Buddhist ethics [44]. These critics use the term ‘McMindfulness’ to question the use of mindfulness as a marketing image of an idealised lifestyle [11]. Such statements are reflective of an ideological stance that spirituality or religiosity is a necessary antecedent to mindfulness. However, this is in contrast to programs such as MBSR, which is used in many healthcare contexts, that are overtly secular in their approaches to mindfulness. Initially, MBSR was designed to enhance mental well-being, but it does not address longer-term outcomes of mindfulness practice that provide impacts on daily life or spiritual traditions [48].

### Problems with extant operational definitions of mindfulness

For an operational definition of mindfulness, there are several measures available, all with different dimensions and different definitions. For example, there is the Mindfulness Attention Awareness Scale [49], the Five Facet Mindfulness Questionnaire [50], the Toronto Mindfulness Scale [51] and the Daily Spiritual Experience Scale [52], amongst others. This indicates that there is no shared agreement between mindfulness scholars as to the conceptual or operational definition of mindfulness. Although some operational definitions are useful across certain disciplines, such as the Mindful Attention Awareness Scale (MAAS), the inconsistent use of operational definitions, when applied across domains without thought for the exigencies of discipline and context, leads to challenges in the measurement and study of mindfulness.

The inconsistent use of conceptual definitions results in the inconsistent application of operational definitions, which causes unreliable measurements across mindfulness studies [9]. The complexity and confusion that surrounds operational definitions of mindfulness limits the potential for a theory or a practice to be informed and validated. Current measurements mostly use self-report questionnaires [9]. As a result, these studies are questioned for reliability, especially where the terms in the questionnaires are ambiguous or have contested meanings, such as ‘awareness’ and ‘nonjudgement’ [10]. These terms may have specific meanings within disciplinary contexts, but these do not necessarily translate into other contexts.

Furthermore, mindfulness researchers are still debating on whether mindfulness is one-dimensional or multidimensional [53], and whether mindfulness is a trait or a state [54]. The Mindfulness Attention Awareness Scale (MAAS), as the most widely disseminated measurement of mindfulness, is one-dimensional [55]. However, other measurements are multidimensional, such as the Five Facets Mindfulness Questionnaire (FFMQ) [50] or the Kentucky Inventory of Mindfulness Skills (KIMS) [56]. Disagreement regarding the dimensions of mindfulness creates confusion regarding which dimensions should be included or excluded in a particular healthcare context. In medicine, the random controlled trial (RCT) remains the gold standard for evidence [22, 57] and valid experiments start with precise instruments [58]. As a result of confusion over measurements of mindfulness, it becomes impossible to create and replicate appropriate experiments that assess the efficacy of mindfulness interventions, regardless of their potential benefit, because the measurement instrument is imprecise.

### Context and research domains

For definitions to be useful for establishing shared meaning across disciplines, there needs to be agreement about what the term means within a specific context [20]. In a conversation, the meaning of a term may be understood by the context in which the conversation takes place [59]. For example, if we use the term ‘aggressive’ in relation to someone’s behaviour in the United States, this can be seen to be a positive statement (e.g. the USA connotations of the word are confident, emphatic, demanding, strong-willed, sure of themselves). If the same statement is made in Australia, the term could be perceived as an insult (e.g. the Australian connotations of the word are overbearing, dogmatic, domineering, combative). Similar to context in a conversation, the research domain contextualises a definition by identifying the shared frames of reference and alignment between studies. In addition, naming a research domain will usually indicate theoretical and morphological boundaries, as well as prescribe both the conceptual and operational definitions of the term to ensure that meaning amongst scholars in the field is consistent. Thus, the research domain enables the establishment of semantic validity, which is ensuring the meaning of a concept to be precisely and consistently transferable from one person to another person [60]. The research domain, therefore, guides researchers to use precise and consistent definitions and measurements that match the research domain [61]. By making a definition consistently transferable (i.e. by using formal disciplinary-framed language), the research domain also allows theories and practices to be communicated and validated by experts and researchers within the same domain.

However, it is when the terms are transferred across contexts that semantic clarity is most necessary. It is in these cases that informal and different meanings can be fraught with potential for error. For example, in computer science a ‘mouse’ is a completely different object to a ‘mouse’ in the biological sciences. When communicating within the discipline there is often no need for explanation. It is when communicating out of the discipline that conceptual and operational definitions are required. The corpus of knowledge regarding mindfulness has grown rapidly in multiple divergent disciplines and neither conceptual nor operational definitions have been agreed.

### The problem of mindfulness research domains

The rapid expansion of mindfulness has resulted in a diverse range of indefinite research domains across disciplines. However, alongside the lack of an agreed scientific definition, there is not yet an agreement as to which specific disciplines comprise mindfulness research. The multifaced meaning of mindfulness raises conflicts between academic disciplines over how mindfulness is defined, measured, and popularly presented. For example, while mindfulness has its historical origin in Buddhism, it was first introduced formally to the Western scientific healthcare by Kabat-Zinn [37] under the mindfulness-based stress reduction (MBSR) program. The application of mindfulness has in time departed from its spiritual origins and has spread across the mainstream of medicine, healthcare, education, government and criminology [62]. As mentioned previously, Buddhist scholars have criticised the presentation of mindfulness in recent Western psychology publications [11]. These modern understandings depart from the accounts of mindfulness in early Buddhist texts and authoritative commentaries in the Theravada and Indian Mahayana traditions [44]. Valerio [63 p3] introduces the idea that conflict between academic disciplines potentially indicates “a personal, institutional, or paradigmatic battle for ownership” over mindfulness. The present study aims to clarify the conceptual and theoretical parameters of mindfulness research with a view to fostering coherence by helping to understand disciplinary boundaries and contexts of use.

As there is currently no agreement on research domain nor shared conceptual and operational definitions, mindfulness research and practices in healthcare settings often use imprecise definitions that do not serve the purpose of the replicable medical scientific research or practice. Therefore, mindfulness research and practices are challenging to validate and are not reliable in different contexts. This research seeks to clarify extant mindfulness research by reviewing the previous classifications of mindfulness to identify research domains and the definitions used in each domain. The classified research domains are then compared to a content-mapping of contemporary mindfulness research to assess the alignment between the classifications and the actual constructions of mindfulness research.

## Methodology

This study comprises two stages: a scoping review of the classifications of mindfulness (Stage 1) and concept mapping for comparison (Stage 2). Stage 1 aims to identify the classifications of mindfulness from different authors within the different research domains. Stage 2 assesses whether the classifications identified from Stage 1 align with the constructions of contemporary mindfulness research. The section below presents in detail how each stage was conducted.

### Stage 1—Scoping review

**Fig 1:**
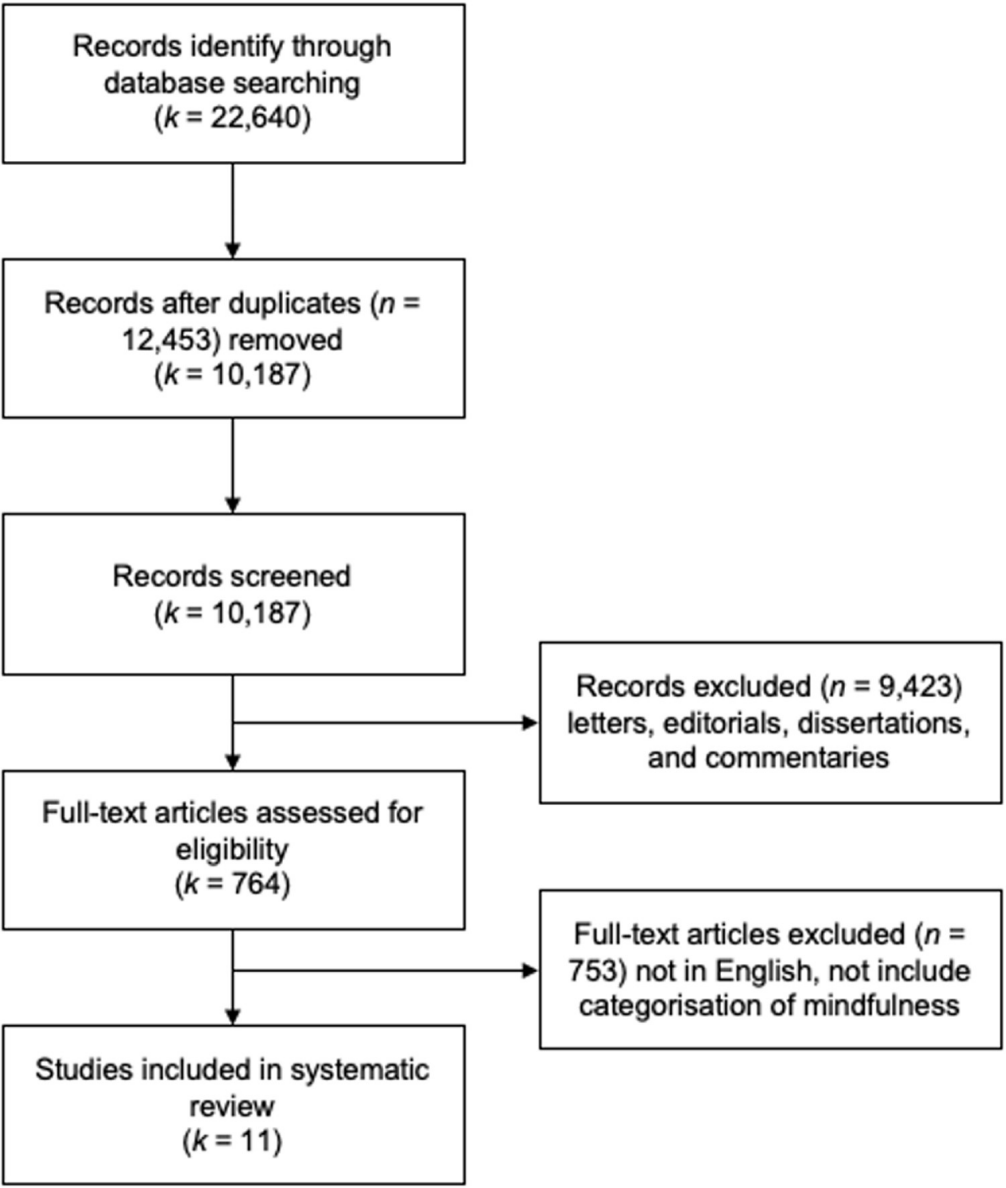
Flow of studies to scoping review.

A scoping review is a review method aimed at synthesizing research evidence and mapping the existing literature in a certain field of interest [64, 65]. Unlike systematic reviews, scoping review methods are considered of particular use when the topic has not been extensively reviewed or is of a complex heterogeneous nature [64]. Scoping review is chosen for this study because scoping review is useful for the purposes of clarifying key concepts/definitions in the literature and of identifying key characteristics of factors related to a concept [64, 65]. Our scoping review followed the protocol for scoping review developed by Tricco, Lillie [65] and then improved by Joanna Briggs Institute (JBI). Our study design, implementation, analysis, and reporting followed the preferred reporting items for scoping reviews and meta-analysis (PRISMA) protocol, detailed in Fig 1. After data was collected, we performed a thematic analysis to identify the key classification themes, the classified mindfulness domains, and identified conceptual and operational definitions of mindfulness. The below section describes in detail our data collection process.

#### Sample selection

*Search methods*. This study developed and pilot-tested a comprehensive search strategy, using relevant search terms for all data sources from January 2015 to October 2019, as shown in Table 1. The data sources were in English language only.

**Table 1:** Database searched in Stage 1 and Stage 2.

Data source	Database
Electronic searches	SCOPUS, ProQuest, ERIC, MEDLINE, PsycInfo, PubPsych, Cochrane Central Register of Controlled Trials (CENTRAL), PsycInfo, PubPsych
General search engines	Google Scholar, Intute, Turning Research into Practice (TRIP)
Science citation index	Science Citation Index, Social Science Citation Index
Hand searches	Key journals–Springer, SAGE, Elsevier
Reference lists	Snowballing was performed to screen the references of identified literature for potentially relevant studies

#### Search strategy

This study used the ‘population, intervention, compare, and outcome’ (PICO) search tool to organise the search framework and to list terms according with the main concepts of the research question. PICO was chosen because it helps researchers cover a comprehensive range of literature [66]. In this scoping review, as the research purpose did not require comparison, we did not include the ‘compare’ element and focused instead on three remaining elements (population, intervention, and outcome). The search strategy is outlined in Table 2.

**Table 2:** Stage 1 search strategy.

Population	Mindfulness
Intervention/indicator	Comparison, scoping review, seta-analysis, citation analysis, classification, categorisation
Outcome	Domain, definition, theory

#### Screening process

A comprehensive screening process following PRISMA guidelines was conducted, detailed in Fig 1. Studies were reviewed by two researchers independently in three stages, based on the exclusion and inclusion criteria. Duplicated items were removed before the first stage. During the first stage of title screening, titles of studies identified from the search were assessed for inclusion. If both authors rejected a title, it was excluded from the review. Titles approved by either researcher would be moved to abstract screening. If both researchers rejected a study at abstract screening, it also would be excluded from the review. In the third stage, full texts of abstracts selected in the previous stage were screened for eligibility. Only those studies approved by both researchers were included in the review. In the event of any disagreements, a third researcher would arbitrate, and a consensus was reached. The rationale for exclusion was provided for all studies which were excluded through this process. A final list of articles was prepared for data extraction.

The search acquired 22,751 articles. Amongst that number, 765 were eligible for further review and 12 articles met the inclusion criteria for analysis (see inclusion criteria below).

*Types of studies*. This review considered both qualitative and quantitative studies. For quantitative studies, researchers considered experiment designs including randomised controlled trials, non-randomised controlled trials, quasi-experimental studies, and before and after studies. For qualitative studies, researchers considered designs such as phenomenology, grounded theory, ethnography, action research, and feminist research. This review also included conceptual papers and scoping review papers, because the main purpose was not to evaluate the impact of mindfulness but to assess how mindfulness is defined and classified. Therefore, any scientific arguments and discussions about mindfulness were taken into account.

*Inclusion criteria*. Published studies, conceptual papers, and scoping reviews conducted on the classification of mindfulness were eligible for inclusion. Studies were examined if they had been published in peer-reviewed journals between January 2015 to October 2019.

Studies that met the following criteria were included:

- Full-text, original research in a peer-reviewed journal
- Published in English language
- Examine mindfulness’ classifications, definitions, models, and/or measurement criteria

*Exclusion criteria*. Studies were excluded if they were editorials, commentaries, and dissertations, or they did not include the classification of mindfulness research.

*Review methods*. Studies were reviewed in regard to their classification methods, the identified mindfulness domains, and the definition of mindfulness in each domain. Studies with similar classification methods were grouped to identify the shared classification method. We then compared the results of classification methods, their applications, and their limitations. Finally, we identified the shared mindfulness domains across all classifications, which is potentially the main domains of mindfulness research.

### Stage 2—Concept mapping for comparison

In Stage 2, we used textual data-mining software Leximancer to examine mindfulness research articles and build a conceptual mapping of mindfulness research to compare to the results from Stage 1. Leximancer was chosen for this research because it assisted the researchers to identify research domains by analysing text conceptually (concept analysis) and relationally (relational analysis). Leximancer allowed the data to be examined for strength and the extent of relationships between concepts, ensuring that key semantic connections were not missed [67]. Leximancer has been used in previous studies to assess the composition of research domains and to trace change over time and compare clusters of research [68].

In the conceptual analysis, the Leximancer algorithm analysed the texts to identify concepts, their relationships to each other, and their frequency [69]. Concepts in Leximancer are collections of words that generally occur together. Words in a concept are weighted accordingly to how frequently they occur in sentences containing that concept, compared to how frequently those words occur elsewhere [70]. Concepts are presented as dots in a visual concept map. The concept dots are also connected by path lines, illustrating the extent of the conceptual distance between a core concept and a related concept [70].

In the relational analysis, Leximancer measured the relationships between identified concepts. Leximancer automatically identified key concepts and themes through its automated procedure for thesaurus construction [70]. Themes are a collection of concepts that are closely linked together and which frequently occur together [70]. Themes are presented in a concept map, with the colour of each theme indicating the level of thematic relevance. The most prominent themes are depicted in red (hot) down to those of least importance in purple (cold). In this research, Leximancer’s themes guided the researcher to identify the content clusters that formed research domains in mindfulness research.

#### Sample selection

*Search methods*. As Stage 2 was designed as a comparison with Stage 1’s results, Stage 2 used the same search database (shown in Table 1) and the same search timeframe (from January 2015 to October 2019) as Stage 1.

*Search strategy*. We developed a search strategy (shown in Table 3) with “mindfulness” and its popular practices–“meditation”, “zen”, and “yoga”–as intervention, and included outcomes of “attention”, “awareness”, “behaviour”, “compassion”, “psychology”, “behaviour”, “sustainability”, and “social change”.

**Table 3:** Stage 2’s search strategy.

Intervention	Outcome
Mindfulness	Psychology, compassion, attention, awareness, behaviour, sustainability, social change, intervention
Meditation	Psychology, compassion, attention, awareness, behaviour, sustainability, social change, intervention
Zen	Psychology, compassion, attention, awareness, behaviour, sustainability, social change, intervention
Yoga	Psychology, compassion, attention, awareness, behaviour, sustainability, social change, intervention

#### Screening process

**Fig 2:**
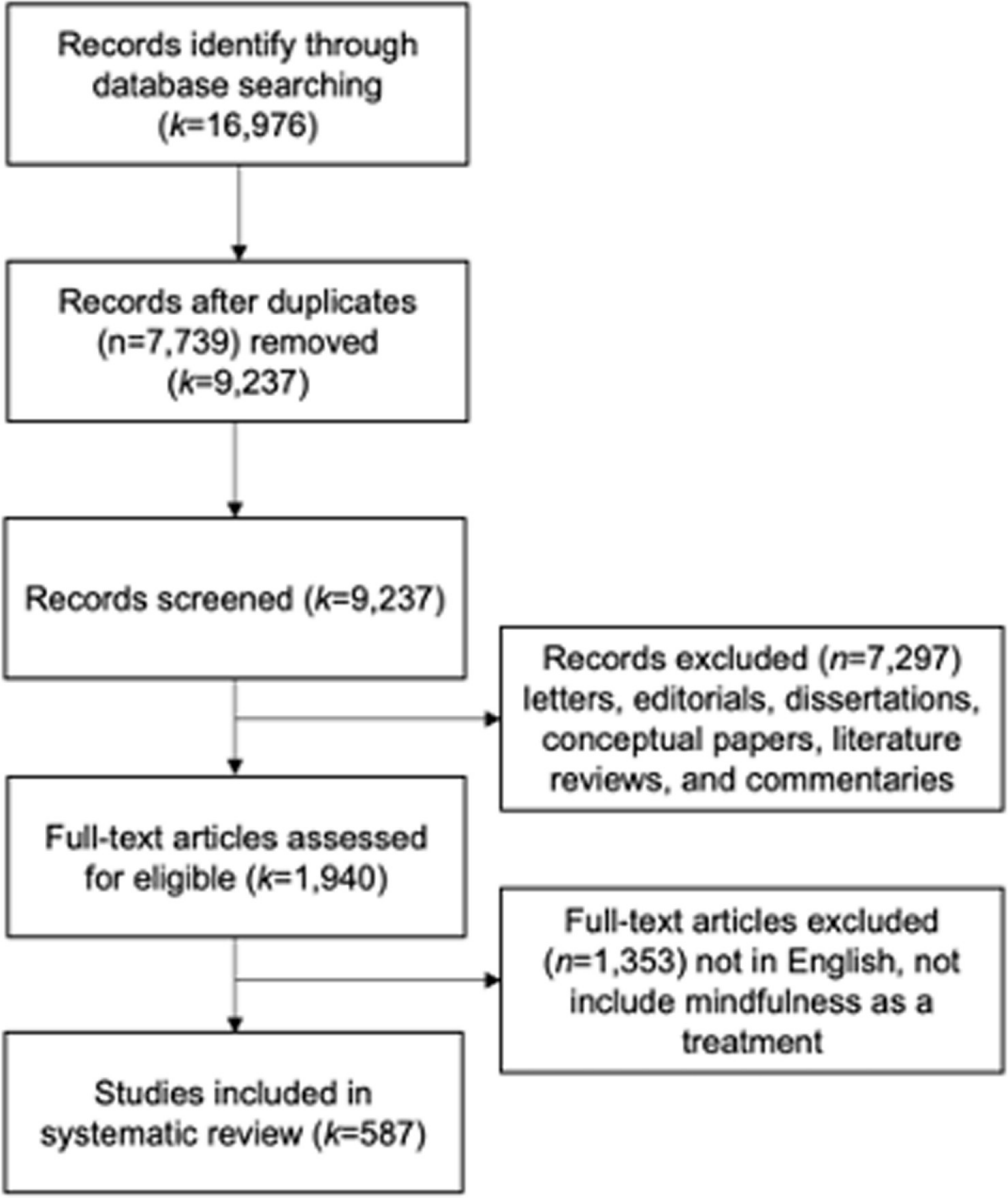
Flow of studies to concept mapping.

The search for Stage 2 was also conducted in accordance with PRISMA guidelines and with the same screening process as Stage 1, as shown in Fig 2. However, in Stage 2, the focus was on the studies of mindfulness in different contexts. Therefore, the types of studies, inclusion, and exclusion criteria were different to Stage 1, as shown in the following sections. The search acquired 36,978 articles. Amongst that number, 1,965 were deemed eligible for further review and 552 articles met the inclusion criteria for analysis.

*Types of studies*. As the focus of Stage 2 was on the studies of mindfulness in different contexts, Stage 2 includes a broader range of research than Stage 1 and included only empirical intervention studies. Stage 2 considered both qualitative and quantitative studies. For quantitative studies, this review considered experimental studies including randomised controlled trials, non-randomised controlled trials, quasi-experimental studies, and before and after studies. For qualitative studies, this review considered studies that used research methods such as phenomenology, grounded theory, ethnography, action research, and feminist research.

*Inclusion criteria*. Published studies, conceptual papers, and scoping reviews conducted on mindfulness were eligible for inclusion. All studies included were published in peer-reviewed journals between January 2015 to March 2021.

Studies included in Stage 2 met the following criteria:

- Full-text, original research, published in a peer-reviewed journal
- Published in English language and human studies
- Studied mindfulness, including both quantitative and qualitative methods

*Exclusion criteria*. Studies were excluded if they were editorials, commentaries, or dissertations.

## Results and discussion

### Stage 1—Scoping review of classifications of mindfulness meanings

This section presents contemporary classifications of mindfulness in research between 2015 and 2021. Table 4 shows the classification method used to categorise mindfulness research, the identified mindfulness concepts, and the properties of each conceptual term. Studies with similar classification methods were grouped to identify a shared classification method. We identified three classification methods: occurrence-based, mechanism-based, and historical development and context-based. Occurrence-based classification methods classify mindfulness research based on how mindfulness is cultivated, whether it is through intention practice or intentionally triggered in everyday life. Mechanism-based classification methods classify mindfulness based on the effects of mindfulness on cognition or on the brain. Historical development and context-based classification methods classify mindfulness studies based on traditions of mindfulness.

**Table 4:** Existing perspectives on shared properties of mindfulness.

Author	Classification method	Concept	Properties in definition of concept
Kabat-Zinn [71]	Occurrence	Deliberate mindfulness	Moment-to-moment, non-judgmental awareness, intentionally cultivated by paying attention in a specific way i.e. in the present moment, and as non-reactively, non-judgmentally, and openheartedly as possible [71]
		Effortless mindfulness	Moment-to-moment, non-judgmental awareness, spontaneously cultivated by paying attention in a specific way i.e. in the present moment, and as non-reactively, as non-judgmentally, and as openheartedly as possible [71]
Davidson and Kaszniak [72]	Occurrence	State of mind and brain (Long/short-term practitioners)	State of mind and brain produced by meditation practices [73]
		Brain and traits effects (Long-term practitioners)	Long-term effects of mindfulness practices on brains, traits, and daily life [74]
		Procedure	Set of instructions provided to participants [75]
Harrington and Dunne [76]	Historical development and context	Zen	A radically anti-authoritarian practice and philosophy that was concerned with the transformative effects of experiencing the world as it really was [77]
		Transcendental meditation	Specific form of silent, mantra meditation, a quick-and-easy form of meditation [78]
		Mindfulness-based stress reduction (MBSR)	Melding of different traditions: Zen, the ‘nondual’ Mahamudra tradition of meditative practice, various yogic traditions, and a modernist version of insight meditation [71]
Tang, Hölzel [79]	Mechanism	Attention control	Meditation traditions emphasise the necessity to cultivate attention regulation early in the practice [79]
		Emotion regulation	Mindfulness-based emotion regulation may involve a mix of attentional deployment, cognitive change, and response modulation [80]
		Self-awareness	Dis-identification from self-concept results in the freedom to experience a more genuine way of being [81]
Kirmayer [82]	Historical development and context	Mindfulness in Buddhism	A tool to achieve ethical and spiritual goals of eliminating greed, hatred, and delusion while cultivating wisdom, compassion, and loving kindness [83]
		Mindfulness as therapeutic modality	Present-centred, non-judgmental or non-evaluative attention [71]
Nilsson and Kazemi [47]	Occurrence	Attention and awareness	Awareness refers to the ability to be deeply self-aware and monitor cognitions, emotions, and bodily sensations as responses to the environmental influence [47]
		Present-centred	Present-centeredness refers to being in the moment or engaging in the being-mode [47]
		External events	An umbrella term for occurrences, objects, and stimuli in the environment (i.e. happenings outside of the body) and should as such be understood as the outer milieus’ impact on the mind-body functioning [47]
		Cultivations	Refers to fostering or developing one’s character through mindfulness [47]
		Ethical-mindedness	The social dimension of mindfulness and can be used as a socio-political tool with the potential to contribute to justice, peace, and ecological balance in the world [47]
Khoury, Knäuper [84]	Historical development and context	Buddhist tradition	The development of lucid awareness of what is occurring in the present moment [44]
		Western mindfulness-meditation	Mindfulness as awareness itself, a form of innate capacity that is virtually transparent [71]
		Langerian	Mindfulness as the creation of new categories, openness to new information, and awareness of more than one perspectives [85]
Chiesa, Fazia [62]	Mechanism	Therapeutic/clinical effect	Evaluation of clinical efficacy as well as the psychological and/or neuropsychological effects of the interventions under examination [62]
		Non-therapeutic/clinical effect	Neuroimaging of functional and structural brain changes, analysis of the mechanism underlying the therapeutic effects and biological correlates [62]
		Type of intervention	Five sub-groups: Mindfulness-based interventions, MBSR, mindfulness-based cognitive therapy, mixed interventions, and vipassana [62]
Creswell [10]	Historical development and context	MBSR and related group-based mindfulness intervention	MBSR has stimulated the development of many mindfulness interventions that share the same basic program structure but are modified to treat specific populations or outcomes [10]
		Mindfulness intervention retreats and brief interventions	Mindfulness meditation residential retreat programs, ranging from three days to three months, are a powerful way to deliver intensive and well-controlled doses of mindfulness intervention [10]
		Internet and smartphone application mindfulness interventions	Internet- and smartphone-based mindfulness programs [10]
		Mindfulness-related interventions	Interventions that incorporate mindfulness training exercises as one component of a broader treatment program [10]
Schindler, Pfattheicher [86]	Mechanism	Self-regulated attention	Attention on present-moment experiences [87]
		Opening, accepting, non-judgemental orientation	Meta‐cognitive awareness of one’s internal mental experiences, observing them as such, and watching them come and go [87]
Kudesia [88]	Mechanism	Mindful attention	Emphasises the early perceptual end of the metacognitive level. Originated in clinical settings for people with mental distress [49]
		Mindful conceptualising	Emphasises the later conceptual end of the linear sequence [89]. This definition originated in the study of repetitive situations.
		Mindful metacognition	A metacognitive process, one that informs how people adjust their information processing to their current situation [88]
Kee, Li [90]	Historical development and context	Condition/Issue	The applicability of mindfulness as a possible approach for a host of mental and physical ailments [90]
		Construct/Philosophy	The nature of mindfulness as a human experience [90]
		Modality	The approaches that mindfulness is applied in practice [90]
		Population/Setting	Intervention that is applied for broader population that embedded in daily life [90]
		Research Methodology	The research methodologies used in mindfulness research [90]

#### Classification methods

The results of Table 4 show that there is no general agreement about the conceptual or operational definitions of mindfulness (Column 4), nor how studies should be classified (Column 2). Four studies classified mindfulness research by the regulation of mindfulness at different cognitive levels (i.e. a mechanism-based approach). Three studies classified mindfulness research by the frequency and intentionality involved in mindfulness practice (occurrence-based approach). The rest of the studies (four out of 11) classify mindfulness research by its context (context-based approach), based on either the historical development or the practice traditions of mindfulness. The next section describes these three approaches.

*Mechanism-based approach*. Mechanism-based studies classify mindfulness research by how mindfulness is regulated at different cognitive levels [79, 88]. For instance, Kudesia [88] classifies mindfulness studies into three domains according to the effects of mindfulness on three cognitive levels. The three domains classified by Kudesia [88] are attention (mindfulness regulates attention over time), conceptualisation (mindfulness refines established concepts), and metacognitive (mindfulness adjusts information processing to a situation). In Tang, Hölzel [79] classification, mindfulness research is classified according to the process of self-regulation, including attention control, emotion regulation, and self-awareness. Mechanism classifications provide a baseline to investigate the underlying effects of mindfulness. However, mechanism-based classifications do not consider studies in other areas such as behavioural change, Buddhist studies, management, or education. Therefore, mechanism-based classifications may hamper interdisciplinary research and limit mindfulness’ application to only the psychology and neuroscience fields.

*Occurrence-based approach*. Occurrence-based studies classify research concerning the intentionality and frequency of mindfulness for long-term and short-term practitioners [71, 72]. For example, Davidson and Kaszniak [72] classify mindfulness studies by the effects of mindfulness on short-term and long-term practitioners. The term ‘mindfulness’ is often applied to clinical interventions (intermittent clinician-led practice), with the term ‘meditation’ being applied to more sustained practitioner-led performances. Thus, the occurrence (clinical/personal) of mindfulness is an important element in these descriptions, as is the purpose and intention of the practice. Nonetheless, the terms ‘mindfulness’ and ‘meditation’ are used somewhat interchangeably, and self-reported effects from these practices cannot be reliably extrapolated into measures of effectiveness, given that occurrence is likely to lead to different outcomes.

*Context-based approach*. Context-based classifications focus on the trajectory of the mindfulness field. Particularly, context-based classifications categorise studies according to the origin of mindfulness practices used in the study. Buddhist/Hindu mindfulness, Langerian mindfulness (the mindfulness tradition introduced by Langer and Newman [91], and modern mindfulness are three main domains identified by context-based classifications. Within each main domain, some sub-domains are identified. For instance, [76] divide the Buddhist/Hindu domain into two other sub-domains, Zen and transcendental mindfulness. Context-based classifications emphasise different translations of mindfulness from the Buddhist/Hindu text and the development of mindfulness from these terms. These themes of research are focussed on the philosophical and religious or spiritual aspects of mindfulness. Consequently, the focus is not so much on interventions or measurable outcomes associated with mindfulness practice, as it is the context of mindfulness or meditation practice and applications of these practices to life or health. Context-based approaches are therefore contradistinctive to mechanism-based approaches, although there are substantive overlaps with occurrence-based approaches.

#### Shared domains of all classification approaches

The previous analysis has shown that the current domains are conceptually discrete with some similarities. Accordingly, we grouped them into shared domains or constructs where there was enough difference between them to necessitate a separate category, for the purposes of providing a logical structure for our research endeavours. While they are not mutually exclusive, these domains can provide some clarity as to how our research contributes to a specific field of scholarship. The three shared domains that covered mindfulness studies comprehensively is shown in Table 5. These identified domains are then compared with the Leximancer analysis in Stage 2 to see whether the current classification is matched with the real composition of mindfulness research. Table 5 shows the shared domains, the relevant research areas, and conceptual definitions or descriptions of mindfulness used in each shared domain. Table 5 also illustrates the substantive differences between descriptions.

**Table 5:** Shared and unshared concepts across domains.

Shared domains (construct)	Relevant research areas (disciplines)	Conceptual definitions/descriptions
Short-term effects of mindfulness	Psychology, Neuroscience, Clinical settings, Stress reduction, Mental illness, Therapeutic applications	Paying attention in a particular way: deliberately, in the present moment, and non-judgmentally [92]
		Awareness of present-moment experience, with intention and purpose, without grasping on to judgments [93]
Long-term effects of mindfulness	Behavioural change, Wellbeing (health) management, Education, Neuroscience, Psychology	Bringing one’s complete awareness to the present experience on a moment-to-moment basis [94]
		Mindfulness is a process of regulating attention in order to bring a quality of non-elaborative awareness to current experiences and a quality of relating to one’s experience within an orientation of curiosity, experiential openness, and acceptance [87]
		Mindfulness involves intentionally bringing one’s attention to the internal and external experiences occurring in the present moment [50]
		Mindfulness as awareness itself, a form of innate capacity that is virtually transparent [39]
Mindfulness perspectives	Langerian mindfulness	Mindfulness as the creation of new categories, openness to new information, and awareness of more than one perspective [95]
	Buddhist mindfulness	Theravada mindfulness [96], Zen, Mahayana mindfulness [97]
	Modern mindfulness	Mindfulness-based stress reduction (MBSR) [71], and mindfulness-based cognitive therapy [98]

The *short-term effects of mindfulness* domain concerns the effects of mindfulness on a certain state of mind. The short-term mindfulness domain includes conceptual objects related to transient (short-term) effects that are outcomes of mindfulness practices. These are further elucidated in the following section. These effects are studied in psychology, neuroscience, and therapeutic fields [71]. Practices in this domain are mostly in clinical settings and are intentional, i.e. people participate in interventions with intent [88]. The descriptions share the following elements: purpose or intent, present moment, and non-judgement. However, there is divergence on other elements, such as awareness and attention, which are conceptually and practically different objects.

The *long-term effects of mindfulness* domain concerns the effects of mindfulness on traits and behaviours (self-awareness, conceptualising, metacognitive). The long-term effects of mindfulness domain incorporates the outcomes of mindfulness practice, such as the ability to focus on present experiences, open mindedness, and awareness of self. The application of research in long-term domain covers a broader research area including behavioural change, positive psychology, subjective well-being, and education [84]. This domain also includes elements such as awareness and attention, as well as intentionality and purpose, but is premised on the idea that these skills can be taught, leading to enhanced experiences.

The *mindfulness perspectives* domain concerns the study of mindfulness practices, their origin, and their process in use for research purpose. The mindfulness perspectives domain does not describe or define mindfulness practice, so much as it examines the impact of various mindfulness cultural traditions and practices. Most research in this domain concerns whether a certain mindfulness practice is practised or researched according to an established scientific or religious process. The main debate within the mindfulness perspectives domain is whether contemporary mindfulness practices are stripping away the ethical values of the original Buddhist/Hindu practices [96]. Consequently, the debate in this arena is one of epistemology, axiology, and the moral worthiness of what is and is not ‘mindfulness’ from different perspectives (i.e. orthodoxy). In this context, axiology comprises both ethical and aesthetic value.

#### Conceptual definitions of mindfulness

The conceptual definitions of mindfulness varied across domains. Within the short-term effects of mindfulness domain and the long-term effects of mindfulness domain, conceptual definitions have shared properties in their own domain and are distinctive to definitions in the other domain. On the other hand, the mindfulness perspectives domain had the most confusion and debate [96]. Considering the often-religious foundations of this domain, it is not surprising that such debates about orthodoxy are prevalent; after all, such debates exist wherever there is a battle for ‘rightness’. However, such debates are best entered into by the genuinely informed and are possibly not required pre-reading for the intervention designer in a clinical health setting.

*Short-term effects of mindfulness*. In the short-term effects of mindfulness domain, mindfulness is defined as a state of mind that is intentionally cultivated by a mindfulness practice that occurs both in short-term and long-term practitioners [71]. The definitions in the short-term effects of mindfulness domain are mostly developed from the earlier conceptual definition of Kabat-Zinn [37]. In this domain, mindfulness is replete with descriptions of the meditative process and its possible mechanisms of action [15]. Those descriptions shed mindfulness’ practices of Buddhist truth claims, appealing to the universality of introspection and attentional control [15]. Although that definition serves the purpose of clinical settings, it is often criticised for stripping away the ethical values of mindfulness and ignoring the benefits of mindfulness to the ‘bigger-than-self world’ [9, 99]. However, overlooking possible spiritual commitments embedded in the mindfulness literature could also lead to confusing terms in empirical studies [15]. While these definitions are applicable to other domains, the focus of research in this domain is on clinical settings and actively seeks to understand the effects or outcomes of mindfulness as an intervention. The term ‘meditation’ is not widely used in this setting, partially because the underlying tradition has used ‘mindfulness’ since its introduction into clinical settings.

*Long-term effects of mindfulness*. In the long-term effects of mindfulness domain, mindfulness’ conceptual definitions are often named ‘dispositional mindfulness’ or ‘trait mindfulness’. The names suggest the effects of mindfulness on the deeper cognitive level. Mindfulness in the long-term effects of mindfulness domain is defined as an innate capacity of awareness to the present experience that is spontaneously cultivated by paying attention in a specific way [71]. The definitions of mindfulness in the long-term effects of mindfulness domain are widely accepted in the fields of wellbeing, management, and behaviour change [100–104]. Yet, the definitions themselves have raised confusion over their own wordings; “present experience” and “spontaneously cultivated”, for example, are broad and hard to measure [9 p39]. Due to the confusing wording in definitions, ‘trait mindfulness’ is often mishandled in practice [99]. Therefore, a clarification of terms within trait-mindfulness can help to narrow the scope of mindfulness in this domain. A narrower scope would facilitate understanding of whether a pre-disposition towards mindfulness (a trait) is necessary in order for an intervention to be effective, or, alternatively, if a trait of mindfulness practice can be built over time. Knowing the difference would expedite intervention design and provide insight into how long interventions should be applied in order to be efficacious in the longer-term.

*Mindfulness perspectives*. The mindfulness perspectives domain concerns different practices of mindfulness and contains the most debates about the conceptual definition of mindfulness. Within this domain there are three sub-domains: Langerian mindfulness, Buddhist mindfulness, and modern mindfulness. Langerian mindfulness is considerably separated from the other two because its definition is distinctive [95]. Langer and Weinman [105] conceptualisation of mindfulness is built on the cognitive information-processing framework. The Langerian mindfulness definition includes components of novelty seeking, engagement, novelty producing, and flexibility. The components of Langerian mindfulness, as a sociocognitive ability, are different, if not opposed, to that of Buddhist/Hindu mindfulness and modern mindfulness, which can be a state of mind, a trait, or a process. Although the Buddhist/Hindu mindfulness and modern mindfulness sub-domains have some shared components in their definitions, there is no agreement between these two fields. Buddhist/Hindu mindfulness emphasises Buddha’s teaching of mindfulness, which is defined as “right effort and right concentration that connects the energetic application of the mind to its stilling and unification” [44 p20]. Practices that fit into the Buddhist/Hindu sub-domains are Zen, Theravada Buddhism, and transcendental mindfulness. The Buddhist/Hindu mindfulness definition is later translated into modern mindfulness, which emphasises bare attention to the moment-to-moment experience, such as that which is used in the MBSR program [71]. However, scholars from the Buddhism field criticise the translation to “bare attention”, stating there are many ways to achieve mindfulness and some of those ways do not require “bare attention” [44 p27]. Buddhist studies also suggest that certain elements of mindfulness, such as compassion, should be included in mindfulness definitions [106].

#### Operational definitions of mindfulness

Each of the eleven studies discuss the conceptual definitions of mindfulness, but only one of them discusses an operational definition of mindfulness. Khoury, Knäuper [84] is the only paper that posits an operational definition across domains. However, their classification does not analyse or identify in detail different operational definitions in one domain, nor does it specify when to use a particular operational definition. For example, in the modern mindfulness domain, they only mention some practices in programs, such as MBSR and MBCT, without clarifying whether they are multi-dimensional or uni-dimensional, or whether there may be different worldviews at work.

Stage 1 illustrates that there are many possible interpretations of the conceptual boundaries and operational constructions associated with mindfulness interventions. Consequently, basic research which relies on precision is limited. In order to ascertain what disciplinary purviews are at work in the mindfulness domain and to evaluate their conceptual coverage, a further analysis using Leximancer was undertaken.

### Stage 2—Comparison to Leximancer result

Stage 2 compares Stage 1 results to the results from a Leximancer content analysis. In this component of the study, 552 articles were uploaded into Leximancer for analysis (see S2 Appendix for a list of articles). Common terms and words like ‘study’, ‘research’, ‘table’, ‘sample’, and ‘people’ were removed. Given that Leximancer also treats variations of words as different terms, the Leximancer term-book was used to merge closely related and plural word versions, such as ‘change’ and ‘changes’. However, words that may appear to be closely related, such as ‘behaviour’ and ‘behavioural’, were not combined, as these words can appear in conceptually different spaces. For example, the term ‘behaviour’ most often refers to human actions, while ‘behavioural’ is most often associated with cognitive behaviour. The Leximancer conceptual map and Leximancer cloud were produced iteratively until stable results are created [107].

**Fig 3:**
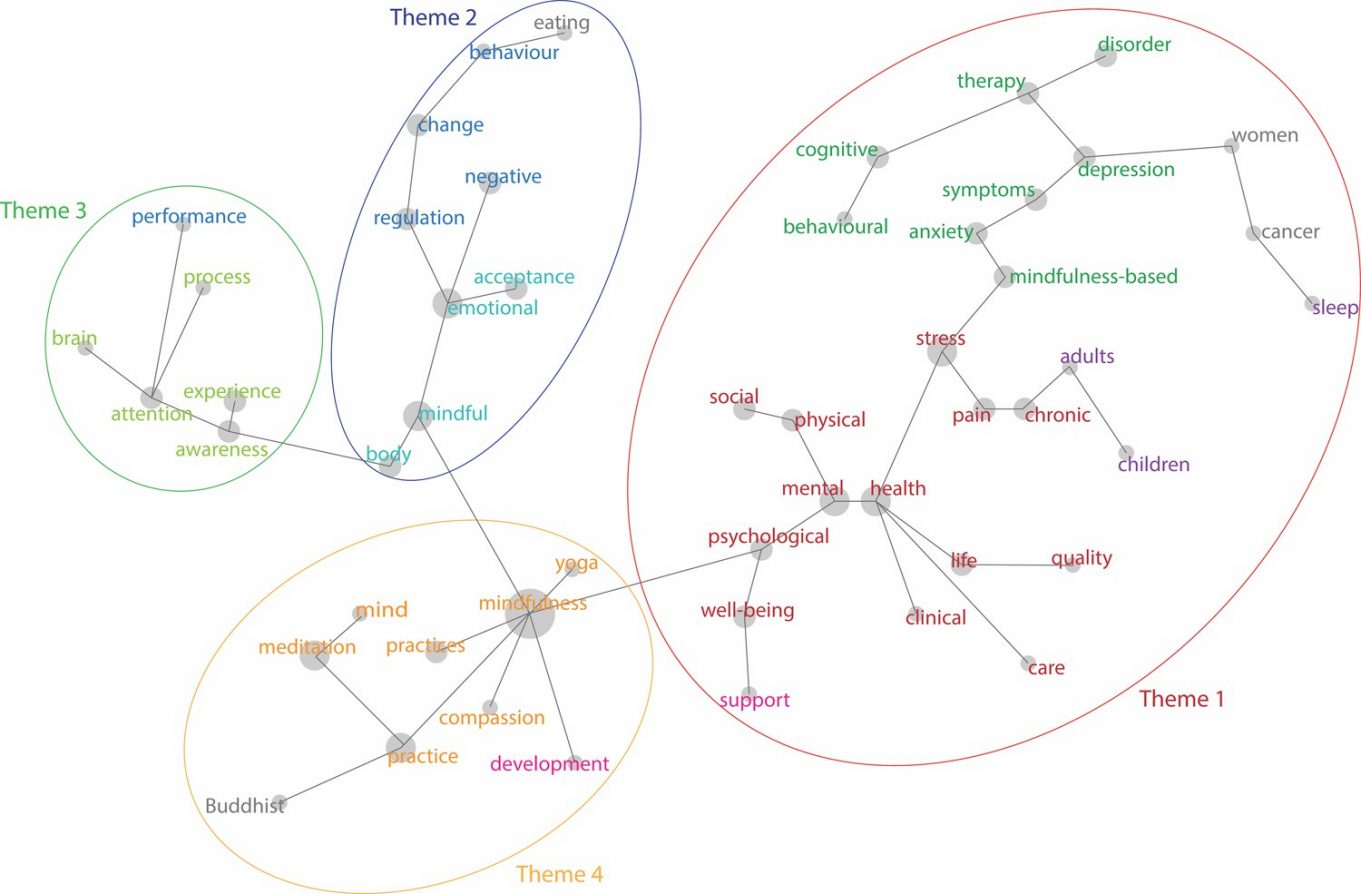
Mindfulness concept map.

Fig 3 presents the concept map exported directly from Leximancer’s text mining. The result shows that there are total 31 key concepts within mindfulness research, presented as dots in the map (Fig 3) and listed in Table 6. The results show there are four themes identified by a central term. In the four themes, the most dominant theme has the main concept coloured in red (indicating a ‘hot’ or close relationship between terms) and the least dominant theme has the concept coloured in blue (‘cold’). The colour indicates the distance between the key concept and the central term. The key term is also determined by prevalence. For example, in the map, the word ‘mindfulness’ is not used as often as the term ‘mental health’, and so does not appear as red, despite being the central concept of this study. The map also shows the conceptual pathways between terms associated with each other. For example, Theme 1 (red outline) is the central (hot, most prevalent) theme. While Theme 2 (blue outline), 3 (green outline), and 4 (yellow outline) are more distantly linked via the term ‘mindfulness’, which is most prevalent in Theme 4, but creates the pathway from Theme 1 to Themes 2 and 3.

**Table 6:** Main themes and key terms.

	Theme	Key terms	Key authors
1	Mental health and well-being (MHWB)	Mental health, physical health, psychological well-being, stress, mindfulness-based, anxiety, depression, therapy, cognitive behavioural, disorder, pain, chronic, sleep	Mantzios [108]; Mantzios, Egan [109]; Lomas, Medina [110]; Lomas, Medina [111]; Kabat-Zinn [71]; Goldberg [112]
2	Behaviour change (BC)	Mindful, emotional acceptance, emotional regulation, behaviour change	Singh, Lancioni [113]; Singh, Lancioni [114]; Singh, Lancioni [115]
3	Cognitive neuroscience (CNS)	Awareness, experience, attention, brain, attention process, performance	Tang, Hölzel [79]; Kudesia [88]; Mooneyham, Mrazek [116]
4	Ethical mindfulness (EM)	Meditation, compassion, yoga, mindfulness practices, mind, mindfulness development, Buddhist	Shonin and Van Gordon [117]; Shonin, Van Gordon [118]; Van Dam, van Vugt [119]; Van Dam, van Vugt [9]

***Theme 1*** is denoted *mental health and well-being* (MHWB) and comprises the concepts ‘mental health’, ‘physical health’, ‘psychological well-being’, ‘stress’, ‘mindfulness-based’, ‘anxiety’, ‘depression’, ‘therapy’, ‘cognitive behavioural’, ‘disorder’, ‘pain’, ‘chronic’, and ‘sleep’. Coloured in red, this theme is the most dominant. In other words, the current focus of mindfulness research is on this theme. As the concepts of this theme include mental health, physical health, psychological well-being, and mental disorders, we conclude that the main focus of research in this theme is on mental health and psychological wellbeing.
***Theme 2*** is denoted *behaviour change* (BC), and comprises the concepts ‘mindful’, ‘emotional acceptance’, ‘emotional regulation’, and ‘behaviour change’. Coloured in blue, this theme is of the least focus of mindfulness research. In other words, more research needs to be invested in this theme. As the concepts in this theme are behaviour change and emotional regulation, we conclude that the main focus of research in this theme is on the effects of mindfulness on changing behaviour and regulating emotion.
***Theme 3*** is denoted *cognitive neuroscience* (CNS), and comprises the concepts ‘awareness’, ‘attention’, ‘brain’, ‘process’, and ‘performance’. As the concepts in this theme have a cognitive neuroscience focus, we conclude that this theme concerns the impacts of mindfulness on the brain and the cognitive function of the brain.
***Theme 4*** is denoted *ethical mindfulness* (EM), and comprises the concepts ‘yoga’, ‘practices’, ‘compassion’, ‘meditation’, and ‘Buddhist’. This theme concerns mindfulness practices and traditions. As the concepts in this theme are different practices and the different origins and core values of mindfulness, we conclude that this theme concerns the ethical elements of mindfulness.

From this analysis, we propose that contemporary mindfulness research is divided into four distinct domains: (1) mental health/well-being, (2) behaviour change, (3) neuroscience/cognitive, and (4) ethical mindfulness. These four clusters are identified in accordance with the above four themes from the analysis. These are outlined in the following section.

#### Theme 1: Mental health and well-being

Research in the mental health and well-being (MHWB) domain mainly addresses the use of mindfulness as a treatment for mental disorders, including anxiety, stress, and sleep disorders. However, it is different to Stage 1’s short-term effects of mindfulness domain, because the MHWB domain does not include cognitive neuroscience research that addresses the effects of mindfulness on the brain and how mindfulness regulates attention and awareness. Furthermore, it is also distinct from Stage 1’s long-term effects of mindfulness domain, because the cluster does not address behaviour change, nor does it comprise studies into how mindfulness regulates emotion, awareness, or attention. Mindfulness-based interventions, including studies from Goldberg [112]; Kabat-Zinn [120]; Lomas, Medina [110]; Mantzios, Egan [109]; Pagnini and Langer [121], fit into the mental health/well-being theme. Mindfulness in this theme is researched as a means to resolve medical and medically problematised conditions.

#### Theme 2: Behaviour change

The main focus of research in Theme 2 concerns the influence of mindfulness on fostering long-term behaviour change (BC) and emotional regulation. Different to Stage 1’s long-term effects of mindfulness domain, research in the BC domains does not include research about long-term effects of mindfulness at neural or cognitive levels. Studies concerning changing behaviour for prosocial/environmental concerns (e.g. [5, 122]), behavioural therapy (e.g. [114, 123–125]), and emotional control (e.g. [126–129]) appear in this theme.

#### Theme 3: Cognitive neuroscience

Theme 3 includes research that is almost exclusively bounded by the physical effects of mindfulness on the brain and its functions. Research in the cognitive neuroscience (CNS) theme concerns the regulation of mindfulness at these cognitive levels and mindfulness’ effects on the brain. This theme includes research that studies both the long-term and short-term effects of mindfulness at neural and cognitive levels [116, 130–135]. It is the most medicalised of the themes, with both physicians and psychologists examining the impacts of mindfulness on an array of processes such as healthy aging (e.g. [136, 137]), brain structures (e.g. [134]), and high risk mental states (e.g. [138]).

#### Theme 4: Ethical mindfulness

Research in the ethical mindfulness theme concerns the fundamental values and practices of mindfulness (e.g. [9, 117, 118]). Different to Stage 1’s mindfulness perspectives domain, the ethical mindfulness theme does not include research on mindfulness-based interventions or Langerian mindfulness. The key terms in this theme link mindfulness to Buddhist traditions but also to compassion and meditation, as well as yoga, mindfulness practice, and the development of mindfulness. Consequently, this theme is not as replete with intellectual ‘turf wars’ over the orthodoxy of the approach.

As a result of these differences, mindfulness is a long way from being a universally measurable construct. However, within clusters and across related clusters, there is potential for more precise operational definitions. For example, the MHWB theme centres on terms such as mental and physical health, and these are not as widely used in the EM theme, despite being contiguous on the map. The overlapping domains cause confusion around which definition and measurement should be used in a certain context. Fig 3 and Table 6 illustrate that there are different contexts of use when it comes to mindfulness research. The four contexts or themes identified here can be used to set theoretic boundaries around research to ensure operationalised research is accurate, precise, and able to be replicated within the domain.

The four identified themes also offer insights into how researchers have been exploring mindfulness and therefore may be indicators of nascent disciplinary offshoots. For example, while health and well-being might be closely related, the map shows that mental health is problematised differently, with divergent paths towards disorder on the one hand (via anxiety and depression) and psychological well-being and quality of life on the other. The latter sub-group appears to be more about examining support and care options than it is about interventions and therapies.

These four themes also present a practical approach that could diminish the extant overlap and duplication between domains, providing opportunities for theoretical clarity in research methods and approaches. The clarity of the identified clusters offers opportunities to cross-fertilise ideas between domains in future research, allowing for increased understanding of mindfulness theories and practices from multiple perspectives. For instance, the ethical mindfulness cluster is distinct from the cognitive neuroscience cluster. Therefore, mindfulness definitions in these two clusters can be different because they exist in different theoretical contexts. On the other hand, the neuroscience cluster and behaviour change cluster share a strong connection, which suggests shared elements in the definitions of these two clusters could be further developed for interdisciplinary projects or intervention programs.

## Conclusion and implications

### Implications for mindfulness research

This research provides a semantic map to further understanding of mindfulness conceptualisations in different theoretical and practical domains and contexts. Extant mindfulness research is currently using inconsistent definitions and measurements that do not serve the purpose of the research [9]. As a result, such research cannot measure the relevant elements and dimensions of mindfulness where measurement is required, such as in clinical settings. For example, mindfulness research for lifestyle illnesses such as diabetes or obesity is currently measured according to the short-term effects of mindfulness, while the longer-term effects would be a preferable approach for lifestyle-related research [139].

This study illustrates that previous mindfulness classifications are either too narrow or too broad for meaningful operationalisation. Previous classifications either focus only on one aspect of mindfulness or include too many themes in one domain. For example, some classifications focus on cognitive levels and neglect the behavioural and ethical elements of mindfulness [79, 88]. In contrast, there are classifications that attempt to combine all the modern mindfulness practices into one domain [140, 141]. The resultant overlaps prohibit precise and meaningful measurement because there are too many elements being considered.

Research in the long-term mindfulness domain uses both ‘trait mindfulness’ and ‘state mindfulness’ as the conceptual definition in their research, although both terms have multiple definitions [142–144]. On the other hand, some definitions are used in all domains of mindfulness research, but they do not always include all relevant elements of descriptions. In particular, MBSR’s definition of mindfulness is the most widely used definition. Yet this definition is more a description of practice and does not cover out-of-the-setting or daily and ethical mindfulness practice [119]. For effective measurement, researchers must move away from relying on broad and umbrella terms of mindfulness that are potentially not fit for purpose and move toward more explicit definitions for their specific healthcare contexts.

This study also calls for further development of operational definitions and measurements of mindfulness within identified themes. While there are conceptual definitions and some strong descriptions in each of the themes, most operational definitions are underdeveloped and therefore contribute to unproductive debates about validity when the key issue is imprecision.

We found only one study [140] that (briefly) discussed mindfulness’ operational definition across domains. Yet, in each domain, without precise and consistent operational definitions, there is no agreed measurement to conduct, communicate, or validate mindfulness research, or to cooperate with others conducting mindfulness research. For example, because ethical mindfulness does not have any agreed conceptual and operational definitions, there is no scientific measurement which can measure whether a practice, theory, or research approach is ethical. Operational definitions adopted from other fields might be unattractive to ethical mindfulness researchers, as it would constrain the boundaries of their intellectual endeavour. However, a definition that can be used within this context might be helpful to establish shared meanings with other researchers. Operational definitions exist in a domain of practice within which the research is embedded. By not trying to be all things to all people, the current tensions might be resolved and opportunities for growing interdisciplinary understandings may emerge. Particularly, clusters with a shared strong connection have the potential for cooperation without working at cross-purposes when defining parameters. The clarity of themes and shared elements in definitions allow the development of working definitions in interdisciplinary research. For example, the mental health cluster and the behaviour change cluster share a strong connection, which suggests shared elements in their definition could be developed for interdisciplinary research.

This research also shows that the current approach of using or looking for an umbrella, one-size-fits-all definitions is not necessary. Some clusters are fundamentally different and require different properties in definitions of mindfulness. Specifically, cognitive neuroscience is concerned with the mechanism of how mindfulness changes the brain and not with the ethical elements of mindfulness. As a result, each cluster can use one distinct definition that serves the purpose of the research of the cluster and does not attempt to comply with the purpose of other clusters. Future research does not require looking for and using a one-fits-all definition. Rather, an explicit definition for each cluster would enable intradisciplinary research and delineate intellectual boundaries.

This research also shows that ethical mindfulness is a separate domain that is underdeveloped. The ethical mindfulness cluster, which is concerned with Buddhist mindfulness, does not connect to the health-related research and appears under-developed in the text-mining map. This disconnection also implies that ethical mindfulness includes core values that are different to other clusters [36]. Currently there is no mindfulness scale dedicated to ethical mindfulness or spiritual values.

### Implications for healthcare practices

The identified themes provide a guideline for health policy to regulate and standardise mindfulness practice and measurement in different medical contexts. Three out of four themes (MHWB, CNS, and BC) are concerned with the use of mindfulness in healthcare practices. In different clusters, mindfulness provides different health benefits. For example, research in the MHWB theme provides recommendations for mental illnesses or stress management. In contrast, the BC theme provides recommendations for illnesses related to lifestyle such as diabetes or obesity [145] and where long term behaviour change is the objective of the intervention.

Although mindfulness is popular in mainstream practice, the understanding of mindfulness as a whole is still problematic. This study shows that there are distinctly different themes of research being conducted. Different practices and definitions bring about different outcomes and benefits. Understanding the different contexts of mindfulness will facilitate more effectual research design across the range of disciplinary situations.

### Limitations

Content mapping is a potential alternative method for classifying mindfulness research on a large scale. It would identify themes, but also the shared properties between mindfulness definitions. Thus, future research might use the text-mining results to identify the shared properties of mindfulness definitions in each cluster to provide a shared definition that can be universally used in each cluster. However, Leximancer is a content analysis tools that relies on access to data gathered by humans. It is possible that major clusters of research were missed in this process, although the search and scoping review process was as reliable as possible.

Another limitation is that the study did not include the entire range of available literature, such as grey literature (reports, government documents, and evaluations). Additionally, the literature covered only contemporary discussions of mindfulness, excluding any studies conducted before 2015. Therefore, the research neglected earlier discussions regarding mindfulness, especially during the booming period from 2011 to 2014 [120]. Furthermore, search terms included only mindfulness and popular mindfulness practices (meditation, zen, and yoga). Additionally, this study did not include more niche religious terms such as ‘transcendental mindfulness’ or emergent practices such as ‘mindful eating’ or ‘mindful walking’. This is potentially an explanation for the weak connection between themes within the ethical mindfulness cluster. Future research might expand the search in terms of timeframe, the range of literature, and include more practices to collect a larger range of literature for more accurate classifications.

In conclusion, this research provides a new way to look at mindfulness research and its intellectual domains. Poor classification of research domains complicates mindfulness research, as it becomes impossible to make a contribution within any disciplines where there is no shared meaning or where there are no boundaries to the knowledge building process. However, our research shows that mindfulness research is not as widely scattered as the debate appears to indicate. Clustering into theoretically meaningful groups is possible and these groups are sufficiently distinct from each other that separate definitions are practicable for the purposes of conducting research. Separate definitions will enable different viewpoints to be accommodated and still facilitate scientific research progress, without the need for debate on which definition is the ‘correct’ one. In this case, there are multiple correct ways of making progress and building the evidence base. The identified clusters also provide a blueprint for mindfulness researchers to develop a conceptual definition and an operational definition that is explicit for each cluster. Such clarity in definitions fosters communication and validation of new theories and practices in each cluster. Such clarification also sets boundaries between domains and promotes more precise, explicit, and effectively designed intervention programs as a treatment for specific healthcare needs. It is not the purpose of this study to construct new and improved operational definitions of mindfulness, as this is best undertaken by those who are deeply informed as to the purposes, connotations and intentional properties of such theoretical spaces. However, we do argue that such work is necessary within the disciplines identified here. A one size fits all strategy is unlikely to be acceptable.

## Supporting information

S1 Appendix Boolean search term. (DOCX)

S2 Appendix List of papers in the Leximancer analysis. (DOCX)

S1 File Glossary of terms. (DOCX)

S2 File Data availability statement. (DOCX)
