# Supplementary material for: The search for scientific meaning in mindfulness research: Insights from a scoping review
Source: PLoS One. 2022 May 4;17(5):e0264924. doi: 10.1371/journal.pone.0264924 (PMC9067662; doi:10.1371/journal.pone.0264924)
Supplement: S1 Appendix — (DOCX) [file pone.0264924.s001.docx]

# S1 Appendix – Boolean search term

| Stage | Population | Intervention | Outcome | Search term |
| --- | --- | --- | --- | --- |
| Stage 1 | Mindfulness | Comparison | Definition | Mindfulness AND comparison AND definition |
|  |  |  | Domain | Mindfulness AND comparison AND domain |
|  |  |  | Theory | Mindfulness AND comparison AND theory |
|  |  | Systematic Review | Definition | Mindfulness AND systematic review AND definition |
|  |  |  | Domain | Mindfulness AND systematic review AND domain |
|  |  |  | Theory | Mindfulness AND systematic review AND theory |
|  |  | Meta-analysis | Definition | Mindfulness AND meta-analysis AND definition |
|  |  |  | Domain | Mindfulness AND meta-analysis AND domain |
|  |  |  | Theory | Mindfulness AND meta-analysis AND theory |
|  |  | Citation analysis | Definition | Mindfulness AND citation analysis AND definition |
|  |  |  | Domain | Mindfulness AND citation analysis AND domain |
|  |  |  | Theory | Mindfulness AND citation analysis AND theory |
|  |  | Classification | Definition | Mindfulness AND classification AND definition |
|  |  |  | Domain | Mindfulness AND classification AND domain |
|  |  |  | Theory | Mindfulness AND classification AND theory |
|  |  | Categorisation | Definition | Mindfulness AND categorisation AND definition |
|  |  |  | Domain | Mindfulness AND categorisation AND domain |
|  |  |  | Theory | Mindfulness AND categorisation AND theory |
| Stage 2 | Mindfulness |  | Psychology | Mindfulness AND psychology |
|  |  |  | Compassion | Mindfulness AND compassion |
|  |  |  | Attention | Mindfulness AND attention |
|  |  |  | Awareness | Mindfulness AND awareness |
|  |  |  | Behaviour | Mindfulness AND behaviour |
|  |  |  | Sustainability | Mindfulness AND sustainability |
|  |  |  | Social change | Mindfulness AND social change |
|  |  |  | Intervention | Mindfulness AND intervention |
|  | Meditation |  | Psychology | Meditation AND psychology |
|  |  |  | Compassion | Meditation AND compassion |
|  |  |  | Attention | Meditation AND attention |
|  |  |  | Awareness | Meditation AND awareness |
|  |  |  | Behaviour | Meditation AND behaviour |
|  |  |  | Sustainability | Meditation AND sustainability |
|  |  |  | Social change | Meditation AND social change |
|  |  |  | Intervention | Meditation AND intervention |
|  | Zen |  | Psychology | Zen AND psychology |
|  |  |  | Compassion | Zen AND compassion |
|  |  |  | Attention | Zen AND attention |
|  |  |  | Awareness | Zen AND awareness |
|  |  |  | Behaviour | Zen AND behaviour |
|  |  |  | Sustainability | Zen AND sustainability |
|  |  |  | Social change | Zen AND social change |
|  |  |  | Intervention | Zen AND intervention |
|  | Yoga |  | Psychology | Yoga AND psychology |
|  |  |  | Compassion | Yoga AND compassion |
|  |  |  | Attention | Yoga AND attention |
|  |  |  | Awareness | Yoga AND awareness |
|  |  |  | Behaviour | Yoga AND behaviour |
|  |  |  | Sustainability | Yoga AND sustainability |
|  |  |  | Social change | Yoga AND social change |
|  |  |  | Intervention | Yoga AND intervention |
