# Supplementary material for: The search for scientific meaning in mindfulness research: Insights from a scoping review
Source: PLoS One. 2022 May 4;17(5):e0264924. doi: 10.1371/journal.pone.0264924 (PMC9067662; doi:10.1371/journal.pone.0264924)
Supplement: S2 Appendix — (DOCX) [file pone.0264924.s002.docx]

# S2 Appendix – List of papers in the Leximancer analysis

| Authors | Year | Title | Journal |
| --- | --- | --- | --- |
| Agarwal, Anulipi and Vidushi Dixit | 2017 | The role of meditation on mindful awareness and life satisfaction of adolescents | Journal of Psychosocial Research |
| Ahmadi, Sahel, Sara Ahmadi and Aida Kheirandish | 2016 | The Effectiveness of Yoga Practice on Positive Psychology Constructs: Meaning in Life, Gratitude and Marital Intimacy | International Journal of Psychology |
| Ahola, S Kohut, J Stinson, A Jelen and D Ruskin | 2019 | Feasibility and Acceptability of a Mindfulness-Based Group Intervention for Adolescents with Inflammatory Bowel Disease | Journal of clinical psychology in medical settings |
| Al, LI Daken and Muayyad M Ahmad | 2018 | The implementation of mindfulness-based interventions and educational interventions to support family caregivers of patients with cancer: A systematic review | Perspectives in psychiatric care |
| Albertson, Ellen R, Kristin D Neff and Karen E Dill-Shackleford | 2015 | Self-compassion and body dissatisfaction in women: A randomized controlled trial of a brief meditation intervention | Mindfulness |
| Alda, Marta, Marta Puebla-Guedea, Baltasar Rodero, Marcelo Demarzo, Jesus Montero-Marin, Miquel Roca and Javier Garcia-Campayo | 2016 | Zen meditation, length of telomeres, and the role of experiential avoidance and compassion | Mindfulness |
| Alsubaie, Modi, Rebecca Abbott, Barnaby Dunn, Chris Dickens, Tina Frieda Keil, William Henley and Willem Kuyken | 2017 | Mechanisms of action in mindfulness-based cognitive therapy (MBCT) and mindfulness-based stress reduction (MBSR) in people with physical and/or psychological conditions: a systematic review | Clinical Psychology Review |
| Amihai, Ido and Maria Kozhevnikov | 2015 | The influence of Buddhist meditation traditions on the autonomic system and attention | BioMed research international |
| Amutio-Kareaga, Alberto, Javier García-Campayo, Luis Delgado, Daniel Hermosilla and Cristina Martínez-Taboada | 2017 | Improving communication between physicians and their patients through mindfulness and compassion-based strategies: a narrative review | Journal of clinical medicine |
| Anālayo, Bhikkhu | 2019 | Meditation on the breath: mindfulness and focused attention | Mindfulness |
| Andreotti, Eva, Anne Congard, Sarah Le Vigouroux, Bruno Dauvier, Johan Illy, Rollon Poinsot and Pascal Antoine | 2018 | Rumination and Mindlessness Processes: Trajectories of Change in a 42-Day Mindfulness-Based Intervention | Journal of cognitive psychotherapy |
| Anheyer, Dennis, Matthew J Leach, Petra Klose, Gustav Dobos and Holger Cramer | 2019 | Mindfulness-based stress reduction for treating chronic headache: a systematic review and meta-analysis | Cephalalgia |
| Antoine, Pascal, Anne Congard, Eva Andreotti, Bruno Dauvier, Johan Illy and Rollon Poinsot | 2018 | A Mindfulness‐Based Intervention: Differential Effects on Affective and Processual Evolution | Applied Psychology: Health and Well‐Being |
| Auty, Katherine M, Aiden Cope and Alison Liebling | 2017 | A systematic review and meta-analysis of yoga and mindfulness meditation in prison: Effects on psychological well-being and behavioural functioning | International journal of offender therapy and comparative criminology |
| Aydogan, Hakan and Selma Demirtas Aydogan | 2016 | EEG attention and meditation responses of students on different presentation slide colors | Journal of Educational and Instructional Studies in the World |
| Azulay, J and T Mott | 2016 | Using mindfulness attention meditation (MAP) with a mixed brain injury population to enhance awareness and improve emotional regulation | J. Psychol. Clin. Psychiatry |
| Bach, Jennifer M and Tharina Guse | 2015 | The effect of contemplation and meditation on ‘great compassion’on the psychological well-being of adolescents | The Journal of Positive Psychology |
| Baer, Ruth, Catherine Crane, Edward Miller and Willem Kuyken | 2019 | Doing no harm in mindfulness-based programs: conceptual issues and empirical findings | Clinical psychology review |
| Baesler, E James | 2015 | Meditation in the classroom: Cultivating attention and insight | Listening Education |
| Bakosh, Laura S, Jutta M Tobias Mortlock, Dawn Querstret and Linda Morison | 2018 | Audio-guided mindfulness training in schools and its effect on academic attainment: Contributing to theory and practice | Learning and Instruction |
| Banerjee, Moitree, Kate Cavanagh and Clara Strauss | 2018 | Barriers to mindfulness: A path analytic model exploring the role of rumination and worry in predicting psychological and physical engagement in an online mindfulness-based intervention | Mindfulness |
| Bankard, Joseph | 2015 | Training emotion cultivates morality: How loving-kindness meditation hones compassion and increases prosocial behavior | Journal of religion and health |
| Barbaro, Nicole and Scott M Pickett | 2016 | Mindfully green: Examining the effect of connectedness to nature on the relationship between mindfulness and engagement in pro-environmental behavior | Personality and Individual Differences |
| Barkan, Tessa, Michael Hoerger, Autumn M Gallegos, Nicholas A Turiano, Paul R Duberstein and Jan A Moynihan | 2016 | Personality predicts utilization of mindfulness-based stress reduction during and post-intervention in a community sample of older adults | The journal of alternative and complementary medicine |
| Barnes, Nicholas, Patrick Hattan, David S Black and Zev Schuman-Olivier | 2017 | An examination of mindfulness-based programs in US medical schools | Mindfulness |
| Barnes, VA, JL Kristeller and MH Johnson | 2016 | Impact of mindfulness-based eating awareness on diet and exercise habits in adolescents | Int J Complement Alt Med |
| Barney, Jennifer L, Helen B Murray, Stephanie M Manasse, Cara Dochat and Adrienne S Juarascio | 2019 | Mechanisms and moderators in mindfulness‐and acceptance‐based treatments for binge eating spectrum disorders: A systematic review | European Eating Disorders Review |
| Barratt, Caroline | 2017 | Exploring how mindfulness and self-compassion can enhance compassionate care | Nursing Standard (2014+) |
| Bartlett, Larissa, Angela Martin, Amanda L Neil, Kate Memish, Petr Otahal, Michelle Kilpatrick and Kristy Sanderson | 2019 | A systematic review and meta-analysis of workplace mindfulness training randomized controlled trials | Journal of occupational health psychology |
| Baslet, Gaston, Barbara Dworetzky, David L Perez and Megan Oser | 2015 | Treatment of psychogenic nonepileptic seizures: updated review and findings from a mindfulness-based intervention case series | Clinical EEG and neuroscience |
| Basso, Julia C, Alexandra McHale, Victoria Ende, Douglas J Oberlin and Wendy A Suzuki | 2019 | Brief, daily meditation enhances attention, memory, mood, and emotional regulation in non-experienced meditators | Behavioural brain research |
| Bawa, Fathima L Marikar, Stewart W Mercer, Rachel J Atherton, Fiona Clague, Andrew Keen, Neil W Scott and Christine M Bond | 2015 | Does mindfulness improve outcomes in patients with chronic pain? Systematic review and meta-analysis | Br J Gen Pract |
| Beblo, Thomas, Sarah Pelster, Christine Schilling, Kristian Kleinke, Benjamin Iffland, Martin Driessen and Silvia Fernando | 2018 | Breath Versus Emotions: The Impact of Different Foci of Attention During Mindfulness Meditation on the Experience of Negative and Positive Emotions | Behavior therapy |
| Beccia, Ariel L, Corina Dunlap, Douglas A Hanes, Brendan J Courneene and Heather L Zwickey | 2018 | Mindfulness-based eating disorder prevention programs: A systematic review and meta-analysis | Mental Health & Prevention |
| Beck, Ann R, Heidi Verticchio, Scott Seeman, Emma Milliken and Heidi Schaab | 2017 | A mindfulness practice for communication sciences and disorders undergraduate and speech-language pathology graduate students: effects on stress, self-compassion, and perfectionism | American journal of speech-language pathology |
| Behan | 2020 | The benefits of meditation and mindfulness practices during times of crisis such as COVID-19 | Irish journal of psychological medicine |
| Bender, Stacy L, Rachel Roth, Alicia Zielenski, Zachary Longo and Ashley Chermak | 2018 | Prevalence of mindfulness literature and intervention in school psychology journals from 2006 to 2016 | Psychology in the Schools |
| Berger, Rony, Alaina Brenick and Ricardo Tarrasch | 2018 | Reducing Israeli-Jewish pupils’ outgroup prejudice with a mindfulness and compassion-based social-emotional program | Mindfulness |
| Beshai, Shadi, Lindi McAlpine, Katherine Weare and Willem Kuyken | 2016 | A non-randomised feasibility trial assessing the efficacy of a mindfulness-based intervention for teachers to reduce stress and improve well-being | Mindfulness |
| Biedermann, Britta, Peter De Lissa, Yatin Mahajan, Vince Polito, Nicolas Badcock, Michael H Connors, Lena Quinto, Linda Larsen and Genevieve McArthur | 2016 | Meditation and auditory attention: An ERP study of meditators and non-meditators | International Journal of Psychophysiology |
| Bilican, F Isil | 2016 | The Relationship Between Focused Attention Meditation Practice Habits, Psychological Symptoms, and Quality of Life | Journal of religion and health |
| Black, David S and George M Slavich | 2016 | Mindfulness meditation and the immune system: a systematic review of randomized controlled trials | Annals of the New York Academy of Sciences |
| Blake, Matthew J, Laura M Blake, Orli Schwartz, Monika Raniti, Joanna M Waloszek, Greg Murray, Julian G Simmons, Elizabeth Landau, Ronald E Dahl and Dana L McMakin | 2018 | Who benefits from adolescent sleep interventions? Moderators of treatment efficacy in a randomized controlled trial of a cognitive‐behavioral and mindfulness‐based group sleep intervention for at‐risk adolescents | Journal of Child Psychology and Psychiatry |
| Blake, Matthew J, Lian Snoep, Monika Raniti, Orli Schwartz, Joanna M Waloszek, Julian G Simmons, Greg Murray, Laura Blake, Elizabeth R Landau and Ronald E Dahl | 2017 | A cognitive-behavioral and mindfulness-based group sleep intervention improves behavior problems in at-risk adolescents by improving perceived sleep quality | Behaviour research and therapy |
| Blake, Matthew, Joanna M Waloszek, Orli Schwartz, Monika Raniti, Julian G Simmons, Laura Blake, Greg Murray, Ronald E Dahl, Richard Bootzin and Paul Dudgeon | 2016 | The SENSE study: Post intervention effects of a randomized controlled trial of a cognitive–behavioral and mindfulness-based group sleep improvement intervention among at-risk adolescents | Journal of Consulting and Clinical Psychology |
| Blake, Matthew, Orli Schwartz, Joanna M Waloszek, Monika Raniti, Julian G Simmons, Greg Murray, Laura Blake, Ronald E Dahl, Richard Bootzin and Dana L McMakin | 2017 | The SENSE Study: Treatment mechanisms of a cognitive behavioral and mindfulness-based group sleep improvement intervention for at-risk adolescents | Sleep |
| Blanck, Paul, Sarah Perleth, Thomas Heidenreich, Paula Kröger, Beate Ditzen, Hinrich Bents and Johannes Mander | 2018 | Effects of mindfulness exercises as stand-alone intervention on symptoms of anxiety and depression: Systematic review and meta-analysis | Behaviour research and therapy |
| Bluth, Karen and Tory A Eisenlohr-Moul | 2017 | Response to a mindful self-compassion intervention in teens: A within-person association of mindfulness, self-compassion, and emotional well-being outcomes | Journal of Adolescence |
| Bluth, Karen, Patricia NE Roberson and Susan A Gaylord | 2015 | A pilot study of a mindfulness intervention for adolescents and the potential role of self-compassion in reducing stress | Explore |
| Boden, Matthew Tyler, Jessica G Irons, Matthew T Feldner, Sarah Bujarski and Marcel O Bonn-Miller | 2015 | An investigation of relations among quality of life and individual facets of emotional awareness and mindfulness | Mindfulness |
| Borquist-Conlon, Debra S, Brandy R Maynard, Kristen Esposito Brendel and Anne SJ Farina | 2019 | Mindfulness-based interventions for youth with anxiety: A systematic review and meta-analysis | Research on Social Work Practice |
| Braden, B Blair, Teri B Pipe, Ryan Smith, Tyler K Glaspy, Brandon R Deatherage and Leslie C Baxter | 2016 | Brain and behavior changes associated with an abbreviated 4‐week mindfulness‐based stress reduction course in back pain patients | Brain and behavior |
| Breedvelt, Josefien, Yagmur Amanvermez, Mathias Harrer, Eirini Karyotaki, Simon Gilbody, Claudi LH Bockting, Pim Cuijpers and David Daniel Ebert | 2019 | The effects of meditation, yoga and mindfulness on depression, anxiety and stress in tertiary education students: A meta-analysis | Frontiers in psychiatry |
| Brem, Meagan J, Ryan C Shorey, Scott Anderson and Gregory L Stuart | 2019 | Exploring Gender Differences in the Relationship Between Dispositional Mindfulness and Compulsive Sexual Behavior Among Adults in Residential Substance Use Treatment | Mindfulness |
| Brito-Pons, Gonzalo, Daniel Campos and Ausiàs Cebolla | 2018 | Implicit or Explicit Compassion? Effects of Compassion Cultivation Training and Comparison with Mindfulness-based Stress Reduction | Mindfulness |
| Brown, Jodi L Constantine, Jacqueline Ong, Jessica M Mathers and James T Decker | 2017 | Compassion fatigue and mindfulness: Comparing mental health professionals and MSW student interns | Journal of evidence-informed social work |
| Buchholz, Laura | 2015 | Exploring the promise of mindfulness as medicine | Jama |
| Bueno, Viviane Freire, Elisa H Kozasa, Maria Aparecida da Silva, Tânia Maria Alves, Mario Rodrigues Louzã and Sabine Pompéia | 2015 | Mindfulness meditation improves mood, quality of life, and attention in adults with attention deficit hyperactivity disorder | BioMed research international |
| Burton, Amy, Catherine Burgess, Sarah Dean, Gina Z Koutsopoulou and Siobhan Hugh‐Jones | 2017 | How effective are mindfulness‐based interventions for reducing stress among healthcare professionals? A systematic review and meta‐analysis | Stress and Health |
| Butler, Rachel M, Matthew T Boden, Thomas M Olino, Amanda S Morrison, Philippe R Goldin, James J Gross and Richard G Heimberg | 2018 | Emotional clarity and attention to emotions in cognitive behavioral group therapy and mindfulness-based stress reduction for social anxiety disorder | Journal of anxiety disorders |
| Butzer, Bethany, Danielle Day, Adam Potts, Connor Ryan, Sarah Coulombe, Brandie Davies, Kimberly Weidknecht, Marina Ebert, Lisa Flynn and Sat Bir S Khalsa | 2015 | Effects of a classroom-based yoga intervention on cortisol and behavior in second-and third-grade students: A pilot study | Journal of evidence-based complementary & alternative medicine |
| Cachia, Renee L, Angelika Anderson and Dennis W Moore | 2016 | Mindfulness in individuals with autism spectrum disorder: a systematic review and narrative analysis | Review Journal of Autism and Developmental Disorders |
| Cachia, Renee L, Angelika Anderson and Dennis W Moore | 2016 | Mindfulness, stress and well-being in parents of children with autism spectrum disorder: a systematic review | Journal of Child and Family Studies |
| Calam, Rachel M | 2016 | Broadening the focus of parenting interventions with mindfulness and compassion | Clinical Psychology: Science and Practice |
| Caldwell, Jon G and Phillip R Shaver | 2015 | Promoting attachment-related mindfulness and compassion: A wait-list-controlled study of women who were mistreated during childhood | Mindfulness |
| Cameron, C Daryl and Barbara L Fredrickson | 2015 | Mindfulness facets predict helping behavior and distinct helping-related emotions | Mindfulness |
| Cameron, Laurie J | 2018 | The Power of Mindfulness and Compassion | The Journal of Medical Practice Management: MPM |
| Campillo, E, JJ Ricarte, L Ros, M Nieto and JM Latorre | 2018 | Effects of the Visual and Auditory Components of a Brief Mindfulness Intervention on Mood State and on Visual and Auditory Attention and Memory Task Performance | Current Psychology |
| Campos, Daniel, Ausiàs Cebolla, Soledad Quero, Juana Bretón-López, Cristina Botella, Joaquim Soler, Javier García-Campayo, Marcelo Demarzo and Rosa María Baños | 2016 | Meditation and happiness: Mindfulness and self-compassion may mediate the meditation–happiness relationship | Personality and Individual Differences |
| Canby, Nicholas K, Ian M Cameron, Amrit T Calhoun and Gregory M Buchanan | 2015 | A brief mindfulness intervention for healthy college students and its effects on psychological distress, self-control, meta-mood, and subjective vitality | Mindfulness |
| Cardeña, Etzel, Joakim OA Sjöstedt and David Marcusson-Clavertz | 2015 | Sustained attention and motivation in Zen meditators and non-meditators | Mindfulness |
| Carletto, Sara, Martina Borghi, Diana Francone, Francesco Scavelli, Gabriella Bertino, Marco Cavallo, Simona Malucchi, Antonio Bertolotto, Francesco Oliva and Luca Ostacoli | 2016 | The efficacy of a Mindfulness Based Intervention for depressive symptoms in patients with Multiple Sclerosis and their caregivers: study protocol for a randomized controlled clinical trial | BMC neurology |
| Carrière, K, B Khoury, MM Günak and B Knäuper | 2018 | Mindfulness‐based interventions for weight loss: a systematic review and meta‐analysis | Obesity Reviews |
| Cash, Therese Verkerke, Vanessa Sepopo Ekouevi, Christopher Kilbourn and Sarah K Lageman | 2016 | Pilot study of a mindfulness-based group intervention for individuals with Parkinson’s disease and their caregivers | Mindfulness |
| Cavicchioli, Marco, Mariagrazia Movalli and Cesare Maffei | 2019 | Difficulties with emotion regulation, mindfulness, and substance use disorder severity: the mediating role of self-regulation of attention and acceptance attitudes | The American journal of drug and alcohol abuse |
| Cheang, Rachael, Anna Gillions and Elizabeth Sparkes | 2019 | Do Mindfulness-Based Interventions Increase Empathy and Compassion in Children and Adolescents: A Systematic Review | Journal of Child and Family Studies |
| Chen and Eyoun | 2021 | Do mindfulness and perceived organizational support work? Fear of COVID-19 on restaurant frontline employees’ job insecurity and emotional exhaustion | International Journal of Hospitality Management |
| Chen, Siyin and Christian H Jordan | 2018 | Incorporating Ethics Into Brief Mindfulness Practice: Effects on Well-Being and Prosocial Behavior | Mindfulness |
| Chen, Xie and Huang | 2021 | Resilience of vocational students with disadvantaged characteristics in China: The role of mindfulness | Children and Youth Services Review |
| Cheung, Ke and Ng | 2020 | Dispositional mindfulness and mental health in Chinese emerging adults: A multilevel model with emotion dysregulation as a mediator | PloS One |
| Cheung, Rebecca YM and Melody CY Ng | 2018 | Mindfulness and symptoms of depression and anxiety: The underlying roles of awareness, acceptance, impulse control, and emotion regulation | Mindfulness |
| Chi, Xinli, Ai Bo, Tingting Liu, Peichao Zhang and Iris Chi | 2018 | Effects of Mindfulness-Based Stress Reduction on Depression in Adolescents and Young Adults: A Systematic Review and Meta-Analysis | Frontiers in psychology |
| Chimiklis, Alyssa L, Victoria Dahl, Angela P Spears, Kelly Goss, Katie Fogarty and Anil Chacko | 2018 | Yoga, mindfulness, and meditation interventions for youth with ADHD: Systematic review and meta-analysis | Journal of Child and Family Studies |
| Choi, Ellen and Jutta Tobias | 2015 | Mind the gap: The link between mindfulness and performance at work needs more attention | Industrial and Organizational Psychology |
| Christopher, Michael S, Richard J Goerling, Brant S Rogers, Matthew Hunsinger, Greg Baron, Aaron L Bergman and David T Zava | 2016 | A pilot study evaluating the effectiveness of a mindfulness-based intervention on cortisol awakening response and health outcomes among law enforcement officers | Journal of Police and Criminal Psychology |
| Chu, Che-Sheng, Brendon Stubbs, Tien-Yu Chen, Chia-Hung Tang, Dian-Jeng Li, Wei-Cheng Yang, Ching-Kuan Wu, André F Carvalho, Eduard Vieta and David J Miklowitz | 2018 | The effectiveness of adjunct mindfulness-based intervention in treatment of bipolar disorder: a systematic review and meta-analysis | Journal of affective disorders |
| Chumachenko, Cali, Rosal, Allison, Person, Ziedonis, Nephew, Moore, Zhang and King | 2021 | Keeping weight off: Mindfulness-Based Stress Reduction alters amygdala functional connectivity during weight loss maintenance in a randomized control trial | Plos one |
| Chung, Arlene S, Jon Smart, Michael Zdradzinski, Sarah Roth, Alecia Gende, Kylie Conroy and Nicole Battaglioli | 2018 | Educator toolkits on second victim syndrome, mindfulness and meditation, and positive psychology: The 2017 Resident Wellness Consensus Summit | Western Journal of Emergency Medicine |
| Conversano, Di Giuseppe, Miccoli, Ciacchini, Gemignani and Orrù | 2020 | Mindfulness, age and gender as protective factors against psychological distress during Covid-19 pandemic | Frontiers in psychology |
| Cifu, Gabriella, Melinda C Power, Sarah Shomstein and Hannah Arem | 2018 | Mindfulness-based interventions and cognitive function among breast cancer survivors: a systematic review | BMC cancer |
| Clarkson, Melanie, Gary Heads, Denyse Hodgson and Heidi Probst | 2019 | Does the intervention of mindfulness reduce levels of burnout and compassion fatigue and increase resilience in pre-registration students? A pilot study | Radiography |
| Cohen, Samantha CL, Danielle J Harvey, Rebecca H Shields, Grant S Shields, Roxanne N Rashedi, Daniel J Tancredi, Kathleen Angkustsiri, Robin L Hansen and Julie B Schweitzer | 2018 | Effects of Yoga on attention, impulsivity, and hyperactivity in preschool-aged children with attention-deficit hyperactivity disorder symptoms | Journal of Developmental & Behavioral Pediatrics |
| Colgan, Dana Dharmakaya, Michael Christopher, Paul Michael and Helané Wahbeh | 2016 | The body scan and mindful breathing among veterans with PTSD: type of intervention moderates the relationship between changes in mindfulness and post-treatment depression | Mindfulness |
| Collins, Rebecca N and Naoko Kishita | 2018 | The effectiveness of mindfulness-and acceptance-based interventions for informal caregivers of people with dementia: a meta-analysis | The Gerontologist |
| Cooper, David, Keong Yap and Luisa Batalha | 2018 | Mindfulness-based interventions and their effects on emotional clarity: A systematic review and meta-analysis | Journal of affective disorders |
| Corsica, Joyce, Rebecca Wilson, Megan Hood and Lauren Bradley | 2016 | Mindfulness in a weight loss intervention: Some utility and some challenges | Obesity |
| Cramer, Holger, Daniela Quinker, Karen Pilkington, Heather Mason, Jon Adams and Gustav Dobos | 2019 | Associations of yoga practice, health status, and health behavior among yoga practitioners in Germany—Results of a national cross-sectional survey | Complementary therapies in medicine |
| Cramer, Holger, Romy Lauche, Heidemarie Haller, Jost Langhorst and Gustav Dobos | 2016 | Mindfulness-and acceptance-based interventions for psychosis: a systematic review and meta-analysis | Global advances in health and medicine |
| Crane, Rebecca Susan, Judson Brewer, C Feldman, Jon Kabat-Zinn, S Santorelli, J Mark G Williams and W Kuyken | 2017 | What defines mindfulness-based programs? The warp and the weft | Psychological medicine |
| Crescentini, Cristiano, Viviana Capurso, Samantha Furlan and Franco Fabbro | 2016 | Mindfulness-oriented meditation for primary school children: Effects on attention and psychological well-being | Frontiers in Psychology |
| Creswell, J David | 2017 | Mindfulness interventions | Annual review of psychology |
| Creswell, J David | 2017 | Mindfulness interventions | Annual review of psychology |
| Creswell, J. David | 2017 | Mindfulness Interventions | Annual Review of Psychology |
| Cullen, Eichel, Lindahl, Rahrig, Kini, Flahive and Britton | 2021 | The contributions of focused attention and open monitoring in mindfulness-based cognitive therapy for affective disturbances: A 3-armed randomized dismantling trial | Plos one |
| Danucalov, Marcelo AD, Elisa H Kozasa, Rui F Afonso, José CF Galduroz and José R Leite | 2017 | Yoga and compassion meditation program improve quality of life and self‐compassion in family caregivers of A lzheimer's disease patients: A randomized controlled trial | Geriatrics & gerontology international |
| Davidson, Richard J and Alfred W Kaszniak | 2015 | Conceptual and methodological issues in research on mindfulness and meditation | American Psychologist |
| Daya, Zahra and Jasmine Heath Hearn | 2018 | Mindfulness interventions in medical education: A systematic review of their impact on medical student stress, depression, fatigue and burnout | Medical teacher |
| de Abreu Costa, Marianna, Gabriela Santos D’Alò de Oliveira, Tiago Tatton-Ramos, Gisele Gus Manfro and Giovanni Abrahão Salum | 2019 | Anxiety and Stress-Related Disorders and Mindfulness-Based Interventions: a Systematic Review and Multilevel Meta-analysis and Meta-Regression of Multiple Outcomes | Mindfulness |
| de Bruin, Esther I, J Esi van der Zwan and Susan M Bögels | 2016 | A RCT comparing daily mindfulness meditations, biofeedback exercises, and daily physical exercise on attention control, executive functioning, mindful awareness, self-compassion, and worrying in stressed young adults | Mindfulness |
| de Bruin, Esther I, Renée Meppelink and Susan M Bögels | 2015 | Mindfulness in higher education: Awareness and attention in university students increase during and after participation in a mindfulness curriculum course | Mindfulness |
| de Vibe, Michael, Ida Solhaug, Jan H Rosenvinge, Reidar Tyssen, Adam Hanley and Eric Garland | 2018 | Six-year positive effects of a mindfulness-based intervention on mindfulness, coping and well-being in medical and psychology students; Results from a randomized controlled trial | PloS one |
| de Vibe, Michael, Ida Solhaug, Reidar Tyssen, Oddgeir Friborg, Jan H Rosenvinge, Tore Sørlie, Even Halland and Arild Bjørndal | 2015 | Does personality moderate the effects of mindfulness training for medical and psychology students? | Mindfulness |
| Decker, James T, Jodi L Constantine Brown, Jacqueline Ong and Crystal A Stiney-Ziskind | 2015 | Mindfulness, compassion fatigue, and compassion satisfaction among social work interns | Social Work and Christianity |
| DeLuca, Sarah M, Alex R Kelman and Lynn C Waelde | 2018 | A systematic review of ethnoracial representation and cultural adaptation of mindfulness-and meditation-based interventions | Psychological Studies |
| Deplus, Sandrine, Joël Billieux, Chantal Scharff and Pierre Philippot | 2016 | A mindfulness-based group intervention for enhancing self-regulation of emotion in late childhood and adolescence: A pilot study | International Journal of Mental Health and Addiction |
| Dhandra, Tavleen Kaur | 2019 | Achieving triple dividend through mindfulness: More sustainable consumption, less unsustainable consumption and more life satisfaction | Ecological Economics |
| Dhillon, Anjulie, Elizabeth Sparkes and Rui V Duarte | 2017 | Mindfulness-based interventions during pregnancy: a systematic review and meta-analysis | Mindfulness |
| Dimidjian, Sona and Zindel V Segal | 2015 | Prospects for a clinical science of mindfulness-based intervention | American Psychologist |
| Donald, James N, Baljinder K Sahdra, Brooke Van Zanden, Jasper J Duineveld, Paul WB Atkins, Sarah L Marshall and Joseph Ciarrochi | 2019 | Does your mindfulness benefit others? A systematic review and meta‐analysis of the link between mindfulness and prosocial behaviour | British Journal of Psychology |
| Donald, James N, Baljinder K Sahdra, Brooke Van Zanden, Jasper J Duineveld, Paul WB Atkins, Sarah L Marshall and Joseph Ciarrochi | 2019 | Does your mindfulness benefit others? A systematic review and meta‐analysis of the link between mindfulness and prosocial behaviour | British Journal of Psychology |
| Donald, James N, Paul WB Atkins, Philip D Parker, Alison M Christie and Richard M Ryan | 2016 | Daily stress and the benefits of mindfulness: Examining the daily and longitudinal relations between present-moment awareness and stress responses | Journal of Research in Personality |
| Donaldson-Feilder, Emma, Rachel Lewis and Joanna Yarker | 2019 | What outcomes have mindfulness and meditation interventions for managers and leaders achieved? A systematic review | European Journal of Work and Organizational Psychology |
| Donovan, Elizabeth, Rachel F Rodgers, Tara M Cousineau, Kayla M McGowan, Stephanie Luk, Kayla Yates and Debra L Franko | 2016 | Brief report: Feasibility of a mindfulness and self-compassion based mobile intervention for adolescents | Journal of adolescence |
| Dorjee, Dusana | 2016 | Defining contemplative science: The metacognitive self-regulatory capacity of the mind, context of meditation practice and modes of existential awareness | Frontiers in psychology |
| Douglas, Sarah, JCH Stott, Aimee Spector, G Charlesworth, D Noone, J Payne, M Patel and E Aguirre | 2019 | Mindfulness-based cognitive therapy (MBCT) for depression in dementia: a qualitative study on patient, carer and facilitator experiences | BMC Geriatrics |
| Droit-Volet, Sylvie and Julien Heros | 2017 | Time judgments as a function of mindfulness meditation, anxiety, and mindfulness awareness | Mindfulness |
| Duarte, Joana and José Pinto-Gouveia | 2016 | Effectiveness of a mindfulness-based intervention on oncology nurses’ burnout and compassion fatigue symptoms: A non-randomized study | International journal of nursing studies |
| Duarte, Joana and José Pinto-Gouveia | 2017 | Mindfulness, self-compassion and psychological inflexibility mediate the effects of a mindfulness-based intervention in a sample of oncology nurses | Journal of Contextual Behavioral Science |
| Duarte, Rui, Annette Lloyd, Eleanor Kotas, Lazaros Andronis and Ross White | 2019 | Are acceptance and mindfulness‐based interventions ‘value for money’? Evidence from a systematic literature review | British Journal of Clinical Psychology |
| Dudley, James, Catrin Eames, John Mulligan and Naomi Fisher | 2018 | Mindfulness of voices, self‐compassion, and secure attachment in relation to the experience of hearing voices | British Journal of Clinical Psychology |
| Duffy, Jason T, Douglas A Guiffrida, Maria E Araneda, Serina MR Tetenov and Sarah C Fitzgibbons | 2017 | A qualitative study of the experiences of counseling students who participate in mindfulness-based activities in a counseling theory and practice course | International Journal for the Advancement of Counselling |
| Dunning, Darren L, Kirsty Griffiths, Willem Kuyken, Catherine Crane, Lucy Foulkes, Jenna Parker and Tim Dalgleish | 2019 | Research Review: The effects of mindfulness‐based interventions on cognition and mental health in children and adolescents–a meta‐analysis of randomized controlled trials | Journal of Child Psychology and Psychiatry |
| Dvořáková, Kamila, Mark T Greenberg and Robert W Roeser | 2019 | On the role of mindfulness and compassion skills in students' coping, well‐being, and development across the transition to college: A conceptual analysis | Stress and Health |
| Eisenbeck, Nikolett, Carmen Luciano and Sonsoles Valdivia-Salas | 2018 | Effects of a Focused Breathing Mindfulness Exercise on Attention, Memory, and Mood: The Importance of Task Characteristics | Behaviour Change |
| Eliassen, Birgit K, Tore Sørlie, Joseph Sexton and Tordis S Høifødt | 2016 | The effect of training in mindfulness and affect consciousness on the therapeutic environment for patients with psychoses: an explorative intervention study | Scandinavian journal of caring sciences |
| Ellamil, Melissa, Kieran CR Fox, Matthew L Dixon, Sean Pritchard, Rebecca M Todd, Evan Thompson and Kalina Christoff | 2016 | Dynamics of neural recruitment surrounding the spontaneous arising of thoughts in experienced mindfulness practitioners | Neuroimage |
| Emerson, Lisa-Marie, Anna Leyland, Kristian Hudson, Georgina Rowse, Pam Hanley and Siobhan Hugh-Jones | 2017 | Teaching mindfulness to teachers: a systematic review and narrative synthesis | Mindfulness |
| Epel, E, B Laraia, K Coleman-Phox, C Leung, C Vieten, L Mellin, JL Kristeller, M Thomas, N Stotland and N Bush | 2019 | Effects of a Mindfulness-Based Intervention on Distress, Weight Gain, and Glucose Control for Pregnant Low-Income Women: A Quasi-Experimental Trial Using the ORBIT Model | International journal of behavioral medicine |
| Erbe, Ryan and David Lohrmann | 2015 | Mindfulness Meditation for Adolescent Stress and Well-Being: A Systematic Review of the Literature with Implications for School Health Programs | Health Educator |
| Ewais, Tatjana, Jake Begun, Maura Kenny, Kirsty Rickett, Karen Hay, Bita Ajilchi and Steve Kisely | 2018 | A systematic review and meta-analysis of mindfulness based interventions and yoga in inflammatory bowel disease | Journal of psychosomatic research |
| Falsafi, Nasrin and Louisa Leopard | 2015 | Pilot study: use of mindfulness, self-compassion, and yoga practices with low-income and/or uninsured patients with depression and/or anxiety | Journal of Holistic Nursing |
| Felver, Joshua C, Sarah L Felver, Kathryn L Margolis, N Kathryn Ravitch, Natalie Romer and Robert H Horner | 2017 | Effectiveness and social validity of the Soles of the Feet mindfulness-based intervention with special education students | Contemporary School Psychology |
| Fiori, Francesca, Salvatore M Aglioti and Nicole David | 2017 | Interactions between body and social awareness in yoga | The Journal of Alternative and Complementary Medicine |
| Fischer, Daniel, Laura Stanszus, Sonja Geiger, Paul Grossman and Ulf Schrader | 2017 | Mindfulness and sustainable consumption: a systematic literature review of research approaches and findings | Journal of Cleaner Production |
| Fissler, Maria, Emilia Winnebeck, Titus Schroeter, Marie Gummersbach, Julia M Huntenburg, Matti Gaertner and Thorsten Barnhofer | 2016 | An investigation of the effects of brief mindfulness training on self-reported interoceptive awareness, the ability to decenter, and their role in the reduction of depressive symptoms | Mindfulness |
| Fitzgerald, Carey J and Adam K Lueke | 2017 | Mindfulness increases analytical thought and decreases just world beliefs | Current Research in Social Psychology |
| Flook, Lisa, Simon B Goldberg, Laura Pinger and Richard J Davidson | 2015 | Promoting prosocial behavior and self-regulatory skills in preschool children through a mindfulness-based kindness curriculum | Developmental psychology |
| Forseth, Bethany M, William R Boyer, Eugene C Fitzhugh and Amy Miller | 2018 | Demographic, Health Behavior, And Cardiometabolic Risk Factor Profiles In Yoga And Non-yoga Participants: Nhanes 1999-2006 | Medicine & Science in Sports & Exercise |
| Fountain-Zaragoza, Stephanie and Ruchika Shaurya Prakash | 2017 | Mindfulness training for healthy aging: impact on attention, well-being, and inflammation | Frontiers in aging neuroscience |
| Fransman, Rick and Arjan van Timmeren | 2017 | Psychological and social factors underlying pro-environmental behaviour of residents after building retrofits in the City-zen project | Energy Procedia |
| Frewen, Paul, Heather Hargraves, Jonathan DePierro, Wendy D'Andrea and Les Flodrowski | 2016 | Meditation Breath Attention Scores (MBAS): Development and investigation of an internet-based assessment of focused attention during meditation practice | Psychological assessment |
| Frostadottir, Anna Dora and Dusana Dorjee | 2019 | Effects of Mindfulness Based Cognitive Therapy (MBCT) and Compassion Focused Therapy (CFT) on Symptom Change, Mindfulness, Self-Compassion and Rumination in Clients with Depression, Anxiety and Stress | Frontiers in Psychology |
| Fulton, Cheryl L | 2018 | Self‐Compassion as a Mediator of Mindfulness and Compassion for Others | Counseling and Values |
| Fulton, Cheryl L and Craig S Cashwell | 2015 | Mindfulness‐based awareness and compassion: Predictors of counselor empathy and anxiety | Counselor Education and Supervision |
| Fung, Joey, Joanna J Kim, Joel Jin, Grace Chen, Laurel Bear and Anna S Lau | 2019 | A randomized trial evaluating school-based mindfulness intervention for ethnic minority youth: exploring mediators and moderators of intervention effects | Journal of abnormal child psychology |
| Galante, Julieta, Geraldine Dufour, Alice Benton, Emma Howarth, Maris Vainre, Timothy J Croudace, Adam P Wagner, Jan Stochl and Peter B Jones | 2016 | Protocol for the Mindful Student Study: a randomised controlled trial of the provision of a mindfulness intervention to support university students' well-being and resilience to stress | BMJ open |
| Galante, Friedrich, Dawson, Modrego-Alarcón, Gebbing, Delgado-Suárez, Gupta, Dean, Dalgleish and White | 2021 | Mindfulness-based programmes for mental health promotion in adults in nonclinical settings: A systematic review and meta-analysis of randomised controlled trials | PLoS medicine |
| Galla, Brian M | 2016 | Within-person changes in mindfulness and self-compassion predict enhanced emotional well-being in healthy, but stressed adolescents | Journal of adolescence |
| Garland, Eric L, Norman A Farb, Philippe R Goldin and Barbara L Fredrickson | 2015 | The mindfulness-to-meaning theory: extensions, applications, and challenges at the attention–appraisal–emotion interface | Psychological Inquiry |
| Garland, Eric L, Norman A Farb, Philippe R. Goldin and Barbara L Fredrickson | 2015 | Mindfulness broadens awareness and builds eudaimonic meaning: A process model of mindful positive emotion regulation | Psychological Inquiry |
| Garland, Sheila N, Codie R Rouleau, Tavis Campbell, Charles Samuels and Linda E Carlson | 2015 | The comparative impact of mindfulness-based cancer recovery (MBCR) and cognitive behavior therapy for insomnia (CBT-I) on sleep and mindfulness in cancer patients | Explore: The Journal of Science and Healing |
| Gawande, Richa, My Ngoc To, Elizabeth Pine, Todd Griswold, Timothy B Creedon, Alexandra Brunel, Angela Lozada, Eric B Loucks and Zev Schuman-Olivier | 2019 | Mindfulness Training Enhances Self-Regulation and Facilitates Health Behavior Change for Primary Care Patients: a Randomized Controlled Trial | Journal of general internal medicine |
| Geiger, Sonja M, Paul Grossman and Ulf Schrader | 2018 | Mindfulness and sustainability: Correlation or causation? | Current Opinion in Psychology |
| Geiger, Sonja M, Siegmar Otto and Ulf Schrader | 2018 | Mindfully green and healthy: An indirect path from mindfulness to ecological behavior | Frontiers in psychology |
| Godfrey, Kathryn M, Linda C Gallo and Niloofar Afari | 2015 | Mindfulness-based interventions for binge eating: a systematic review and meta-analysis | Journal of behavioral medicine |
| Goldberg, Simon B | 2018 | Why mindfulness belongs in counseling psychology: A synergistic clinical and research agenda | Counselling Psychology Quarterly |
| Goldberg, Riordan, Sun and Davidson | 2021 | The empirical status of mindfulness-based interventions: a systematic review of 44 meta-analyses of randomized controlled trials | Perspectives on Psychological Science |
| Goldberg, Simon B, Raymond P Tucker, Preston A Greene, Richard J Davidson, Bruce E Wampold, David J Kearney and Tracy L Simpson | 2018 | Mindfulness-based interventions for psychiatric disorders: a systematic review and meta-analysis | Clinical psychology review |
| Good, Darren J, Christopher J Lyddy, Theresa M Glomb, Joyce E Bono, Kirk Warren Brown, Michelle K Duffy, Ruth A Baer, Judson A Brewer and Sara W Lazar | 2016 | Contemplating mindfulness at work: An integrative review | Journal of management |
| Gothe, Neha P, Arthur F Kramer and Edward McAuley | 2017 | Hatha yoga practice improves attention and processing speed in older adults: Results from an 8-week randomized control trial | The Journal of Alternative and Complementary Medicine |
| Gotink, Rinske A, Karlijn SFM Hermans, Nicole Geschwind, Reinier De Nooij, Wouter T De Groot and Anne EM Speckens | 2016 | Mindfulness and mood stimulate each other in an upward spiral: a mindful walking intervention using experience sampling | Mindfulness |
| Gould, Laura Feagans, Jacinda K Dariotis, Mark T Greenberg and Tamar Mendelson | 2016 | Assessing fidelity of implementation (FOI) for school-based mindfulness and yoga interventions: a systematic review | Mindfulness |
| Gouveia, MJ, C Carona, MC Canavarro and H Moreira | 2016 | Self-compassion and dispositional mindfulness are associated with parenting styles and parenting stress: The mediating role of mindful parenting | Mindfulness |
| Grant, Sean, Benjamin Colaiaco, Aneesa Motala, Roberta Shanman, Marika Booth, Melony Sorbero and Susanne Hempel | 2017 | Mindfulness-based relapse prevention for substance use disorders: A systematic review and meta-analysis | Journal of addiction medicine |
| Graser, Johannes, Volkmar Höfling, Charlotte Weβlau, Adriana Mendes and Ulrich Stangier | 2016 | Effects of a 12-week mindfulness, compassion, and loving kindness program on chronic depression: A pilot within-subjects wait-list controlled trial | Journal of Cognitive Psychotherapy |
| Green, Joseph P and Katharine N Black | 2017 | Meditation-focused attention with the MBAS and solving anagrams | Psychology of Consciousness: Theory, Research, and Practice |
| Greenberg, Mark T and Joy L Mitra | 2015 | From mindfulness to right mindfulness: The intersection of awareness and ethics | Mindfulness |
| Gregory, Amber | 2015 | Yoga and mindfulness program: The effects on compassion fatigue and compassion satisfaction in social workers | Journal of Religion & Spirituality in Social Work: Social Thought |
| Gu, Jenny, Clara Strauss, Rod Bond and Kate Cavanagh | 2015 | How do mindfulness-based cognitive therapy and mindfulness-based stress reduction improve mental health and wellbeing? A systematic review and meta-analysis of mediation studies | Clinical Psychology Review |
| Gu, Jenny, Kate Cavanagh and Clara Strauss | 2018 | Investigating the specific effects of an online mindfulness-based self-help intervention on stress and underlying mechanisms | Mindfulness |
| Hadash, Yuval, Natalie Segev, Galia Tanay, Pavel Goldstein and Amit Bernstein | 2016 | The decoupling model of equanimity: theory, measurement, and test in a mindfulness intervention | Mindfulness |
| Hall, Helen G, Jill Beattie, Rosalind Lau, Christine East and Mary Anne Biro | 2016 | Mindfulness and perinatal mental health: a systematic review | Women and Birth |
| Halladay, Jillian E, Jamie L Dawdy, Isabella F McNamara, Allison J Chen, Irene Vitoroulis, Natalia McInnes and Catharine Munn | 2019 | Mindfulness for the Mental Health and Well-Being of Post-Secondary Students: A Systematic Review and Meta-Analysis | Mindfulness |
| Halland, E, M De Vibe, I Solhaug, O Friborg, JH Rosenvinge, R Tyssen, T Sørlie and A Bjørndal | 2015 | Mindfulness training improves problem-focused coping in psychology and medical students: Results from a randomized controlled trial | College Student Journal |
| Haller, Heidemarie, Maria M Winkler, Petra Klose, Gustav Dobos, Sherko Kuemmel and Holger Cramer | 2017 | Mindfulness-based interventions for women with breast cancer: an updated systematic review and meta-analysis | Acta Oncologica |
| Hanley, Adam W, Wolf E Mehling and Eric L Garland | 2017 | Holding the body in mind: Interoceptive awareness, dispositional mindfulness and psychological well-being | Journal of psychosomatic research |
| Hanley, Adam W, Yoshio Nakamura and Eric L Garland | 2018 | The Nondual Awareness Dimensional Assessment (NADA): New tools to assess nondual traits and states of consciousness occurring within and beyond the context of meditation | Psychological assessment |
| Hanson, Petra, Emma Shuttlewood, Louise Halder, Neha Shah, FT Lam, Vinod Menon and Thomas M Barber | 2018 | Application of mindfulness in a tier 3 obesity service improves eating behavior and facilitates successful weight loss | The Journal of Clinical Endocrinology & Metabolism |
| Harley, Jane | 2018 | The role of attention in therapy for children and adolescents who stutter: Cognitive behavioral therapy and mindfulness-based interventions | American journal of speech-language pathology |
| Harrington, Anne and John D Dunne | 2015 | When mindfulness is therapy: Ethical qualms, historical perspectives | American Psychologist |
| Harris, Alexis R, Patricia A Jennings, Deirdre A Katz, Rachel M Abenavoli and Mark T Greenberg | 2016 | Promoting stress management and wellbeing in educators: Feasibility and efficacy of a school-based yoga and mindfulness intervention | Mindfulness |
| Heckenberg, Rachael A, Pennie Eddy, Stephen Kent and Bradley J Wright | 2018 | Do workplace-based mindfulness meditation programs improve physiological indices of stress? A systematic review and meta-analysis | Journal of psychosomatic research |
| Hedman-Lagerlöf, Maria, Erik Hedman-Lagerlöf and Lars-Göran Öst | 2018 | The empirical support for mindfulness-based interventions for common psychiatric disorders: a systematic review and meta-analysis | Psychological medicine |
| Hensley | 2020 | Educating for sustainable development: Cultivating creativity through mindfulness | Journal of Cleaner Production |
| Hickey, Tara, Barnaby Nelson and Graham Meadows | 2017 | Application of a mindfulness and compassion‐based approach to the at‐risk mental state | Clinical psychologist |
| Hildebrandt, Lea K, Cade McCall and Tania Singer | 2017 | Differential effects of attention-, compassion-, and socio-cognitively based mental practices on self-reports of mindfulness and compassion | Mindfulness |
| Hilton, Lara, Susanne Hempel, Brett A Ewing, Eric Apaydin, Lea Xenakis, Sydne Newberry, Ben Colaiaco, Alicia Ruelaz Maher, Roberta M Shanman and Melony E Sorbero | 2016 | Mindfulness meditation for chronic pain: systematic review and meta-analysis | Annals of Behavioral Medicine |
| Hocaloski, Shea, Stacy Elliott, Lori A Brotto, Erin Breckon and Kate McBride | 2016 | A mindfulness psychoeducational group intervention targeting sexual adjustment for women with multiple sclerosis and spinal cord injury: a pilot study | Sexuality and Disability |
| Hoffman, Diane M | 2018 | Mindfulness and the cultural psychology of personhood: Challenges of self, other, and moral orientation in Haiti | Culture & Psychology |
| Horan, Kristin A and Maija B Taylor | 2018 | Mindfulness and self-compassion as tools in health behavior change: An evaluation of a workplace intervention pilot study | Journal of Contextual Behavioral Science |
| Huberty, Green, Puzia, Larkey, Laird, Vranceanu, Vlisides-Henry and Irwin | 2021 | Testing a mindfulness meditation mobile app for the treatment of sleep-related symptoms in adults with sleep disturbance: A randomized controlled trial | Plos one |
| Hülsheger, Ute R, Alina Feinholdt and Annika Nübold | 2015 | A low‐dose mindfulness intervention and recovery from work: Effects on psychological detachment, sleep quality, and sleep duration | Journal of Occupational and Organizational Psychology |
| Hunecke, Marcel and Nadine Richter | 2018 | Mindfulness, Construction of Meaning, and Sustainable Food Consumption | Mindfulness |
| Hunt, Patricia | 2017 | Review: Nurses' perceived stress and compassion following a mindfulness meditation and self compassion training | Journal of Research in Nursing |
| Hwang, Kyusic, Aeran Kwon and Changhee Hong | 2017 | A preliminary study of new positive psychology interventions: Neurofeedback-aided meditation therapy and modified positive psychotherapy | Current Psychology |
| Hwang, Yoon-Suk, Brendan Bartlett, Melissa Greben and Kirstine Hand | 2017 | A systematic review of mindfulness interventions for in-service teachers: A tool to enhance teacher wellbeing and performance | Teaching and Teacher Education |
| Jackson, Kevin T | 2018 | Interpreting the virtues of mindfulness and compassion: Contemplative practices and virtue-oriented business ethics | Humanistic Management Journal |
| Jackson, William, Emily L Zale, Stanley J Berman, Alberto Malacarne, Amy Lapidow, Michael E Schatman, Ronald Kulich and Ana-Maria Vranceanu | 2019 | Physical functioning and mindfulness skills training in chronic pain: a systematic review | Journal of pain research |
| Jarraya, Sana, Matthias Oliver Wagner, Mohamed Jarraya and Florian Azad Engel | 2019 | 12 weeks of Kindergarten-based yoga practice increases visual attention, visual-motor precision and decreases behavior of inattention and hyperactivity in 5-year-old children | Frontiers in psychology |
| Jayawardene, Wasantha P, David K Lohrmann, Ryan G Erbe and Mohammad R Torabi | 2017 | Effects of preventive online mindfulness interventions on stress and mindfulness: A meta-analysis of randomized controlled trials | Preventive medicine reports |
| Jennings, Patricia A | 2015 | Early childhood teachers’ well-being, mindfulness, and self-compassion in relation to classroom quality and attitudes towards challenging students | Mindfulness |
| Jiga, Karma, Rebekah Jane Kaunhoven and Dusana Dorjee | 2019 | Feasibility and Efficacy of an Adapted Mindfulness-Based Intervention (MBI) in Areas of Socioeconomic Deprivation (SED) | Mindfulness |
| Johnson, Blair T and Rebecca L Acabchuk | 2018 | What are the keys to a longer, happier life? Answers from five decades of health psychology research | Social Science & Medicine |
| Johnstone, Jeanette M, Chelsea Roake, Ifrah Sheikh, Ashlie Mole, Joel T Nigg and Barry Oken | 2016 | School-based mindfulness intervention for stress reduction in adolescents: Design and methodology of an open-label, parallel group, randomized controlled trial | Contemporary clinical trials communications |
| Josipovic, Zoran | 2016 | Love and compassion meditation: a nondual perspective | Annals of the New York Academy of Sciences |
| Juberg, Michael, Samuel D Spencer, Timothy J Martin, Jonas Vibell, Alexandra da Costa Ferro, Brandt Kam and Akihiko Masuda | 2019 | A Mindfulness-Based Intervention for College Students, Faculty, and Staff: A Preliminary Investigation | Clinical Case Studies |
| Kabat-Zinn, Jon | 2015 | Mindfulness | Mindfulness |
| Kabat-Zinn, Jon | 2017 | Too early to tell: the potential impact and challenges—ethical and otherwise—inherent in the mainstreaming of dharma in an increasingly dystopian world | Mindfulness |
| Kallapiran, Kannan, Siew Koo, Richard Kirubakaran and Karen Hancock | 2015 | Effectiveness of mindfulness in improving mental health symptoms of children and adolescents: a meta‐analysis | Child and Adolescent Mental Health |
| Kang, Matthew, Rob Selzer, Harry Gibbs, Katie Bourke, Abdul-Rahman Hudaib and Jo Gibbs | 2019 | Mindfulness-based intervention to reduce burnout and psychological distress, and improve wellbeing in psychiatry trainees: a pilot study | Australasian Psychiatry |
| Kaviani, Hossein and Neda Hatami | 2016 | Link between mindfulness and personality-related factors including empathy, theory of mind, openness, pro-social behaviour and suggestibility | Clinical Depression |
| Kee, Ying Hwa | 2019 | Looking East for Mindfulness: A Glimpse of Practices and Research on Shaolin Martial Arts and Related Practices to Advance Sport Psychology | Psych |
| Keller, Julia, Eric Ruthruff, Patrick Keller, Robert Hoy, Nicholas Gaspelin and Katherine Bertolini | 2017 | “Your Brain Becomes a Rainbow”: Perceptions and Traits of 4th-Graders in a School-Based Mindfulness Intervention | Journal of Research in Childhood Education |
| Kemper, Kathi J, Xiaokui Mo and Rami Khayat | 2015 | Are mindfulness and self-compassion associated with sleep and resilience in health professionals? | The Journal of Alternative and Complementary Medicine |
| Keng, Shian-Ling, Cheng Kar Phang and Tian Po Oei | 2015 | Effects of a brief mindfulness-based intervention program on psychological symptoms and well-being among medical students in Malaysia: a controlled study | International Journal of Cognitive Therapy |
| Kersemaekers, Wendy, Silke Rupprecht, Marc Wittmann, Chris Tamdjidi, Pia Falke, Rogier Donders, Anne Speckens and Niko Kohls | 2018 | A workplace mindfulness intervention may be associated with improved psychological well-being and productivity. A preliminary field study in a company setting | Frontiers in psychology |
| Khaddouma, Alexander, Kristina Coop Gordon and Jennifer Bolden | 2015 | Mindful M&M’s: Mindfulness and parent training for a preschool child with disruptive behavior disorder | Clinical Case Studies |
| Khoo, Eve-Ling, Rebecca Small, Wei Cheng, Taylor Hatchard, Brittany Glynn, Danielle B Rice, Becky Skidmore, Samantha Kenny, Brian Hutton and Patricia A Poulin | 2019 | Comparative evaluation of group-based mindfulness-based stress reduction and cognitive behavioural therapy for the treatment and management of chronic pain: A systematic review and network meta-analysis | Evidence-based mental health |
| Khoury, Bassam, Manoj Sharma, Sarah E Rush and Claude Fournier | 2015 | Mindfulness-based stress reduction for healthy individuals: A meta-analysis | Journal of psychosomatic research |
| Kiani, Behnaz, Habib Hadianfard and John T Mitchell | 2017 | The impact of mindfulness meditation training on executive functions and emotion dysregulation in an Iranian sample of female adolescents with elevated attention‐deficit/hyperactivity disorder symptoms | Australian Journal of Psychology |
| Kiburz, Kaitlin M, Tammy D Allen and Kimberly A French | 2017 | Work–family conflict and mindfulness: Investigating the effectiveness of a brief training intervention | Journal of Organizational Behavior |
| Kiken, Laura G, Eric L Garland, Karen Bluth, Olafur S Palsson and Susan A Gaylord | 2015 | From a state to a trait: Trajectories of state mindfulness in meditation during intervention predict changes in trait mindfulness | Personality and Individual differences |
| Kinnunen, Sanna M, Anne Puolakanaho, Asko Tolvanen, Anne Mäkikangas and Raimo Lappalainen | 2018 | Does mindfulness-, acceptance-, and value-based intervention alleviate burnout?—A person-centered approach | International Journal of Stress Management |
| Kirby, James N | 2016 | The role of mindfulness and compassion in enhancing nurturing family environments | Clinical Psychology: Science and Practice |
| Kirmayer, Laurence J | 2015 | Mindfulness in cultural context | Transcultural Psychiatry |
| Kline, Anna, Megan Chesin, Miriam Latorre, Rachael Miller, Lauren St Hill, Anton Shcherbakov, Arlene King, Barbara Stanley, Marc D Weiner and Alejandro Interian | 2016 | Rationale and study design of a trial of mindfulness-based cognitive therapy for preventing suicidal behavior (MBCT-S) in military veterans | Contemporary clinical trials |
| Ko, Celine M, Fran Grace, Gilbert N Chavez, Sarah J Grimley, Emily R Dalrymple and Lisa E Olson | 2018 | Effect of Seminar on Compassion on student self-compassion, mindfulness and well-being: A randomized controlled trial | Journal of American college health |
| Kohlenberg, Robert J, Mavis Tsai, Adam M Kuczynski, James R Rae, Elizabeth Lagbas, Jianne Lo and Jonathan W Kanter | 2015 | A brief, interpersonally oriented mindfulness intervention incorporating Functional Analytic Psychotherapy׳ s model of awareness, courage and love | Journal of contextual behavioral science |
| Kohler, Mark, Mallory Rawlings, April Kaeding, Siobhan Banks and Maarten A Immink | 2017 | Meditation is effective in reducing sleepiness and improving sustained attention following acute sleep restriction | Journal of Cognitive Enhancement |
| Kong, Dejun Tony | 2016 | The pathway to unethical pro-organizational behavior: Organizational identification as a joint function of work passion and trait mindfulness | Personality and Individual Differences |
| Kor, Patrick PK, Wai Tong Chien, Justina YW Liu and Claudia KY Lai | 2018 | Mindfulness-based intervention for stress reduction of family caregivers of people with dementia: a systematic review and meta-analysis | Mindfulness |
| Koren, Mary Elaine | 2017 | Mindfulness Interventions for Nursing Students: Application of Modelling and Role Modelling Theory | International Journal of Caring Sciences |
| Koszycki, Diana, Jennifer Thake, Céline Mavounza, Jean-Philippe Daoust, Monica Taljaard and Jacques Bradwejn | 2016 | Preliminary investigation of a mindfulness-based intervention for social anxiety disorder that integrates compassion meditation and mindful exposure | The Journal of Alternative and Complementary Medicine |
| Koszycki, Diana, Jennifer Thake, Céline Mavounza, Jean-Philippe Daoust, Monica Taljaard and Jacques Bradwejn | 2016 | Preliminary investigation of a mindfulness-based intervention for social anxiety disorder that integrates compassion meditation and mindful exposure | The Journal of Alternative and Complementary Medicine |
| Kotzé, Martina and Petrus Nel | 2016 | The psychometric properties of the Mindful Attention Awareness Scale (MAAS) and Freiburg Mindfulness Inventory (FMI) as measures of mindfulness and their relationship with burnout and work engagement | SA Journal of Industrial Psychology |
| Koula, Mallory J and Jennifer M Knight | 2018 | Increasing provider awareness of and recommendations for yoga and meditation classes for cancer patients | Supportive Care in Cancer |
| Kozasa, Elisa H, Joana B Balardin, João Ricardo Sato, Khallil Taverna Chaim, Shirley S Lacerda, João Radvany, Luiz Eugênio AM Mello and Edson Amaro Jr | 2018 | Effects of a 7-day meditation retreat on the brain function of meditators and non-meditators during an attention task | Frontiers in Human Neuroscience |
| Kozasa, Elisa H, Shirley S Lacerda, Carolina Menezes, B Alan Wallace, João Radvany, Luiz EAM Mello and João R Sato | 2015 | Effects of a 9-day Shamatha Buddhist meditation retreat on attention, mindfulness and self-compassion in participants with a broad range of meditation experience | Mindfulness |
| Krishnakumar, Divya, Michael R Hamblin and Shanmugamurthy Lakshmanan | 2015 | Meditation and yoga can modulate brain mechanisms that affect behavior and anxiety-A modern scientific perspective | Ancient science |
| Kudesia, Ravi S | 2019 | Mindfulness as metacognitive practice | Academy of Management Review |
| Lange, Sarah, Kai C Bormann and Jens Rowold | 2018 | Mindful leadership: mindfulness as a new antecedent of destructive and transformational leadership behavior | Gruppe. Interaktion. Organisation. Zeitschrift für Angewandte Organisationspsychologie (GIO) |
| Langer, Álvaro I, Carlos Schmidt, Rocío Mayol, Marcela Díaz, Javiera Lecaros, Edwin Krogh, Aída Pardow, Carolina Vergara, Guillermo Vergara and Bernardita Pérez-Herrera | 2017 | The effect of a mindfulness-based intervention in cognitive functions and psychological well-being applied as an early intervention in schizophrenia and high-risk mental state in a Chilean sample: study protocol for a randomized controlled trial | Trials |
| Lazarus, Irene Sheiner | 2018 | FIFTY YEARS OF TITLES: A PERSONAL IMMERSION INTO THE JOURNAL OF TRANSPERSONAL PSYCHOLOGY PART 2: FOCUSING ON MEDITATION AND TRANSPERSONAL PSYCHOTHERAPY | Journal of Transpersonal Psychology |
| Le, Thao N and Judith M Gobert | 2015 | Translating and implementing a mindfulness-based youth suicide prevention intervention in a Native American community | Journal of Child and Family Studies |
| Lee, Shayna Fox and Jacy L Young | 2018 | Mind the gap: the history and philosophy of health psychology and mindfulness | Revista Psicologia e Saúde |
| Leeuwerik, Cavanagh, Forrester, Hoadley, Jones, Lea, Rosten and Strauss | 2020 | Participant perspectives on the acceptability and effectiveness of mindfulness-based cognitive behaviour therapy approaches for obsessive compulsive disorder | PloS one |
| Lemberger‐Truelove, Matthew E, Kira J Carbonneau, David J Atencio, Almut K Zieher and Alfredo F Palacios | 2018 | Self‐Regulatory Growth Effects for Young Children Participating in a Combined Social and Emotional Learning and Mindfulness‐Based Intervention | Journal of Counseling & Development |
| Lengyel, Attila | 2015 | Mindfulness and sustainability: utilizing the tourism context | Journal of Sustainable Development |
| Leonard, Mark | 2016 | Mindfulness Meditation and Social Change: from Therapy to Wisdom and Ethics | Journal of Oriental Studies= 東洋学術研究 |
| Leyland, Anna, Georgina Rowse and L-M Emerson | 2018 | Experimental effects of mindfulness inductions on self-regulation: Systematic review and meta-analysis | Emotion |
| Li, Chunxiao, Yuxin Zhu, Mengge Zhang, Henrik Gustafsson and Tao Chen | 2019 | Mindfulness and athlete burnout: a systematic review and meta-analysis | International journal of environmental research and public health |
| Li, Jing, Ling Long, Yu Liu, Wei He and Min Li | 2016 | Effects of a mindfulness-based intervention on fertility quality of life and pregnancy rates among women subjected to first in vitro fertilization treatment | Behaviour research and therapy |
| Li, Simon Yat Ho and Daniel Bressington | 2019 | The effects of mindfulness‐based stress reduction on depression, anxiety, and stress in older adults: A systematic review and meta‐analysis | International journal of mental health nursing |
| Li, Simon Yat Ho and Daniel Bressington | 2019 | The effects of mindfulness‐based stress reduction on depression, anxiety, and stress in older adults: A systematic review and meta‐analysis | International journal of mental health nursing |
| Li, Wen, Matthew O Howard, Eric L Garland, Patricia McGovern and Michael Lazar | 2017 | Mindfulness treatment for substance misuse: A systematic review and meta-analysis | Journal of Substance Abuse Treatment |
| Lim, Daniel, Paul Condon and David DeSteno | 2015 | Mindfulness and compassion: an examination of mechanism and scalability | PloS one |
| Lindsay, Emily K and J David Creswell | 2017 | Mechanisms of mindfulness training: Monitor and Acceptance Theory (MAT) | Clinical Psychology Review |
| Liu, Zheng, Qian-lin Chen and Yu-ying Sun | 2017 | Mindfulness training for psychological stress in family caregivers of persons with dementia: a systematic review and meta-analysis of randomized controlled trials | Clinical Interventions in Aging |
| Lo, Herman HM, Simpson WL Wong, Janet YH Wong, Jerf WK Yeung, Eline Snel and Samuel YS Wong | 2017 | The effects of family-based mindfulness intervention on ADHD symptomology in young children and their parents: a randomized control trial | Journal of attention disorders |
| Lomas, Tim | 2017 | Recontextualizing mindfulness: Theravada Buddhist perspectives on the ethical and spiritual dimensions of awareness | Psychology of Religion and Spirituality |
| Lomas, Tim, Joan Carles Medina, Itai Ivtzan, Silke Rupprecht and Francisco José Eiroa-Orosa | 2018 | A systematic review and meta-analysis of the impact of mindfulness-based interventions on the well-being of healthcare professionals | Mindfulness |
| Lomas, Tim, Juan Carlos Medina, Itai Ivtzan, Silke Rupprecht and Francisco José Eiroa-Orosa | 2017 | The impact of mindfulness on the wellbeing and performance of educators: A systematic review of the empirical literature | Teaching and Teacher Education |
| Lomas, Tim, Juan Carlos Medina, Itai Ivtzan, Silke Rupprecht and Francisco José Eiroa-Orosa | 2018 | Mindfulness-based interventions in the workplace: An inclusive systematic review and meta-analysis of their impact upon wellbeing | The Journal of Positive Psychology |
| Lomas, Tim, Juan Carlos Medina, Itai Ivtzan, Silke Rupprecht, Rona Hart and Francisco José Eiroa-Orosa | 2017 | The impact of mindfulness on well-being and performance in the workplace: an inclusive systematic review of the empirical literature | European Journal of Work and Organizational Psychology |
| Long, Erin Cooke and Michael S Christian | 2015 | Mindfulness buffers retaliatory responses to injustice: A regulatory approach | Journal of Applied Psychology |
| Long, Halvorson and Lengua | 2021 | A mindfulness-based promotive coping program improves well-being in college undergraduates | Anxiety, Stress, & Coping |
| López, Angélica, Robbert Sanderman and Maya J Schroevers | 2016 | Mindfulness and self-compassion as unique and common predictors of affect in the general population | Mindfulness |
| Lord, Susan A | 2017 | Mindfulness and spirituality in couple therapy: The use of meditative dialogue to help couples develop compassion and empathy for themselves and each other | Australian and New Zealand Journal of Family Therapy |
| Lötzke, Désirée, Florian Wiedemann, Daniela Rodrigues Recchia, Thomas Ostermann, Daniel Sattler, Johannes Ettl, Marion Kiechle and Arndt Büssing | 2016 | Iyengar-Yoga compared to exercise as a therapeutic Intervention during (Neo) adjuvant therapy in women with stage I–III breast cancer: health-related quality of life, mindfulness, spirituality, life satisfaction, and cancer-related fatigue | Evidence-Based Complementary and Alternative Medicine |
| Louise, Stephanie, Molly Fitzpatrick, Clara Strauss, Susan L Rossell and Neil Thomas | 2018 | Mindfulness-and acceptance-based interventions for psychosis: our current understanding and a meta-analysis | Schizophrenia Research |
| Louise, Stephanie, Susan L Rossell and Neil Thomas | 2019 | The Acceptability, Feasibility and Potential Outcomes of an Individual Mindfulness-Based Intervention for Hearing Voices | Behavioural and cognitive psychotherapy |
| Lu, Junfei and Keith Huffman | 2017 | A Meta-Analysis of Correlations between Trait Mindfulness and Impulsivity: Implications for Counseling | International Journal for the Advancement of Counselling |
| Lu, Shuang, Juan A Rios and Chien‐Chung Huang | 2018 | Mindfulness, Emotion and Behaviour: An Intervention Study with Chinese Migrant Children | Children & Society |
| Luberto, Christina M, Nina Shinday, Rhayun Song, Lisa L Philpotts, Elyse R Park, Gregory L Fricchione and Gloria Y Yeh | 2018 | A systematic review and meta-analysis of the effects of meditation on empathy, compassion, and prosocial behaviors | Mindfulness |
| Lueke, Adam and Bryan Gibson | 2015 | Mindfulness meditation reduces implicit age and race bias: The role of reduced automaticity of responding | Social Psychological and Personality Science |
| Lunde, Linn-Heidi and Svein Skjøtskift | 2015 | Combining mindfulness meditation with cognitive behavior therapy and medication taper for hypnotic-dependent insomnia in older adults: A case study | Clinical Case Studies |
| Lutz, Antoine, Amishi P Jha, John D Dunne and Clifford D Saron | 2015 | Investigating the phenomenological matrix of mindfulness-related practices from a neurocognitive perspective | American Psychologist |
| Lutz, J, AB Brühl, H Scheerer, L Jäncke and U Herwig | 2016 | Neural correlates of mindful self-awareness in mindfulness meditators and meditation-naïve subjects revisited | Biological psychology |
| Lymeus, Freddie, Per Lindberg and Terry Hartig | 2018 | Building mindfulness bottom-up: meditation in natural settings supports open monitoring and attention restoration | Consciousness and cognition |
| Lyzwinski, Lynnette Nathalie, Liam Caffery, Matthew Bambling and Sisira Edirippulige | 2018 | A systematic review of electronic mindfulness-based therapeutic interventions for weight, weight-related behaviors, and psychological stress | Telemedicine and e-Health |
| Maglione, Margaret A, Alicia Ruelaz Maher, Brett Ewing, Benjamin Colaiaco, Sydne Newberry, Ryan Kandrack, Roberta M Shanman, Melony E Sorbero and Susanne Hempel | 2017 | Efficacy of mindfulness meditation for smoking cessation: A systematic review and meta-analysis | Addictive Behaviors |
| Mahon, Marie Ann, Lorraine Mee, Denise Brett and Maura Dowling | 2017 | Nurses' perceived stress and compassion following a mindfulness meditation and self compassion training | Journal of research in nursing |
| Mak, Catherine, Koa Whittingham, Ross Cunnington and Roslyn N Boyd | 2018 | Efficacy of mindfulness-based interventions for attention and executive function in children and adolescents—A systematic review | Mindfulness |
| Mak, Catherine, Koa Whittingham, Ross Cunnington and Roslyn N Boyd | 2018 | Effect of mindfulness yoga programme MiYoga on attention, behaviour, and physical outcomes in cerebral palsy: a randomized controlled trial | Developmental Medicine & Child Neurology |
| Mak, Winnie WS, Alan CY Tong, Sindy YC Yip, Wacy WS Lui, Floria HN Chio, Amy TY Chan and Celia CY Wong | 2018 | Efficacy and Moderation of Mobile App–Based Programs for Mindfulness-Based Training, Self-Compassion Training, and Cognitive Behavioral Psychoeducation on Mental Health: Randomized Controlled Noninferiority Trial | JMIR mental health |
| Malboeuf-Hurtubise, Catherine, Eric Lacourse, Geneviève Taylor, Mireille Joussemet and Leila Ben Amor | 2017 | A mindfulness-based intervention pilot feasibility study for elementary school students with severe learning difficulties: effects on internalized and externalized symptoms from an emotional regulation perspective | Journal of evidence-based complementary & alternative medicine |
| Malouf, Elizabeth T, Kerstin Youman, Jeffrey Stuewig, Edward A Witt and June P Tangney | 2017 | A pilot RCT of a values-based mindfulness group intervention with jail inmates: Evidence for reduction in post-release risk behavior | Mindfulness |
| Mani, Madhavan, David J Kavanagh, Leanne Hides and Stoyan R Stoyanov | 2015 | Review and evaluation of mindfulness-based iPhone apps | JMIR mHealth and uHealth |
| Mantzios, Michail | 2018 | Mindfulness and Eating Behaviour: New directions and exciting opportunities for mindfulness-based research, interventions and practices | Frontiers in psychology |
| Mantzios, Michail and Janet Clare Wilson | 2015 | Exploring mindfulness and mindfulness with self-compassion-centered interventions to assist weight loss: theoretical considerations and preliminary results of a randomized pilot study | Mindfulness |
| Mantzios, Michail, Helen Egan, Misba Hussain, Rebecca Keyte and Henna Bahia | 2018 | Mindfulness, self-compassion, and mindful eating in relation to fat and sugar consumption: an exploratory investigation | Eating and Weight Disorders-Studies on Anorexia, Bulimia and Obesity |
| Marshall, Rebecca Shisler, Jacqueline Laures‐Gore and Kim Love | 2018 | Brief mindfulness meditation group training in aphasia: exploring attention, language and psychophysiological outcomes | International journal of language & communication disorders |
| Martin, EC, N Galloway-Williams, MG Cox and RA Winett | 2015 | Pilot testing of a mindfulness-and acceptance-based intervention for increasing cardiorespiratory fitness in sedentary adults: a feasibility study | Journal of contextual behavioral science |
| Martin, Kristy-Jane, Nima Golijani-Moghaddam and Roshan dasNair | 2018 | Mindfulness self-help interventions for symptoms of depression, anxiety and stress: Review and meta-analysis | International Journal of Therapy And Rehabilitation |
| Martin, Kristy-Jane, Nima Golijani-Moghaddam and Roshan dasNair | 2018 | Mindfulness self-help interventions for symptoms of depression, anxiety and stress: Review and meta-analysis | International Journal of Therapy And Rehabilitation |
| Martins, Maria João, Paula Castilho, Vitor Santos and Andrew Gumley | 2017 | Schizophrenia: An Exploration of an Acceptance, Mindfulness, and Compassion‐based Group Intervention | Australian Psychologist |
| Mason, Ashley E, Elissa S Epel, Jean Kristeller, Patricia J Moran, Mary Dallman, Robert H Lustig, Michael Acree, Peter Bacchetti, Barbara A Laraia and Frederick M Hecht | 2016 | Effects of a mindfulness-based intervention on mindful eating, sweets consumption, and fasting glucose levels in obese adults: data from the SHINE randomized controlled trial | Journal of behavioral medicine |
| Mason, Ashley E, Elissa S Epel, Kirstin Aschbacher, Robert H Lustig, Michael Acree, Jean Kristeller, Michael Cohn, Mary Dallman, Patricia J Moran and Peter Bacchetti | 2016 | Reduced reward-driven eating accounts for the impact of a mindfulness-based diet and exercise intervention on weight loss: Data from the SHINE randomized controlled trial | Appetite |
| Mason, James, Andrew Meal, Ian Shaw and Gary G Adams | 2018 | Outcomes of mindfulness-based stress reduction and mindfulness-based cognitive therapy in adults with diabetes: a systematic review | Journal of Diabetes and Treatment |
| Masuda, Akihiko, Rachel D Marshall and Janet D Latner | 2018 | Mindfulness as a Moderator of the Association between Eating Disorder Cognition and Eating Disorder Behavior Among a Non-Clinical Sample of Female College Students: The Role of Ethnicity | Frontiers in psychology |
| Maynard, Brandy R, Alyssa N Wilson, Elizabeth Labuzienski and Seth W Whiting | 2018 | Mindfulness-based approaches in the treatment of disordered gambling: A systematic review and meta-analysis | Research on Social Work Practice |
| Maynard, Brandy R, Michael R Solis and Veronica Miller | 2015 | Mindfulness-based interventions for improving academic achievement, behavior and socio-emotional functioning of primary and secondary students: a systematic review | The Campbell Collaboration |
| McConville, Janet, Rachael McAleer and Andrew Hahne | 2017 | Mindfulness training for health profession students—the effect of mindfulness training on psychological well-being, learning and clinical performance of health professional students: a systematic review of randomized and non-randomized controlled trials | Explore |
| McGill, Julianne, Francesca Adler-Baeder and Priscilla Rodriguez | 2016 | Mindfully in love: A meta-analysis of the association between mindfulness and relationship satisfaction | Journal of Human Sciences and Extension |
| Meland, Anders, Vivianne Fonne, Anthony Wagstaff and Anne Marte Pensgaard | 2015 | Mindfulness-based mental training in a high-performance combat aviation population: a one-year intervention study and two-year follow-up | The International Journal of Aviation Psychology |
| Melen, Sarah, Christopher A Pepping and Analise O’Donovan | 2017 | Social foundations of mindfulness: Priming attachment anxiety reduces emotion regulation and mindful attention | Mindfulness |
| Melnychuk, Michael Christopher, Paul M Dockree, Redmond G O'Connell, Peter R Murphy, Joshua H Balsters and Ian H Robertson | 2018 | Coupling of respiration and attention via the locus coeruleus: Effects of meditation and pranayama | Psychophysiology |
| Mesmer-Magnus, Jessica, Archana Manapragada, Chockalingam Viswesvaran and Josh W Allen | 2017 | Trait mindfulness at work: A meta-analysis of the personal and professional correlates of trait mindfulness | Human Performance |
| Meyer, Eric C, Yvette Z Szabo, Sheila B Frankfurt, Nathan A Kimbrel, Bryann B DeBeer and Sandra B Morissette | 2019 | Predictors of recovery from post-deployment posttraumatic stress disorder symptoms in war veterans: The contributions of psychological flexibility, mindfulness, and self-compassion | Behaviour research and therapy |
| Mikolasek, Michael, Jonas Berg, Claudia M Witt and Jürgen Barth | 2018 | Effectiveness of mindfulness-and relaxation-based eHealth interventions for patients with medical conditions: a systematic review and synthesis | International journal of behavioral medicine |
| Millon, Guy and Andrea Halewood | 2015 | Mindfulness meditation and countertransference in the therapeutic relationship: A small‐scale exploration of therapists' experiences using grounded theory methods | Counselling and Psychotherapy Research |
| Millstein, Daniel J, Susan M Orsillo, Sarah A Hayes-Skelton and Lizabeth Roemer | 2015 | Interpersonal problems, mindfulness, and therapy outcome in an acceptance-based behavior therapy for generalized anxiety disorder | Cognitive behaviour therapy |
| Minami, Haruka, Hannah R Brinkman, Shadi Nahvi, Julia H Arnsten, Monica Rivera-Mindt, David W Wetter, Erika Litvin Bloom, Lawrence H Price, Carlos Vieira and Remington Donnelly | 2018 | Rationale, design and pilot feasibility results of a smartphone-assisted, mindfulness-based intervention for smokers with mood disorders: Project mSMART MIND | Contemporary clinical trials |
| Mistler, Lisa A, Dror Ben-Zeev, Elizabeth Carpenter-Song, Mary F Brunette and Matthew J Friedman | 2017 | Mobile Mindfulness intervention on an acute psychiatric unit: feasibility and acceptability study | JMIR mental health |
| Mitchell, John T, Elizabeth M McIntyre, Joseph S English, Michelle F Dennis, Jean C Beckham and Scott H Kollins | 2017 | A pilot trial of mindfulness meditation training for attention-deficit/hyperactivity disorder in adulthood: impact on core symptoms, executive functioning, and emotion dysregulation | Journal of attention disorders |
| Mitchell, John T, Lidia Zylowska and Scott H Kollins | 2015 | Mindfulness meditation training for attention-deficit/hyperactivity disorder in adulthood: current empirical support, treatment overview, and future directions | Cognitive and behavioral practice |
| Mohamed, Sayeda Mohamed, Nadia Bassuoni Elsharkawy and Mohamed Hammam Awad | 2017 | Mindfulness Based Intervention Program on Anxiety and Depressive Symptoms among Pregnant Women | American Journal of Nursing |
| Mohammed, Warhel Asim, Athanasios Pappous, Karthikeyan Muthumayandi and Dinkar Sharma | 2018 | The effect of mindfulness meditation on therapists’ body-awareness and burnout in different forms of practice | European Journal of Physiotherapy |
| Mokhtar, Rahmah, Nurshafiqa Sharif, Nor Azan Mat Zin and Siti Normaziah Ihsan | 2017 | Assessing Attention and Meditation Levels in Learning Process Using Brain Computer Interface | Advanced Science Letters |
| Montanari, Kate M, Cassandra L Bowe, Sherry S Chesak and Susanne M Cutshall | 2018 | Mindfulness: Assessing the Feasibility of a Pilot Intervention to Reduce Stress and Burnout | Journal of Holistic Nursing |
| Montani, Francesco, Christian Vandenberghe, Anis Khedhaouria and François Courcy | 2019 | Examining the inverted U-shaped relationship between workload and innovative work behavior: The role of work engagement and mindfulness | Human Relations |
| Montani, Francesco, Véronique Dagenais-Desmarais, Gabriele Giorgi and Simon Grégoire | 2018 | A conservation of resources perspective on negative affect and innovative work behaviour: The role of affect activation and mindfulness | Journal of Business and Psychology |
| Mooneyham, Benjamin W and Jonathan W Schooler | 2016 | Mind wandering and meta-awareness in hypnosis and meditation | Hypnosis and meditation: Towards an integrative science of conscious planes |
| Mooneyham, Benjamin W, Michael D Mrazek, Alissa J Mrazek, Kaita L Mrazek, Elliott D Ihm and Jonathan W Schooler | 2017 | An integrated assessment of changes in brain structure and function of the insula resulting from an intensive mindfulness-based intervention | Journal of Cognitive Enhancement |
| Morley, Richard H | 2017 | The impact of mindfulness meditation and self-compassion on criminal impulsivity in a prisoner sample | Journal of Police and Criminal Psychology |
| Morley, Richard H | 2018 | The impact of mindfulness meditation and self-compassion on criminal impulsivity in a prisoner sample | Journal of Police and Criminal Psychology |
| Moye, Amir Josef and Marieke K Van Vugt | 2019 | A computational model of focused attention meditation and its transfer to a sustained attention task | IEEE Transactions on Affective Computing |
| Murphy, Anne | 2016 | Mindfulness-based therapy in modern psychology: Convergence and divergence from early Buddhist thought | Contemporary Buddhism |
| Murray, Greg, Nuwan D Leitan, Michael Berk, Neil Thomas, E Michalak, Lesley Berk, Sheri L Johnson, Steven Jones, Tania Perich and Nicholas B Allen | 2015 | Online mindfulness-based intervention for late-stage bipolar disorder: pilot evidence for feasibility and effectiveness | Journal of affective disorders |
| Myers, Rachel E, Bryan T Karazsia, Eunjin Kim, Monica M Jackman, Carrie L McPherson and Nirbhay N Singh | 2018 | A telehealth parent-mediated mindfulness-based health wellness intervention for adolescents and young adults with intellectual and developmental disabilities | Advances in Neurodevelopmental Disorders |
| Nardi, Harrison, Saadeh, Webb, Wentz and Loucks | 2020 | Mindfulness and cardiovascular health: Qualitative findings on mechanisms from the mindfulness-based blood pressure reduction (MB-BP) study | PloS one |
| Ndubisi, Nelson Oly and Obaid Al‐Shuridah | 2019 | Organizational mindfulness, mindful organizing, and environmental and resource sustainability | Business Strategy and the Environment |
| Neukirch, Nadine, Sophie Reid and Alice Shires | 2019 | Yoga for PTSD and the role of interoceptive awareness: A preliminary mixed-methods case series study | European Journal of Trauma & Dissociation |
| Neves-Pereira, Mônica Souza, Marco Aurélio Bilibio de Carvalho and Cristiana de Campos Aspesi | 2018 | Mindfulness and Buddhism: Contributions of Buddhist philosophical and religious teachings to the fields of psychology and education | Gifted Education International |
| Nguyen, Michelle C, Steven G Gabbe, Kathi J Kemper, John D Mahan, Jennifer S Cheavens and Susan D Moffatt-Bruce | 2019 | Training on mind-body skills: Feasibility and effects on physician mindfulness, compassion, and associated effects on stress, burnout, and clinical outcomes | The Journal of Positive Psychology |
| Noetel, Michael, Joseph Ciarrochi, Brooke Van Zanden and Chris Lonsdale | 2017 | Mindfulness and acceptance approaches to sporting performance enhancement: A systematic review | International Review of Sport and Exercise Psychology |
| Noone, Chris and Michael J Hogan | 2018 | A randomised active-controlled trial to examine the effects of an online mindfulness intervention on executive control, critical thinking and key thinking dispositions in a university student sample | BMC psychology |
| Noordali, Farhan, Jennifer Cumming and Janice L Thompson | 2017 | Effectiveness of mindfulness-based interventions on physiological and psychological complications in adults with diabetes: a systematic review | Journal of health psychology |
| Norman, Jonna, Michael Fu, Inger Ekman, Lena Björck and Kristin Falk | 2018 | Effects of a mindfulness-based intervention on symptoms and signs in chronic heart failure: A feasibility study | European Journal of Cardiovascular Nursing |
| Norris, Catherine J, Daniel Creem, Reuben Hendler and Hedy Kober | 2018 | Brief mindfulness meditation improves attention in novices: evidence from ERPs and moderation by neuroticism | Frontiers in human neuroscience |
| Norton, Alice R, Maree J Abbott, Melissa M Norberg and Caroline Hunt | 2015 | A systematic review of mindfulness and acceptance‐based treatments for social anxiety disorder | Journal of clinical psychology |
| O'Donnell, Aislinn | 2015 | Contemplative pedagogy and mindfulness: Developing creative attention in an age of distraction | Journal of Philosophy of Education |
| O'Driscoll, Michelle, Laura J Sahm, Helen Byrne, Sharon Lambert and Stephen Byrne | 2019 | Impact of a mindfulness-based intervention on undergraduate pharmacy students' stress and distress: Quantitative results of a mixed-methods study | Currents in Pharmacy Teaching and Learning |
| O’Driscoll, Michelle, Stephen Byrne, Aoife Mc Gillicuddy, Sharon Lambert and Laura J Sahm | 2017 | The effects of mindfulness-based interventions for health and social care undergraduate students–a systematic review of the literature | Psychology, health & medicine |
| Ogata, Keizaburo, Ken Ichiro Koyama, Marie Amitani, Haruka Amitani and Akio Inui | 2018 | The Effectiveness of Cognitive Behavioral Therapy With Mindfulness and an Internet Intervention for Obesity: A Case Series | Frontiers in nutrition |
| Oman, Doug and Jill E Bormann | 2018 | Eknath Easwaran’s mantram and passage meditation as applied Indian psychology: Psycho-spiritual and health effects | Psychological Studies |
| Orellana-Rios, Claudia L, Lukas Radbruch, Martina Kern, Yesche U Regel, Andreas Anton, Shane Sinclair and Stefan Schmidt | 2018 | Mindfulness and compassion-oriented practices at work reduce distress and enhance self-care of palliative care teams: a mixed-method evaluation of an “on the job “program | BMC palliative care |
| Ortet, Pinazo, Walker, Gallego, Mezquita and Ibáñez | 2020 | Personality and nonjudging make you happier: Contribution of the Five-Factor Model, mindfulness facets and a mindfulness intervention to subjective well-being | PloS one |
| Palmeira, Lara, Marina Cunha and José Pinto-Gouveia | 2019 | Processes of change in quality of life, weight self-stigma, body mass index and emotional eating after an acceptance-, mindfulness-and compassion-based group intervention (Kg-Free) for women with overweight and obesity | Journal of Health Psychology |
| Panek, Elliot T, Joseph B Bayer, Sonya Dal Cin and Scott W Campbell | 2015 | Automaticity, mindfulness, and self-control as predictors of dangerous texting behavior | Mobile Media & Communication |
| Panno, Angelo, Mauro Giacomantonio, Giuseppe Carrus, Fridanna Maricchiolo, Sabine Pirchio and Lucia Mannetti | 2018 | Mindfulness, pro-environmental behavior, and belief in climate change: the mediating role of social dominance | Environment and Behavior |
| Parameshwaran, Ramakrishnan | 2015 | Theory and practice of chaplain's spiritual care process: A psychiatrist's experiences of chaplaincy and conceptualizing trans-personal model of mindfulness | Indian journal of psychiatry |
| Parsons, Christine E, Catherine Crane, Liam J Parsons, Lone Overby Fjorback and Willem Kuyken | 2017 | Home practice in Mindfulness-Based Cognitive Therapy and Mindfulness-Based Stress Reduction: A systematic review and meta-analysis of participants' mindfulness practice and its association with outcomes | Behaviour Research and Therapy |
| Pascoe, Michaela C, David R Thompson, Zoe M Jenkins and Chantal F Ski | 2017 | Mindfulness mediates the physiological markers of stress: systematic review and meta-analysis | Journal of psychiatric research |
| Patel, Naresh Kumar, L Nivethitha and A Mooventhan | 2018 | Effect of a Yoga Based Meditation Technique on Emotional Regulation, Self-compassion and Mindfulness in College Students | EXPLORE |
| Pedersen, Susanne, Falko F. Sniehotta, Kirby Sainsbury, Elizabeth H. Evans, Marta M. Marques, R. James Stubbs, Berit L. Heitmann and Liisa Lähteenmäki | 2018 | The complexity of self-regulating food intake in weight loss maintenance. A qualitative study among short- and long-term weight loss maintainers | Social Science & Medicine |
| Perestelo-Perez, Lilisbeth, Jorge Barraca, Wenceslao Peñate, Amado Rivero-Santana and Yolanda Alvarez-Perez | 2017 | Mindfulness-based interventions for the treatment of depressive rumination: Systematic review and meta-analysis | International Journal of Clinical and Health Psychology |
| Pintado, Sheila | 2019 | Changes in body awareness and self-compassion in clinical psychology trainees through a mindfulness program | Complementary therapies in clinical practice |
| Pinto-Gouveia, José, Sérgio A Carvalho, Lara Palmeira, Paula Castilho, Cristiana Duarte, Cláudia Ferreira, Joana Duarte, Marina Cunha, Marcela Matos and Joana Costa | 2019 | Incorporating psychoeducation, mindfulness and self-compassion in a new programme for binge eating (BEfree): Exploring processes of change | Journal of health psychology |
| Pinto‐Gouveia, José, Sérgio A Carvalho, Lara Palmeira, Paula Castilho, Cristiana Duarte, Cláudia Ferreira, Joana Duarte, Marina Cunha, Marcela Matos and Joana Costa | 2017 | BEfree: A new psychological program for binge eating that integrates psychoeducation, mindfulness, and compassion | Clinical psychology & psychotherapy |
| Pivarunas, Bernadette, Nichole R Kelly, Courtney K Pickworth, Omni Cassidy, Rachel M Radin, Lisa M Shank, Anna Vannucci, Amber B Courville, Kong Y Chen and Marian Tanofsky‐Kraff | 2015 | Mindfulness and eating behavior in adolescent girls at risk for type 2 diabetes | International Journal of Eating Disorders |
| Poissant, Hélène, Adrianna Mendrek, Nadine Talbot, Bassam Khoury and Jennifer Nolan | 2019 | Behavioral and Cognitive Impacts of Mindfulness-Based Interventions on Adults with Attention-Deficit Hyperactivity Disorder: A Systematic Review | Behavioural neurology |
| Poissant, Hélène, Adrianna Mendrek, Nadine Talbot, Bassam Khoury and Jennifer Nolan | 2019 | Behavioral and Cognitive Impacts of Mindfulness-Based Interventions on Adults with Attention-Deficit Hyperactivity Disorder: A Systematic Review | Behavioural neurology |
| Potes, Angela, Gabriel Souza, Katerina Nikolitch, Romeo Penheiro, Yara Moussa, Eric Jarvis, Karl Looper and Soham Rej | 2018 | Mindfulness in severe and persistent mental illness: a systematic review | International journal of psychiatry in clinical practice |
| Pradhan, Basant | 2018 | Combining Mindfulness Based Cognitive Therapy (MBCT) with Brain Stimulation Using Concurrent Repetitive Transcranial Magnetic Stimulation (rTMS) and Focused Attention Meditation During the rTMS Session for Refractory Depression: A Case Report | EC Neurology |
| Purser, Ronald E and Joseph Milillo | 2015 | Mindfulness revisited: A Buddhist-based conceptualization | Journal of Management Inquiry |
| Qamar, Osama and Ahrar Husain | 2017 | Upper Primary School Students Health and Awareness of Yoga as a Cure: An Exploratory Study | Educational Quest |
| Qu, Weina, Huihui Zhang, Wenguo Zhao, Kan Zhang and Yan Ge | 2016 | The effect of cognitive errors, mindfulness and personality traits on pedestrian behavior in a Chinese sample | Transportation research part F: traffic psychology and behaviour |
| Quaglia, Jordan T, Kirk Warren Brown, Emily K Lindsay, J David Creswell and Robert J Goodman | 2015 | From conceptualization to operationalization of mindfulness | Handbook of mindfulness: Theory, research, and practice |
| Quaglia, Jordan T, Robert J Goodman and Kirk Warren Brown | 2016 | Trait mindfulness predicts efficient top‐down attention to and discrimination of facial expressions | Journal of personality |
| Quan, Peng, Wenna Wang, Chengjing Chu and Lingfengz Hou | 2018 | Seven days of mindfulness-based cognitive therapy improves attention and coping style | Social Behavior and Personality: an international journal |
| Rahl, Hayley A, Emily K Lindsay, Laura E Pacilio, Kirk W Brown and J David Creswell | 2017 | Brief mindfulness meditation training reduces mind wandering: the critical role of acceptance | Emotion |
| Ramler, Taylor R, Linda R Tennison, Julie Lynch and Patsy Murphy | 2016 | Mindfulness and the college transition: the efficacy of an adapted mindfulness-based stress reduction intervention in fostering adjustment among first-year students | Mindfulness |
| Randal, Chloe, Daniel Pratt and Sandra Bucci | 2015 | Mindfulness and self-esteem: a systematic review | Mindfulness |
| Rao, Nisha and Kathi J Kemper | 2017 | Online training in specific meditation practices improves gratitude, well-being, self-compassion, and confidence in providing compassionate care among health professionals | Journal of evidence-based complementary & alternative medicine |
| Reb, Jochen, Jayanth Narayanan and Zhi Wei Ho | 2015 | Mindfulness at work: Antecedents and consequences of employee awareness and absent-mindedness | Mindfulness |
| Reid, Caroline, Freya Gill, Nick Gore and Serena Brady | 2016 | New ways of seeing and being: evaluating an acceptance and mindfulness group for parents of young people with intellectual disabilities who display challenging behaviour | Journal of Intellectual Disabilities |
| Reina and Kudesia | 2020 | Wherever you go, there you become: How mindfulness arises in everyday situations | Organizational Behavior and Human Decision Processes |
| Ribeiro, Letícia, Rachel M Atchley and Barry S Oken | 2018 | Adherence to practice of mindfulness in novice meditators: practices chosen, amount of time practiced, and long-term effects following a mindfulness-based intervention | Mindfulness |
| Ricarte, JJ, L Ros, JM Latorre and MT Beltrán | 2015 | Mindfulness-based intervention in a rural primary school: Effects on attention, concentration and mood | International Journal of Cognitive Therapy |
| Richard, Véronique, Wayne Halliwell and Gershon Tenenbaum | 2017 | Effects of an Improvisation Intervention on Elite Figure Skaters’ Performance, Self Esteem, Creativity, and Mindfulness Skills | The Sport Psychologist |
| Riley, Kristen E and Seth Kalichman | 2015 | Mindfulness-based stress reduction for people living with HIV/AIDS: preliminary review of intervention trial methodologies and findings | Health psychology review |
| Rivest-Gadbois, Emmanuelle and Marie-Hélène Boudrias | 2019 | What are the known effects of yoga on the brain in relation to motor performances, body awareness and pain? A narrative review | Complementary therapies in medicine |
| Roberts, Lisa and Susanne Montgomery | 2016 | Mindfulness-based intervention for perinatal grief education and reduction among poor women in Chhattisgarh, India: A pilot study | Interdisciplinary journal of best practices in global development |
| Rodríguez Carvajal, Raquel, Carlos Alberto García Rubio, David Paniagua, Gustavo García-Diex and Sara de Rivas Hermosilla | 2016 | Mindfulness Integrative Model (MIM): Cultivating positive states of mind towards oneself and the others through mindfulness and self-compassion | anales de psicología |
| Roeser, Robert W and Jacquelynne S Eccles | 2015 | Mindfulness and compassion in human development: introduction to the special section | Developmental Psychology |
| Rogers, Jeffrey M, Madeleine Ferrari, Kylie Mosely, Cathryne P Lang and Leah Brennan | 2017 | Mindfulness‐based interventions for adults who are overweight or obese: a meta‐analysis of physical and psychological health outcomes | Obesity reviews |
| Rönnlund, Michael, Antonina Koudriavtseva, Linnea Germundsjö, Terese Eriksson, Elisabeth Åström and Maria Grazia Carelli | 2018 | Mindfulness promotes a more balanced time perspective: correlational and intervention-based evidence | Mindfulness |
| Rooks, Joshua D, Alexandra B Morrison, Merissa Goolsarran, Scott L Rogers and Amishi P Jha | 2017 | “We Are Talking About Practice”: the Influence of Mindfulness vs. Relaxation Training on Athletes’ Attention and Well-Being over High-Demand Intervals | Journal of Cognitive Enhancement |
| Rosen, Helen | 2017 | From Innovative to Conventional: Transformations of Practice in Psychology and Meditation | Journal of the International Association of Buddhist Universities (JIABU) |
| Rosenkranz, Melissa A, John D Dunne and Richard J Davidson | 2019 | The next generation of mindfulness-based intervention research: what have we learned and where are we headed? | Current opinion in psychology |
| Rosenstreich, Eyal and Lital Ruderman | 2016 | Not sensitive, yet less biased: A signal detection theory perspective on mindfulness, attention, and recognition memory | Consciousness and cognition |
| Rosenstreich, Eyal, Uzi Levi and Roni Laslo-Roth | 2018 | A matter of (inner) balance: the association between facets of mindfulness, attention deficit, and postural stability | Mindfulness |
| Röthlin, Philipp, Daniel Birrer, Stephan Horvath and Martin Grosse Holtforth | 2016 | Psychological skills training and a mindfulness-based intervention to enhance functional athletic performance: design of a randomized controlled trial using ambulatory assessment | BMC psychology |
| Rowe, Angela C, Laura Shepstone, Katherine B Carnelley, Kate Cavanagh and Abigail Millings | 2016 | Attachment security and self-compassion priming increase the likelihood that first-time engagers in mindfulness meditation will continue with mindfulness training | Mindfulness |
| Rudaz, Myriam, Michael P Twohig, Clarissa W Ong and Michael E Levin | 2017 | Mindfulness and acceptance-based trainings for fostering self-care and reducing stress in mental health professionals: A systematic review | Journal of Contextual Behavioral Science |
| Ruffault, Alexis, Sébastien Czernichow, Martin S Hagger, Margot Ferrand, Nelly Erichot, Claire Carette, Emilie Boujut and Cécile Flahault | 2017 | The effects of mindfulness training on weight-loss and health-related behaviours in adults with overweight and obesity: A systematic review and meta-analysis | Obesity research & clinical practice |
| Ruiz‐Fernández, Ortíz‐Amo, Ortega‐Galán, Ibáñez‐Masero, Rodríguez‐Salvador and Ramos‐Pichardo | 2020 | Mindfulness therapies on health professionals | International journal of mental health nursing |
| Russell, Beth S, Morica Hutchison and Alaina Fusco | 2019 | Emotion regulation outcomes and preliminary feasibility evidence from a mindfulness intervention for adolescent substance use | Journal of Child & Adolescent Substance Abuse |
| Russell, Lahiru, Anna Ugalde, Donna Milne, David Austin and Patricia M Livingston | 2018 | Digital characteristics and dissemination indicators to optimize delivery of internet-supported mindfulness-based interventions for people with a chronic condition: systematic review | JMIR mental health |
| Şakiroğlu, Mehmet, Gülten Gülada, Seçkin Uğurcan, Nesrin Kara and Tarık Gandur | 2017 | The Mediator Effect of Mindfulness Awareness on The Relationship Between Nomophobia and Academic University Adjustment Levels in College Students | International Journal of Psycho-Educational Sciences |
| Sala, Margarita, Catherine Rochefort, P. Priscilla Lui and Austin S. Baldwin | 2019 | Trait mindfulness and health behaviours: a meta-analysis | Health Psychology Review |
| Sala, Shankar Ram, Vanzhula and Levinson | 2020 | Mindfulness and eating disorder psychopathology: A meta‐analysis | International Journal of Eating Disorders |
| Salem-Guirgis, Sandra, Carly Albaum and Jonathan A Weiss | 2018 | Systematic Review of Mindfulness Interventions and Mindfulness Measures Used With the Autism Population | Journal on Developmental Disabilities |
| Salmoirago-Blotcher, Elena, Carla Rich, Rochelle K Rosen, Shira Dunsiger, Aadia Rana and Michael P Carey | 2017 | Phone-delivered mindfulness training to promote medication adherence and reduce sexual risk behavior among persons living with HIV: design and methods | Contemporary clinical trials |
| Salmoirago-Blotcher, Elena, Sue Druker, Florence Meyer, Beth Bock, Sybil Crawford and Lori Pbert | 2015 | Design and methods for “Commit to Get Fit”—A pilot study of a school-based mindfulness intervention to promote healthy diet and physical activity among adolescents | Contemporary clinical trials |
| Salvo, Vera, Jean Kristeller, Jesus Montero Marin, Adriana Sanudo, Bárbara Hatzlhoffer Lourenço, Mariana Cabral Schveitzer, Vania D’Almeida, Héctor Morillo, Suely Godoy Agostinho Gimeno and Javier Garcia-Campayo | 2018 | Mindfulness as a complementary intervention in the treatment of overweight and obesity in primary health care: study protocol for a randomised controlled trial | Trials |
| Samios, Christina | 2018 | Burnout and psychological adjustment in mental health workers in rural Australia: the roles of mindfulness and compassion satisfaction | Mindfulness |
| Sanada, Kenji, Marta Alda Díez, Montserrat Salas Valero, María C Pérez-Yus, Marcelo MP Demarzo, Jesús Montero-Marín, Mauro García-Toro and Javier García-Campayo | 2017 | Effects of mindfulness-based interventions on biomarkers in healthy and cancer populations: a systematic review | BMC complementary and alternative medicine |
| Sancho, Marta, Marta De Gracia, Rita C Rodríguez, Núria Mallorquí-Bagué, Jéssica Sánchez-González, Joan Trujols, Isabel Sánchez, Susana Jiménez-Murcia and Jose M Menchón | 2018 | Mindfulness-based interventions for the treatment of substance and behavioral addictions: a systematic review | Frontiers in psychiatry |
| Sapthiang, Supakyada, William Van Gordon and Edo Shonin | 2019 | Health School-based Mindfulness Interventions for Improving Mental Health: A Systematic Review and Thematic Synthesis of Qualitative Studies | Journal of Child and Family Studies |
| Sass, Sarah M, Lauren M Early, Linda Long, Amy Burke, David Gwinn and Paul Miller | 2019 | A brief mindfulness intervention reduces depression, increases nonjudgment, and speeds processing of emotional and neutral stimuli | Mental Health & Prevention |
| Scheepers, Emke, Epstein and Lombarts | 2020 | The impact of mindfulness‐based interventions on doctors’ well‐being and performance: A systematic review | Medical education |
| Schindler, Simon, Stefan Pfattheicher and Marc‐André Reinhard | 2019 | Potential negative consequences of mindfulness in the moral domain | European Journal of Social Psychology |
| Schmalzl, Laura, Chivon Powers, Anthony P Zanesco, Neil Yetz, Erik J Groessl and Clifford D Saron | 2018 | The effect of movement-focused and breath-focused yoga practice on stress parameters and sustained attention: A randomized controlled pilot study | Consciousness and cognition |
| Schnepper, Rebekka, Anna Richard, Frank H Wilhelm and Jens Blechert | 2019 | A combined mindfulness–prolonged chewing intervention reduces body weight, food craving, and emotional eating | Journal of consulting and clinical psychology |
| Schonert-Reichl, Kimberly A, Eva Oberle, Molly Stewart Lawlor, David Abbott, Kimberly Thomson, Tim F Oberlander and Adele Diamond | 2015 | Enhancing cognitive and social–emotional development through a simple-to-administer mindfulness-based school program for elementary school children: A randomized controlled trial | Developmental psychology |
| Schumer, Maya C, Emily K Lindsay and J David Creswell | 2018 | Brief mindfulness training for negative affectivity: A systematic review and meta-analysis | Journal of Consulting and Clinical Psychology |
| Schussler, Deborah L, Mark Greenberg, Anna DeWeese, Damira Rasheed, Anthony DeMauro, Patricia A Jennings and Joshua Brown | 2018 | Stress and release: Case studies of teacher resilience following a mindfulness-based intervention | American Journal of Education |
| Schwager, Inge TL, Ute R Hülsheger and Jonas WB Lang | 2016 | Be aware to be on the square: Mindfulness and counterproductive academic behavior | Personality and Individual Differences |
| Scott-Hamilton, John and Nicola S Schutte | 2016 | The role of adherence in the effects of a mindfulness intervention for competitive athletes: Changes in mindfulness, flow, pessimism, and anxiety | Journal of Clinical Sport Psychology |
| Scott-Sheldon, Lori AJ, Brittany L Balletto, Marissa L Donahue, Melissa M Feulner, Dean G Cruess, Elena Salmoirago-Blotcher, Rena R Wing and Michael P Carey | 2019 | Mindfulness-Based Interventions for Adults Living with HIV/AIDS: A Systematic Review and Meta-analysis | AIDS and Behavior |
| Scott-Sheldon, Lori AJ, Emily C Gathright, Marissa L Donahue, Brittany Balletto, Melissa M Feulner, Julie DeCosta, Dean G Cruess, Rena R Wing, Michael P Carey and Elena Salmoirago-Blotcher | 2019 | Mindfulness-Based Interventions for Adults with Cardiovascular Disease: A Systematic Review and Meta-Analysis | Annals of Behavioral Medicine |
| Scott‐Hamilton, John, Nicola S Schutte and Rhonda F Brown | 2016 | Effects of a mindfulness intervention on sports‐anxiety, pessimism, and flow in competitive cyclists | Applied Psychology: Health and Well‐Being |
| Seabrook, Kelly, Foley, Theiler, Thomas, Wadley and Nedeljkovic | 2020 | Understanding how virtual reality can support mindfulness practice: Mixed methods study | Journal of medical Internet research |
| Seetee, Somsong, Sangthong Terathongkum, Wantana Maneesriwongul and SA Vallipakorn | 2016 | Effect of Pulmonary Rehabilitation Program with Meditation on Perceived Self-Efficacy, Pulmonary Rehabilitation Behavior, Exercise Tolerance, and Dyspnea in Patients with Chronic Obstructive Pulmonary Disease | Journal of the Medical Association of Thailand= Chotmaihet thangphaet |
| Serfaty, Shirley, Grace Gale, Matthew Beadman, Brett Froeliger and Sunjeev K Kamboj | 2018 | Mindfulness, acceptance and defusion strategies in smokers: a systematic review of laboratory studies | Mindfulness |
| Sevilla-Llewellyn-Jones, Julia, Olga Santesteban-Echarri, Ingrid Pryor, Patrick McGorry and Mario Alvarez-Jimenez | 2018 | Web-based mindfulness interventions for mental health treatment: systematic review and meta-analysis | JMIR mental health |
| Shadiev, Rustam, Ting-Ting Wu and Yueh-Min Huang | 2017 | Enhancing learning performance, attention, and meditation using a speech-to-text recognition application: Evidence from multiple data sources | Interactive Learning Environments |
| Shadiev, Rustam, Yueh-Min Huang and Jan-Pan Hwang | 2017 | Investigating the effectiveness of speech-to-text recognition applications on learning performance, attention, and meditation | Educational Technology Research and Development |
| Shallcross, Amanda J, Pallavi D Visvanathan, Sarah H Sperber and Zoe T Duberstein | 2018 | Waking up to the problem of sleep: Can mindfulness help? A review of theory and evidence for the effects of mindfulness for sleep | Current opinion in psychology |
| Shankland, Tessier, Strub, Gauchet and Baeyens | 2021 | Improving Mental Health and Well‐Being through Informal Mindfulness Practices: An Intervention Study | Applied Psychology: Health and Well‐Being |
| Sharf, Robert H | 2015 | Is mindfulness Buddhist?(and why it matters) | Transcultural psychiatry |
| Sharp Donahoo, Lori M, Beverly Siegrist and Dawn Garrett-Wright | 2018 | Addressing compassion fatigue and stress of special education teachers and professional staff using mindfulness and prayer | The Journal of School Nursing |
| Sharp, Jennifer E and Patricia A Jennings | 2016 | Strengthening teacher presence through mindfulness: What educators say about the cultivating awareness and resilience in education (CARE) program | Mindfulness |
| Shaw, Joanne M, Natasha Sekelja, Diana Frasca, Haryana M Dhillon and Melanie A Price | 2018 | Being mindful of mindfulness interventions in cancer: A systematic review of intervention reporting and study methodology | Psycho‐oncology |
| Shearer, Annie, Melissa Hunt, Mifta Chowdhury and Lorena Nicol | 2016 | Effects of a brief mindfulness meditation intervention on student stress and heart rate variability | International Journal of Stress Management |
| Shiyko, Mariya P, Sean Hallinan and Tatsuhiko Naito | 2017 | Effects of mindfulness training on posttraumatic growth: a systematic review and meta-analysis | Mindfulness |
| Shonin, Edo and William Van Gordon | 2016 | Thupten Jingpa on compassion and mindfulness | Mindfulness |
| Shonin, Edo, William Van Gordon, Angelo Compare, Masood Zangeneh and Mark D Griffiths | 2015 | Buddhist-derived loving-kindness and compassion meditation for the treatment of psychopathology: A systematic review | Mindfulness |
| Shorey, Ryan C, Joanna Elmquist, Michael J Gawrysiak, Scott Anderson and Gregory L Stuart | 2016 | The relationship between mindfulness and compulsive sexual behavior in a sample of men in treatment for substance use disorders | Mindfulness |
| SHOREY, Shefaly, ANG Lina and CHEE Cornelia | 2019 | A systematic mixed-studies review on mindfulness-based childbirth education programmes and maternal outcomes | Nursing Outlook |
| Shute, Rosalyn H | 2018 | Schools, mindfulness, and metacognition: A view from developmental psychology | International Journal of School & Educational Psychology |
| Sibinga, Erica MS, Lindsey Webb, Sharon R Ghazarian and Jonathan M Ellen | 2016 | School-based mindfulness instruction: an RCT | Pediatrics |
| Siebelink, Nienke M, Susan M Bögels, Lisanne M Boerboom, Noor de Waal, Jan K Buitelaar, Anne E Speckens and Corina U Greven | 2018 | Mindfulness for children with ADHD and Mindful Parenting (MindChamp): Protocol of a randomised controlled trial comparing a family Mindfulness-Based Intervention as an add-on to care-as-usual with care-as-usual only | BMC psychiatry |
| Sieverdes, John C, Zachary W Adams, Lynne Nemeth, Brenda Brunner-Jackson, Martina Mueller, Ashley Anderson, Sachin Patel, Luke Sox and Frank A Treiber | 2017 | Formative evaluation on cultural tailoring breathing awareness meditation smartphone apps to reduce stress and blood pressure | Mhealth |
| Simpson, Robert, Sharon Simpson, Karen Wood, Stewart W Mercer and Frances S Mair | 2018 | Using normalisation process theory to understand barriers and facilitators to implementing mindfulness-based stress reduction for people with multiple sclerosis | Chronic Illness |
| Simpson, Robert, Sharon Simpson, Nitish Ramparsad, Margaret Lawrence, Jo Booth and Stewart W Mercer | 2019 | Mindfulness-based interventions for mental well-being among people with multiple sclerosis: a systematic review and meta-analysis of randomised controlled trials | Journal of Neurology, Neurosurgery & Psychiatry |
| Singh, Nirbhay N, Giulio E Lancioni, Bryan T Karazsia and Rachel E Myers | 2016 | Caregiver training in mindfulness-based positive behavior supports (MBPBS): effects on caregivers and adults with intellectual and developmental disabilities | Frontiers in psychology |
| Singh, Nirbhay N, Giulio E Lancioni, Bryan T Karazsia, Joshua C Felver, Rachel E Myers and Kristen Nugent | 2016 | Effects of Samatha meditation on active academic engagement and math performance of students with attention deficit/hyperactivity disorder | Mindfulness |
| Singh, Nirbhay N, Giulio E Lancioni, Oleg N Medvedev, Rachel E Myers, Jeffrey Chan, Carrie L McPherson, Monica M Jackman and Eunjin Kim | 2018 | Comparative effectiveness of caregiver training in mindfulness-based positive behavior support (MBPBS) and positive behavior support (PBS) in a randomized controlled trial | Mindfulness |
| Siu, Angela FY, Ying Ma and Francis WY Chui | 2016 | Maternal mindfulness and child social behavior: The mediating role of the mother-child relationship | Mindfulness |
| Snaith, Nicole, Tim Schultz, Michael Proeve and Philippa Rasmussen | 2018 | Mindfulness, self-compassion, anxiety and depression measures in South Australian yoga participants: implications for designing a yoga intervention | Complementary therapies in clinical practice |
| Snaith, Nicole, Tim Schultz, Michael Proeve and Philippa Rasmussen | 2018 | Mindfulness, self-compassion, anxiety and depression measures in South Australian yoga participants: implications for designing a yoga intervention | Complementary therapies in clinical practice |
| Sohl, Stephanie Jean, Gurjeet Birdee and Roy Elam | 2016 | Complementary tools to empower and sustain behavior change: Motivational interviewing and mindfulness | American journal of lifestyle medicine |
| Solhaug, Ida, Thor E Eriksen, Michael de Vibe, Hanne Haavind, Oddgeir Friborg, Tore Sørlie and Jan H Rosenvinge | 2016 | Medical and psychology student’s experiences in learning mindfulness: Benefits, paradoxes, and pitfalls | Mindfulness |
| Soysa, Champika K and Carolyn J Wilcomb | 2015 | Mindfulness, self-compassion, self-efficacy, and gender as predictors of depression, anxiety, stress, and well-being | Mindfulness |
| Spadaro, Kathleen C and Diane F Hunker | 2016 | Exploring The effects of an online asynchronous mindfulness meditation intervention with nursing students on stress, mood, and cognition: a descriptive study | Nurse education today |
| Spijkerman, MPJ, Wendy Theresia Maria Pots and Ernst Thomas Bohlmeijer | 2016 | Effectiveness of online mindfulness-based interventions in improving mental health: A review and meta-analysis of randomised controlled trials | Clinical psychology review |
| Stanko-Kaczmarek, Maja and Lukasz D Kaczmarek | 2016 | Effects of tactile sensations during finger painting on mindfulness, emotions, and scope of attention | Creativity Research Journal |
| Stankov, Filimonau and Vujičić | 2020 | A mindful shift: an opportunity for mindfulness-driven tourism in a post-pandemic world | Tourism Geographies |
| Stevenson, Jodie C, Lisa-Marie Emerson and Abigail Millings | 2017 | The relationship between adult attachment orientation and mindfulness: A systematic review and meta-analysis | Mindfulness |
| Stjernswärd, Sigrid and Lars Hansson | 2017 | User value and usability of a web‐based mindfulness intervention for families living with mental health problems | Health & social care in the community |
| Stjernswärd, Sigrid and Lars Hansson | 2017 | Effectiveness and usability of a web-based mindfulness intervention for families living with mental illness | Mindfulness |
| Stonnington, Cynthia M, Betty Darby, Angela Santucci, Pamela Mulligan, Patricia Pathuis, Andrea Cuc, Joseph G Hentz, Nan Zhang, David Mulligan and Amit Sood | 2016 | A resilience intervention involving mindfulness training for transplant patients and their caregivers | Clinical transplantation |
| Strauss, Clara, Neil Thomas and Mark Hayward | 2015 | Can we respond mindfully to distressing voices? A systematic review of evidence for engagement, acceptability, effectiveness and mechanisms of change for mindfulness-based interventions for people distressed by hearing voices | Frontiers in psychology |
| Sultan, Nevine | 2018 | Embodied self-care: Enhancing awareness and acceptance through mindfulness-oriented expressive writing self-disclosure | Journal of Creativity in Mental Health |
| Sutcliffe, Kathleen M, Timothy J Vogus and Erik Dane | 2016 | Mindfulness in organizations: A cross-level review | Annual Review of Organizational Psychology and Organizational Behavior |
| Sutcliffe, Kathleen M, Timothy J Vogus and Erik Dane | 2016 | Mindfulness in organizations: A cross-level review | Annual Review of Organizational Psychology and Organizational Behavior |
| Svendsen, Julie Lillebostad, Katrine Valvatne Kvernenes, Agnethe Smith Wiker and Ingrid Dundas | 2017 | Mechanisms of mindfulness: Rumination and self-compassion | Nordic Psychology |
| Tak, Sanne R, Christel Hendrieckx, Giesje Nefs, Ivan Nyklíček, Jane Speight and François Pouwer | 2015 | The association between types of eating behaviour and dispositional mindfulness in adults with diabetes. Results from Diabetes MILES. The Netherlands | Appetite |
| Tang, Yi-Yuan, Britta K Hölzel and Michael I Posner | 2015 | The neuroscience of mindfulness meditation | Nature Reviews Neuroscience |
| Taylor, Billie Lever, Kate Cavanagh and Clara Strauss | 2016 | The effectiveness of mindfulness-based interventions in the perinatal period: a systematic review and meta-analysis | PloS one |
| Taylor, Cynthia, Jessica Harrison, Kyla Haimovitz, Eva Oberle, Kimberly Thomson, Kimberly Schonert-Reichl and Robert W Roeser | 2016 | Examining ways that a mindfulness-based intervention reduces stress in public school teachers: A mixed-methods study | Mindfulness |
| Tercelli, Illaria and Nuno Ferreira | 2019 | A systematic review of mindfulness based interventions for children and young people with ADHD and their parents | Global Psychiatry |
| Thompson, Isabel A, Cheryl Pence Wolf, Elisa Mott, Adrienne S Baggs, Eric S Thompson, Carmelo Callueng and Ana Puig | 2018 | Luna Yoga: A Wellness Program for Female Counselors and Counselors-in-Training to Foster Self-Awareness and Connection | Journal of Creativity in Mental Health |
| Thupten, Jinpa | 2018 | The question of mindfulness' connection with ethics and compassion | Current opinion in psychology |
| Tihanyi, Benedek T, Andrea Sági, Barbara Csala, Nóra Tolnai and Ferenc Köteles | 2016 | 'Body Awareness, Mindfulness and Affect: Does the Kind of Physical Activity Make a Difference?' | European Journal of Mental Health |
| Tihanyi, Benedek T, Petra Böőr, Lene Emanuelsen and Ferenc Köteles | 2016 | 'Mediators between Yoga Practice and Psychological Well-Being: Mindfulness, Body Awareness and Satisfaction with Body Image' | European Journal of Mental Health |
| Tkatch, Rifky, Dawn Bazarko, Shirley Musich, Lizi Wu, Stephanie MacLeod, Karen Keown, Kevin Hawkins and Ellen Wicker | 2017 | A pilot online mindfulness intervention to decrease caregiver burden and improve psychological well-being | Journal of Evidence-Based Complementary & Alternative Medicine |
| Tomasino, Barbara and Franco Fabbro | 2016 | Increases in the right dorsolateral prefrontal cortex and decreases the rostral prefrontal cortex activation after-8 weeks of focused attention based mindfulness meditation | Brain and cognition |
| Tomlinson, Eve R, Omar Yousaf, Axel D Vittersø and Lauraine Jones | 2018 | Dispositional mindfulness and psychological health: a systematic review | Mindfulness |
| Tong, Alan Chun Yat, Jessie Jing Xia Lin, Veronica Yuen Ki Cheung, Nicole Ka Man Lau, Wing Chung Chang, Sherry Kit Wa Chan, Christy Lai Ming Hui, Edwin Ho Ming Lee and Eric Yu Hai Chen | 2016 | A Low‐Intensity Mindfulness‐Based Intervention for Mood Symptoms in People with Early Psychosis: Development and Pilot Evaluation | Clinical psychology & psychotherapy |
| Tovote, K Annika, Maya J Schroevers, Evelien Snippe, Paul MG Emmelkamp, Thera P Links, Robbert Sanderman and Joke Fleer | 2017 | What works best for whom? Cognitive Behavior Therapy and Mindfulness-Based Cognitive Therapy for depressive symptoms in patients with diabetes | PloS one |
| Travis, Frederick and Niyazi Parim | 2017 | Default mode network activation and Transcendental Meditation practice: Focused Attention or Automatic Self-transcending? | Brain and cognition |
| Trowbridge, Kelly and Lisa Mische Lawson | 2016 | Mindfulness-based interventions with social workers and the potential for enhanced patient-centered care: A systematic review of the literature | Social work in health care |
| Tsai, Alice, Elizabeth K Hughes, Matthew Fuller-Tyszkiewicz, Kimberly Buck and Isabel Krug | 2017 | The differential effects of mindfulness and distraction on affect and body satisfaction following food consumption | Frontiers in psychology |
| Tsur, Noga, Nirit Berkovitz and Karni Ginzburg | 2016 | Body awareness, emotional clarity, and authentic behavior: The moderating role of mindfulness | Journal of Happiness Studies |
| Úbeda-Gómez, J, MG León-Palacios, S Escudero-Pérez, MD Barros-Albarrán, AM López-Jiménez and S Perona-Garcelán | 2015 | Relationship between self-focused attention, mindfulness and distress in individuals with auditory verbal hallucinations | Cognitive neuropsychiatry |
| Ugalde, Anna, Susan Mathers, Nicole Hennessy Anderson, Peter Hudson, Liliana Orellana and Cathy Gluyas | 2018 | A self-care, problem-solving and mindfulness intervention for informal caregivers of people with motor neurone disease: A pilot study | Palliative medicine |
| Ulrichsen, Kristine M, Tobias Kaufmann, Erlend S Dørum, Knut K Kolskår, Geneviève Richard, Dag Alnæs, Tone J Arneberg, Lars T Westlye and Jan E Nordvik | 2016 | Clinical utility of mindfulness training in the treatment of fatigue after stroke, traumatic brain injury and multiple sclerosis: a systematic literature review and meta-analysis | Frontiers in psychology |
| Vaghela, Nirav, Daxa Mishra, Jigar N Mehta, Hemal Punjabi, Hena Patel and Ishani Sanchala | 2019 | Awareness and practice of aerobic exercise and yoga among hypertensive patients in Anand city | Journal of Education and Health Promotion |
| Vago, David R, Resh S Gupta and Sara W Lazar | 2018 | Measuring cognitive outcomes in mindfulness-based intervention research: a reflection on confounding factors and methodological limitations | Current opinion in psychology |
| Valerio, Adam | 2016 | Owning mindfulness: A bibliometric analysis of mindfulness literature trends within and outside of Buddhist contexts | Contemporary Buddhism |
| Van Aalderen, J, J De Haas-de Vries and N Luiten-van de Vliert | 2016 | MINDFULNESS: A NEED FOR INTEGRATION OR FACILITATION WITHIN A CHRISTIAN VIEW ON LIFE AND PSYCHOLOGY? | Mindfulness-based cognitive therapy for recurrent depression |
| Van Dam, Nicholas T, Marieke K van Vugt, David R Vago, Laura Schmalzl, Clifford D Saron, Andrew Olendzki, Ted Meissner, Sara W Lazar, Catherine E Kerr and Jolie Gorchov | 2018 | Mind the hype: A critical evaluation and prescriptive agenda for research on mindfulness and meditation | Perspectives on Psychological Science |
| Van Dam, Nicholas T, Marieke K van Vugt, David R Vago, Laura Schmalzl, Clifford D Saron, Andrew Olendzki, Ted Meissner, Sara W Lazar, Jolie Gorchov and Kieran CR Fox | 2018 | Reiterated concerns and further challenges for mindfulness and meditation research: a reply to Davidson and Dahl | Perspectives on Psychological Science |
| van Dongen, Johanna M, Jantien van Berkel, Cécile RL Boot, Judith E Bosmans, Karin I Proper, Paulien M Bongers, Allard J Van Der Beek, Maurits W van Tulder and Marieke F van Wier | 2016 | Long-term cost-effectiveness and return-on-investment of a mindfulness-based worksite intervention: results of a randomized controlled trial | Journal of occupational and environmental medicine |
| Van Driel, CM, Anniek Stuursma, Maya J Schroevers, MJ Mourits and Geertruida H de Bock | 2019 | Mindfulness, cognitive behavioural and behaviour‐based therapy for natural and treatment‐induced menopausal symptoms: a systematic review and meta‐analysis | BJOG: An International Journal of Obstetrics & Gynaecology |
| Veehof, MM, HR Trompetter, Ernst Thomas Bohlmeijer and Karlein Maria Gertrudis Schreurs | 2016 | Acceptance-and mindfulness-based interventions for the treatment of chronic pain: a meta-analytic review | Cognitive behaviour therapy |
| Verhaeghen, Paul | 2019 | The Mindfulness Manifold: Exploring How Self-Preoccupation, Self-Compassion, and Self-Transcendence Translate Mindfulness Into Positive Psychological Outcomes | Mindfulness |
| Victorson, David, Mitchell Kentor, Carly Maletich, Rachel C Lawton, Vered Hankin Kaufman, Maria Borrero, Lauren Languido, Katherine Lewett, Hannah Pancoe and Carla Berkowitz | 2015 | Mindfulness Meditation to Promote Wellness and Manage Chronic Disease: A Systematic Review and Meta-Analysis of Mindfulness-Based Randomized Controlled Trials Relevant to Lifestyle Medicine | American Journal of Lifestyle Medicine |
| Vidic, Zeljka, Mark St. Martin and Richard Oxhandler | 2017 | Mindfulness Intervention with a US women’s NCAA Division I basketball team: impact on stress, athletic coping skills and perceptions of intervention | The Sport Psychologist |
| Vieten, Cassandra, Barbara A Laraia, Jean Kristeller, Nancy Adler, Kimberly Coleman-Phox, Nicole R Bush, Helané Wahbeh, Larissa G Duncan and Elissa Epel | 2018 | The mindful moms training: development of a mindfulness-based intervention to reduce stress and overeating during pregnancy | BMC pregnancy and childbirth |
| Viglas, Melanie and Michal Perlman | 2018 | Effects of a mindfulness-based program on young children’s self-regulation, prosocial behavior and hyperactivity | Journal of Child and Family Studies |
| Vinci, Christine, Claire A Spears, MacKenzie R Peltier and Amy L Copeland | 2016 | Facets of mindfulness mediate the relationship between depressive symptoms and smoking behavior | Mindfulness |
| Viskovich, Shelley and Linda De George-Walker | 2019 | An investigation of self-care related constructs in undergraduate psychology students: Self-compassion, mindfulness, self-awareness, and integrated self-knowledge | International Journal of Educational Research |
| Visted, Endre, Jon Vøllestad, Morten Birkeland Nielsen and Geir Høstmark Nielsen | 2015 | The impact of group-based mindfulness training on self-reported mindfulness: a systematic review and meta-analysis | Mindfulness |
| Voci, Alberto, Chiara A Veneziani and Giulia Fuochi | 2019 | Relating Mindfulness, Heartfulness, and Psychological Well-Being: the Role of Self-Compassion and Gratitude | Mindfulness |
| Waloszek, Joanna M, Orli Schwartz, Julian G Simmons, Matthew Blake, Laura Blake, Greg Murray, Monika Raniti, Ronald E Dahl, Neil O’Brien-Simpson and Paul Dudgeon | 2015 | The SENSE Study (Sleep and Education: learning New Skills Early): a community cognitive-behavioural therapy and mindfulness-based sleep intervention to prevent depression and improve cardiac health in adolescence | BMC psychology |
| Walsh, Zachary David | 2017 | Critical Theory and the Contemporary Discourse on Mindfulness | Journal of the International Association of Buddhist Universities (JIABU) |
| Wamsler, Christine and Ebba Brink | 2018 | Mindsets for sustainability: Exploring the link between mindfulness and sustainable climate adaptation | Ecological Economics |
| Wamsler, Christine, Johannes Brossmann, Heidi Hendersson, Rakel Kristjansdottir, Colin McDonald and Phil Scarampi | 2018 | Mindfulness in sustainability science, practice, and teaching | Sustainability science |
| Warren, Michael T, Laura Wray-Lake and Amy K Syvertsen | 2018 | Becoming who they want to be: A cross-national examination of value-behavior concordance and mindfulness in adolescence | The Journal of Positive Psychology |
| Watier, Nicholas and Michael Dubois | 2016 | The effects of a brief mindfulness exercise on executive attention and recognition memory | Mindfulness |
| Watt, Tessa | 2017 | Spacious awareness in Mahāyāna Buddhism and its role in the modern mindfulness movement | Contemporary Buddhism |
| Wei, Meifen, Pei-Chun Tsai, Daniel G Lannin, Yi Du and Jeritt R Tucker | 2015 | Mindfulness, psychological flexibility, and counseling self-efficacy: Hindering self-focused attention as a mediator | The Counseling Psychologist |
| Wheeler, Megan S, Diane B Arnkoff and Carol R Glass | 2016 | What is being studied as mindfulness meditation? | Nature Reviews Neuroscience |
| Whitehead, Mark, Rachel Lilley, Rachel Howell, Rhys Jones and Jessica Pykett | 2016 | (Re) Inhabiting awareness: geography and mindfulness | Social & Cultural Geography |
| Wongtongkam, Nualnong, Andrew Day, Paul Russell Ward and Anthony Harold Winefield | 2015 | The influence of mindfulness meditation on angry emotions and violent behavior on Thai technical college students | European Journal of Integrative Medicine |
| Wongtongkam, Nualnong, Branka Krivokapic-Skoko, Roderick Duncan and Mariagrazia Bellio | 2017 | The influence of a mindfulness-based intervention on job satisfaction and work-related stress and anxiety | International Journal of Mental Health Promotion |
| Yang, Chih-Hsiang and David E Conroy | 2019 | Mindfulness and physical activity: a systematic review and hierarchical model of mindfulness | International Journal of Sport and Exercise Psychology |
| Yang, Xiaozhe, Pei-Yu Cheng, Lin Lin, Yueh Min Huang and Youqun Ren | 2019 | Can an Integrated System of Electroencephalography and Virtual Reality Further the Understanding of Relationships Between Attention, Meditation, Flow State, and Creativity? | Journal of Educational Computing Research |
| Yang, Yang, Yan-Hui Liu, Hong-Fu Zhang and Jing-Ying Liu | 2015 | Effectiveness of mindfulness-based stress reduction and mindfulness-based cognitive therapies on people living with HIV: A systematic review and meta-analysis | International Journal of Nursing Sciences |
| Yazdanimehr, Reza, Abdollah Omidi, Zohreh Sadat and Hossein Akbari | 2016 | The effect of mindfulness-integrated cognitive behavior therapy on depression and anxiety among pregnant women: a randomized clinical trial | Journal of caring sciences |
| Yip, Sindy YC, Winnie WS Mak, Floria HN Chio and Rita W Law | 2017 | The mediating role of self-compassion between mindfulness and compassion fatigue among therapists in Hong Kong | Mindfulness |
| Yook, Young-Sook, Soo-Jin Kang and InKyoung Park | 2017 | Effects of physical activity intervention combining a new sport and mindfulness yoga on psychological characteristics in adolescents | International Journal of Sport and Exercise Psychology |
| Zanesco, Anthony P, Brandon G King, Chivon Powers, Rosanna De Meo, Kezia Wineberg, Katherine A MacLean and Clifford D Saron | 2019 | Modulation of event-related potentials of visual discrimination by meditation training and sustained attention | Journal of cognitive neuroscience |
| Zangi, Heidi A and Liv Haugli | 2017 | Vitality training—A mindfulness-and acceptance-based intervention for chronic pain | Patient education and counseling |
| Zeng, Xianglong, Mengdan Li, Bo Zhang and Xiangping Liu | 2015 | Revision of the Philadelphia mindfulness scale for measuring awareness and equanimity in Goenka’s Vipassana meditation with Chinese Buddhists | Journal of religion and health |
| Zeng, Xianglong, Tian PS Oei, Yiqing Ye and Xiangping Liu | 2015 | A critical analysis of the concepts and measurement of awareness and equanimity in Goenka’s Vipassana meditation | Journal of religion and health |
| Zgierska, Aleksandra E, Cindy A Burzinski, Jennifer Cox, John Kloke, Aaron Stegner, Dane B Cook, Janice Singles, Shilagh Mirgain, Christopher L Coe and Miroslav Bačkonja | 2016 | Mindfulness meditation and cognitive behavioral therapy intervention reduces pain severity and sensitivity in opioid-treated chronic low back pain: pilot findings from a randomized controlled trial | Pain Medicine |
| Zgierska, Aleksandra E, Cindy A Burzinski, Jennifer Cox, John Kloke, Janice Singles, Shilagh Mirgain, Aaron Stegner, Dane B Cook and Miroslav Bačkonja | 2016 | Mindfulness meditation-based intervention is feasible, acceptable, and safe for chronic low back pain requiring long-term daily opioid therapy | The Journal of Alternative and Complementary Medicine |
| Zhang, Jun, Rui Xu, Bo Wang and Jinxia Wang | 2016 | Effects of mindfulness-based therapy for patients with breast cancer: a systematic review and meta-analysis | Complementary therapies in medicine |
| Zhang, Junhua, Amparo Díaz-Román and Samuele Cortese | 2018 | Meditation-based therapies for attention-deficit/hyperactivity disorder in children, adolescents and adults: a systematic review and meta-analysis | Evidence-based mental health |
| Zhang, Mei-Fen, Yong-Shan Wen, Wei-Yan Liu, Li-Fen Peng, Xiao-Dan Wu and Qian-Wen Liu | 2015 | Effectiveness of mindfulness-based therapy for reducing anxiety and depression in patients with cancer: a meta-analysis | Medicine |
| Zhang, Qiuxiang, Heng Zhao and Yaning Zheng | 2019 | Effectiveness of mindfulness-based stress reduction (MBSR) on symptom variables and health-related quality of life in breast cancer patients—a systematic review and meta-analysis | Supportive Care in Cancer |
| Zhang, Xuxi, Siok Swan Tan, Irene Fierloos, Oscar Zanutto, Tamara Alhambra-Borrás, Vanja Vasiljev, Scott Bennett, Tasos Rentoumis, Antonella Buranello and Stefania Macchione | 2019 | Evaluation design of the Social Engagement Framework for Addressing the Chronic-disease-challenge (SEFAC): a mindfulness-based intervention to promote the self-management of chronic conditions and a healthy lifestyle | BMC public health |
| Zhou, Jingxuan, Ping Peng and Xiaohua Xie | 2018 | Prevalence of pain and effects of a brief mindfulness-based intervention on Chinese Community-dwelling older adults with chronic pain | Journal of community health nursing |
| Zimmermann, Fernanda F, Beverley Burrell and Jennifer Jordan | 2018 | The acceptability and potential benefits of mindfulness-based interventions in improving psychological well-being for adults with advanced cancer: a systematic review | Complementary therapies in clinical practice |
| Zou, Liye, Albert Yeung, Xinfeng Quan, Sean Boyden and Huiru Wang | 2018 | A systematic review and meta-analysis of mindfulness-based (Baduanjin) exercise for alleviating musculoskeletal pain and improving sleep quality in people with chronic diseases | International journal of environmental research and public health |
| Zou, Liye, Albert Yeung, Xinfeng Quan, Stanley Sai-Chuen Hui, Xiaoyue Hu, Jessie SM Chan, Chaoyi Wang, Sean David Boyden, Li Sun and Huiru Wang | 2018 | Mindfulness-based Baduanjin exercise for depression and anxiety in people with physical or mental illnesses: a systematic review and meta-analysis | International journal of environmental research and public health |
