# Supplementary material for: The search for scientific meaning in mindfulness research: Insights from a scoping review
Source: PLoS One. 2022 May 4;17(5):e0264924. doi: 10.1371/journal.pone.0264924 (PMC9067662; doi:10.1371/journal.pone.0264924)
Supplement: S1 File — (DOCX) [file pone.0264924.s003.docx]

# S3 Glossary of terms

| Term | Description | Disciplines using |
| --- | --- | --- |
| Acceptance | Willingness to tolerate a difficult situation | Psychology, Mindfulness, Behaviour Study |
| Attention | Attention is often subdivided into three different components: alerting (readiness in preparation for an impending stimulus, which includes tonic effects that result from spending time on a task (vigilance) and phasic effects that are due to brain changes induced by warning signals or targets); orienting (the selection of specific information from multiple sen- sory stimuli); and conflict monitoring (monitoring and resolution of conflict between computations in different neural areas, also referred to as executive attention) | Neuroscience |
| Attention control | Refers to an individual's capacity to choose what they pay attention to and what they ignore | Psychology, Behaviour Study |
| Awareness | Concern about and well-informed interest in a particular situation or development. | Psychology, Mindfulness |
| Brain and traits effects | Temporarily changes the condition of the brain and the corresponding pattern of activity or connectivity (state change), and it also alters personality traits following a longer period of practice | Psychology, Mindfulness, Behaviour Study |
| Buddhism | The world's fourth-largest religion. A widespread Asian religion or philosophy, founded by Siddartha Gautama in north-eastern India in the 5th century BC | Religion study |
| Buddhist traditions | Three main branches of Buddhism including Theravada, Mahayana, Vajrayana | Religion study |
| Canonical mindfulness | Sacred mindfulness practice officially accepted as genuine | Mindfulness |
| Category | Division of mindfulness studies regarded as having particular shared characteristics |  |
| Classification | The arrangement of studies in groups according to their observed similarities |  |
| Compassion | Sympathetic pity and concern for the sufferings or misfortunes of others | Psychology, Mindfulness, Behaviour Study, Religion Study |
| Concept | Group of terms | Linguistic |
| Cultivation | The process of trying to acquire or develop a quality or skill | Mindfulness |
| Discernment | The ability to obtain sharp perceptions or to judge well |  |
| Domain | A discipline area. A peer-developed complete set of concepts, terms, and activities that make up a body-of-knowledge | Science |
| Effortless mindfulness / Trait mindfulness | An opening to a unintention naturally compassionate, non-conceptual awake awareness that is interconnected here and now. | Mindfulness, Psychology |
| Emotion regulation | Emotion regulation refers to strategies that can influence which emotions arise and when, how long they occur, and how these emotions are experienced and expressed. | Behavioural and Cognitive Psychology |
| Ethical-mindedness | The human propensity to be concerned with morality and ethics | Mindfulness |
| Intentionality | The fact of being deliberate or purposive | Psychology, Mindfulness |
| Internet and smartphone application mindfulness interventions | Web and app-based program developed based on mindfulness interventions to bring virtual mindfulness practice to practitioners | Mindfulness |
| Langerian mindfulness | A doctrine and method that was developed by psychologist Dr. Ellen Langer. Langerian mindfulness is defined as the process of paying attention on purpose to the present moment, of being aware of novelty in experiences or situations, and of perceiving differences in contexts and events | Psychology |
| Long term effects of mindfulness | The effects of mindfulness on the brain, traits, mental well-being on practitioners who practicing mindfulness for more than 3 months | Psychology, Mindfulness |
| Long-term practitioners | Mindfulness practitioners who are practicing mindfulness for months to years | Mindfulness |
| Mechanism based approach to classification | Classification approach that divide mindfulness studies according to the effects of mindfulness on different cognitive levels | Mindfulness |
| Memory | The faculty by which the mind stores and remembers information | Psychology |
| Mindful attention | Emphasizes how people dynamically regulate their attention over time, in contrast to more static “resource” accounts that imply scarcity. Mindful attention thereby prevents people’s minds from wandering away from situations to potentially negative conceptualizing | Mindfulness, Psychology, Behaviour Study |
| Mindful conceptualising | Recognizes that concepts can be a key source of flexible responses—if people refine prior concepts to interpret their current situations. Conceptualising singles out isolated parts of situations, which prevents people from entering a holistic landscape of absorbed engagement, thereby limiting their developing expertise | Mindfulness, Psychology, Behaviour Study |
| Mindful metacognition | The processes by which people monitor and adjust their information processing | Mindfulness, Psychology, Behaviour Study |
| Mindfulness in Buddhism | Mindfulness derives from sati, a significant element of Buddhist traditions, and based on Zen, Vipassanā, and Tibetan meditation techniques. Buddhist traditions explain what constitutes mindfulness such as how past, present and future moments arise and cease as momentary sense impressions and mental phenomena | Psychology, Religion Study, Mindfulness |
| Mindfulness intervention retreats and brief interventions | Mindfulness practices that involve several weeks of full-time intensive mindfulness training | Mindfulness |
| Mindfulness-based stress reduction (MBSR) | Mindfulness-based stress reduction (MBSR) is a mindfulness-based program developed by Jon Kabat-Zinn at the University of Massachusetts Medical Center, which uses a combination of mindfulness meditation, body awareness, and yoga to help people become more mindful | Psychology, Mindfulness |
| Mindfulness-related interventions | Interventions including predominantly mindfulness elements | Psychology, Mindfulness |
| Non evaluative | Not involving evaluation or judgement; concerned only with fact |  |
| Non judgemental / non judgement | Not judgemental; avoiding moral judgements | Psychology, Mindfulness |
| Occurrence based approach to classification | Classification approach that divide mindfulness studies according to the frequency and the intetionality that mindfulness happen | Mindfulness |
| Opening, accepting, non-judgemental orientation | Aware and accept the situations and external events as the way it is without judgement or criticise | Mindfulness |
| Positive psychology | The scientific study of the "good life", or the positive aspects of the human experience that make life worth living | Psychology |
| Present-centred | Present centred awareness is a practice and a technique where practitioners are present and aware for the experience of life | Psychology, Mindfulness |
| Procedure | A series of actions conducted in a certain order or manner | Mindfulness |
| Retention | The continued possession, use, or control of something | Psychology |
| Self-awareness | The experience of one's own personality or individuality. Self-awareness is how an individual consciously knows and understands their own character, feelings, motives, and desires | Psychology, Philosophy |
| Self-regulated attention | The individual’s ability to adjust physiological arousal to situational conditions | Biology, Psychology |
| Short-term practitioners | Mindfulness practitioners who are practicing mindfulness for weeks to months | Mindfulness |
| State of mind and brain | The reliable patterns of brain activity that involve the activation and/or connectivity of multiple large-scale brain networks | Neuroscience |
| Term | Word or group of words included together | Linguistic |
| Therapeutic Effect | Therapeutic effect refers to the response(s) after a treatment of any kind, the results of which are judged to be useful or favorable. | Psychology |
| Therapeutic modality | Therapeutic modalities represent the administration of thermal, mechanical, electromagnetic, and light energies for a specific therapeutic effect; for example, to decrease pain, increase range of motion (ROM), improve tissue healing, or improve muscle activation. | Psychology |
| Transcendental meditation | A specific form of silent, mantra meditation | Mindfulness |
| Western mindfulness-meditation | Mindfulness in the modern Western context. In which, mindfulness and meditation is researched and applied out of religious contexts | Mindfulness, Psychology |
| Zen | A school of Buddhism that originated in China during the Tang dynasty. Zen emphasizes rigorous self-restraint, meditation-practice, insight into the nature of mind | Religion study, Mindfulness |
