# Supplementary material for: The search for scientific meaning in mindfulness research: Insights from a scoping review
Source: PLoS One. 2022 May 4;17(5):e0264924. doi: 10.1371/journal.pone.0264924 (PMC9067662; doi:10.1371/journal.pone.0264924)
Supplement: S2 File — (DOCX) [file pone.0264924.s004.docx]

**Data Availability Statement**

Our submission contains our "minimal data set", which PLOS defines as consisting of the data set used to reach the conclusions drawn in the manuscript with related metadata and methods, and any additional data required to replicate the reported study findings in their entirety. This includes:

1) The values behind the means, standard deviations and other measures reported;

2) The values used to build graphs;

3) The points extracted from images for analysis.
